# Ecological and genomic signatures of the convergent evolution of planktivory in fossil and living reef fishes over deep time

**DOI:** 10.1038/s41467-026-73110-3

**Published:** 2026-05-22

**Authors:** Aintzane Santaquiteria, Dahiana Arcila, Giorgio Carnevale, Melissa Rincon-Sandoval, Fernando Melendez-Vazquez, William T. White, Carole C. Baldwin, Mark W. Westneat, Guillermo Ortí, James C. Tyler, Ricardo Betancur-R

**Affiliations:** 1 Department of Biological Sciences, The George Washington University, Washington, DC USA; 2 Department of Biology, University of Oklahoma, Norman, OK USA; 3 Marine Biology Research Division, Scripps Institution of Oceanography, University of California San Diego, La Jolla, CA USA; 4 Dipartimento di Scienze della Terra, Università degli Studi di Torino, Torino, Italy; 5 CSIRO Australian National Fish Collection, National Research Collections Australia, Hobart, TAS Australia; 6 Department of Vertebrate Zoology, National Museum of Natural History, Smithsonian Institution, Washington, DC USA; 7 Department of Organismal Biology and Anatomy, The University of Chicago, Chicago, IL USA

**Keywords:** Phylogenetics, Comparative genomics

## Abstract

Transitions from benthic to pelagic habitats often coincide with shifts in diet, yet the genomic basis and macroevolutionary dynamics of these changes remain poorly understood. Surgeonfishes and allies (Acanthuriformes), with their rich fossil record, dietary diversity, and compact genomes, provide a powerful model to investigate these transitions across deep time. We integrate genomic data from 80 extant species with morphological data from 32 fossils to construct total-evidence, tip-dated phylogenomic trees and reconstruct ancestral diets and geographic ranges. We identify at least seven independent origins of planktivory and four reversals, with transitions concentrated in the Tethys and Indo-Pacific oceans. Contrary to the view that planktivory represents an evolutionary dead end, we find higher reversal rates and lower extinction in planktivores. Time-dependent models support both climate and phylogenetic effects on planktivorous lineage diversification. Using a newly assembled chromosome-level genome and 44 additional assemblies, we apply phenotype-aware selection models to identify 39 genes under convergent positive selection in planktivorous lineages. Six of these genes show accelerated evolution tied to metabolism and dietary specialization, and also exhibit codon-level parallelism or convergence. Our findings illuminate the ecological, biogeographic, and genomic basis of repeated dietary shifts and challenge assumptions about evolutionary constraints in pelagic environments.

## Abstract (web summary)

Here, the authors present an integrative macroevolutionary analysis of surgeonfishes and their relatives. Using genomic and fossil morphological data to build time-calibrated phylogenies, they infer repeated origins and reversals of planktivory and identify genes under positive selection in planktivorous lineages, primarily associated with metabolic and cellular functions.

## Introduction

Evolutionary convergent adaptations often arise in response to shared ecological pressures or opportunities in similar environments^1^. The shift from benthic (bottom-dwelling) to pelagic (open-water) habitats represents a major ecological transition in aquatic organisms and is frequently accompanied by morphological and trophic specialization^2–4^. Among these, the evolution of a zooplanktivorous diet (planktivory) stands out as a deterministic shift influencing feeding strategies, ecological roles, and functional traits across diverse freshwater and marine fish clades. Notable examples include repeated benthic–pelagic transitions in lake fishes, such as African and Central American cichlids, whitefish, and three-spined sticklebacks, where bottom-feeding benthic forms have evolved into pelagic specialists adapted for planktonic feeding^5–8^. While these cases highlight striking divergence along this environmental axis in relatively recent evolutionary timeframes, similar transitions have also occurred at deeper phylogenetic scales. For example, snappers and fusiliers (Lutjanidae^9^), grunts (Haemulidae^10^), surgeonfishes (Acanthuridae^11–13^), and damselfishes (Pomacentridae^14^) have independently shifted from benthic to pelagic habitats multiple times over the last 60 million years (Ma). Although such ecological transitions are often associated with convergence in ecomorphological traits, recent large-scale studies have found little evidence for strong morphological convergence among reef fish planktivores^15,16^.

Trophic shifts are also common among reef fishes, with dietary identity emerging as a key feature of ecological function and biodiversity patterns in reef ecosystems^17^. Planktivorous fishes, in particular, exhibit unique traits tied to their feeding strategy, such as filter-feeding. Although many species exhibit planktivory in their larval stages, only those with specific adaptations persist as obligate planktivores into adulthood^18^. Marine biodiversity is generally higher in benthic than pelagic environments^19,20^, suggesting that pelagic planktivory may constitute an evolutionary dead end; however, this view is not consistently supported across all groups. Adopting a water column lifestyle may offer short-term advantages, but in the long term, it can create an evolutionary trap due to reduced speciation, elevated extinction, or both^9,21^, particularly in structurally homogeneous pelagic habitats with fewer opportunities for ecological partitioning. While planktivores may initially diversify by exploiting vacant niches with little competition, long-term persistence in a resource-scarce and relatively uniform environment could limit diversification potential^22^. This hypothesis is supported in some groups, such as snappers and fusiliers^9^, yet remains ambiguous in others, including wrasses ^23^ and damselfishes, the latter showing a particularly strong asymmetry through an intermediate dietary ecotype^14^. Understanding these trophic shifts also provides insight into the adaptive capacity and evolutionary responses of organisms to environmental change, including both past paleoclimatic fluctuations and potential future impacts of climate change. Past fluctuations in ocean temperatures have been shown to influence the diversity of marine organisms, with drastic temperature changes followed by either extinction^24–26^ or increasing diversification^27^, but their role in shaping trophic transitions remains unexplored.

While ecological factors are known to be tightly associated with trophic transitions, the genetic mechanisms underlying these shifts remain less understood (but see refs. ^28–30^). A central question is whether similar dietary specializations across lineages arise from convergent changes at the molecular level^31–34^. Genomic innovations have been linked to a range of ecological adaptations, including vision^35^, habitat use^36^, and feeding ecology^37,38^. Previous research, primarily in mammals and insects, suggests that dietary evolution is associated with gene family expansions^39^, changes in gene copy number^40^, or loss of gene function^41^ in pathways related to metabolism. Genes linked to appetite regulation, gastrointestinal function, enzymic digestion, and biosynthesis of fatty acid and cholesterol have been identified in zebrafish^42^ and clownfish^29^. As transitions to the water column are often associated with both a shift to a planktivore diet and morphological changes, previous studies in cichlids, whitefish, or sticklebacks have also identified genes linked to body elongation and caudal fin shape^30,43^, gill raker counts^28,44,45^, and jaw length^46^. However, comparable studies in marine fishes remain sparse, largely due to a lack of high-quality genome assemblies for reef lineages. In parrotfishes, for example, genomic analyses revealed expansions in detoxification genes, likely linked to their highly specialized herbivorous diet and biomineralized teeth^47^.

Reef fishes in the order Acanthuriformes *sensu stricto*^48^, which includes the charismatic unicornfishes, surgeonfishes, and tangs (Acanthuridae), luvars (Luvaridae), and Moorish idols (Zanclidae), offer a unique opportunity to investigate dietary transitions across both extant and fossil species, as well as the genomic underpinnings of these transitions in living taxa. This is facilitated by their relatively small genomes ( ~ 700 Mb), well-documented ecologies, and an exceptional fossil record comprising over 30 species, which provides a temporal framework to examine dietary evolution^49,50^. While previous comparative studies have clarified the role of morphology and diversification dynamics in acanthuriform trophic evolution^11,12,51,52^, integrating fossil and extant data offers deeper insight into the clade’s macroevolutionary and biogeographic history. Two studies—one including 18 fossil taxa subjectively placed from photographic records alongside 41 extant species^50^, and another with 25 fossil taxa placed according to available literature alongside 72 extant species^52^, rather than explicit morphological datasets—proposed that surgeonfishes diversified in two distinct phases: first during the Paleocene–Eocene ( ~ 66–34 Ma), potentially due to ecological release after the Cretaceous–Paleogene (K–Pg) mass extinction, and again in the Oligocene–Miocene ( ~ 23–5 Ma), coinciding with the rise of extensive coral reef systems^53^. Trophically, acanthuriforms are broadly divided into non-planktivores (i.e., herbivores and detritivores feeding on turf algae, macroalgae, or detritus) and planktivores that feed primarily on zooplankton^17^. Although the group is widespread across tropical marine realms, planktivorous species are most common in the Indo-Pacific^12,26,52^. Prior studies have suggested that planktivory evolved at least five times independently within surgeonfishes^11,12^, however, these inferences were based on limited phylogenetic resolution from mitochondrial and nuclear markers and did not incorporate fossil data. Moreover, the genomic basis of trophic transitions in marine fishes remains poorly understood in a macroevolutionary context.

Here, we investigated the tempo and mode of planktivory evolution in fossil and extant acanthuriforms. Specifically, we (1) estimated the timing, frequency, and geographic context of dietary shifts; (2) tested whether planktivory represented an evolutionary dead end; (3) assessed correlations between trophic transitions and paleoclimatic trends; and (4) identified candidate genes under positive selection associated with transitions to planktivory. We constructed time-calibrated phylogenies using a total-evidence dating approach that integrated genome-wide data from nearly 1000 loci in 80 extant species with 107 morphological characters scored across 32 fossil and 19 extant taxa. From these phylogenies, we reconstructed ancestral diets and geographic ranges, estimated diet-dependent diversification rates, and evaluated the impact of ancient ocean temperatures on trophic shifts. We also tested the hypothesis that acanthuriform trophic shifts have been shaped by past geological and climatic events and the extent to which transitions to a planktivorous diet represent an evolutionary dead end. To examine the genomic basis of planktivory, we generated a chromosome-level genome assembly for a surgeonfish representative and short-read assemblies for 44 additional acanthuriform species. Using phylogenetic genotype-to-phenotype (PhyloG2P) analyses^54^, we identified genes under positive selection that have convergently evolved across planktivorous lineages and along branches coinciding with this dietary transition.

## Results and discussion

### Phylogenomic total-evidence dating resolves acanthuriform relationships and reveals post-K–Pg diversification

To infer acanthuriform relationships, we assembled three complementary datasets. The morphology-only matrix includes 107 characters newly scored for 32 fossil and 19 extant species, along with 5 extant outgroups. The molecular-only matrix contains 80 ingroup species (representing approximately 93% of the group’s diversity) and 9 outgroups. Of these, 65 species have data for 998 “FishLife” exon markers^55^, which also incorporate 22 legacy markers, whereas 24 species include data only for legacy markers. Finally, we created a combined dataset that integrates both morphological and molecular data. Using several complementary methods, we consistently retrieved the same family-level patterns of relationship from the genomic data. Concatenation-based maximum-likelihood trees built in RAxML v.8.2.11 (ref. ^56^; Supplementary Figs. 1 and 2), as well as coalescent trees inferred with ASTRAL-III (ref. ^57^; Supplementary Figs. 3 and 4), all resolve one well-supported acanthuriform clade in which Luvaridae branches first, followed by the sister pair Zanclidae + Acanthuridae. The combined morphology + molecular dataset yields the same topology (Supplementary Fig. 7). By contrast, morphology-only trees generated with TNT v1.5^58^ and RAxML split Zanclidae and Acanthuridae and reposition Luvaridae, indicating that skeletal traits alone can be misleading at this phylogenetic depth (Supplementary Fig. 6). Specifically, *Luvarus* is grouped with the largely planktivorous *Naso*, a pattern that may reflect convergence associated with planktivory and illustrates how morphology alone can obscure true evolutionary relationships, reinforcing the importance of incorporating genomic data. Most genera remain monophyletic; the main exceptions are *Ctenochaetus* and *Acanthurus* (both surgeonfishes) consistent with previous studies^52,59,60^ and the fossil genus *Tylerichthys*. Gene-concordance analyses indicate that individual genes overwhelmingly support the molecular topology, reinforcing the robustness of the genomic signal (see Supplementary Fig. 5).

**Fig. 1:**
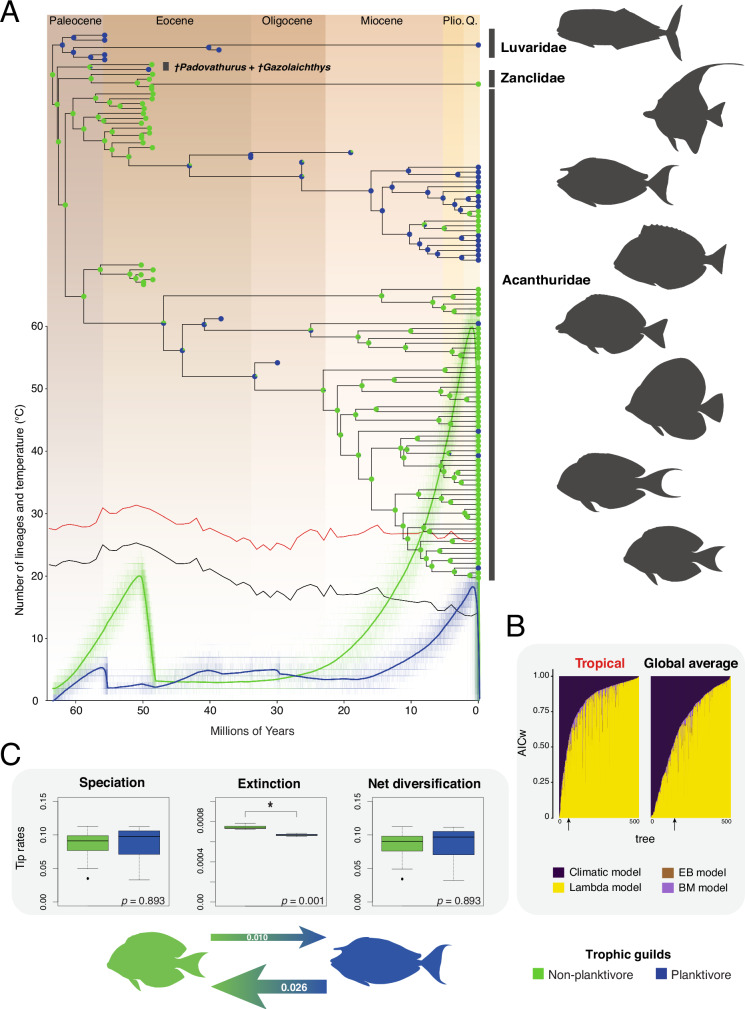
Diversification of fossil and extant acanthuriforms and evolutionary origins of planktivory. **A** Ancestral diet reconstruction based on a posterior distribution of 500 trees (999 loci) from Scheme 1 trees for 112 species (32 fossil and 80 extant) reveals multiple transitions between non-planktivorous and planktivorous diets across the acanthuriform phylogeny. Lineage-through time plot for each trophic guild show two major waves of diversification: one during the Paleocene–early Eocene and another in the early Miocene. The darker lines indicate the mean number of lineages over time for each trophic guild, and color indicates dietary strategy. **B** Ocean temperatures (purple) and phylogenetic signal (yellow) show cofounding effects in the evolution of planktivory. AIC weights are shown for climate-dependent (curves for tropical in red and global average temperature in black, illustrated in (**A**)) and independent models based on the 500 alternative trees as well as the MCC tree (indicated with a black arrow). **C** Model-averaged tip rates estimated with HiSSE show similar net diversification rates between trophic guilds, although non-planktivores experienced higher extinction rates. Box plots summarize speciation, extinction, and net diversification tip rates for extant species under the MCC tree, comparing non-planktivores (*n* = 61) and planktivores (*n* = 19). Box plots indicate median (middle line), 25th, 75th percentile (box), and 5th and 95th percentile (whiskers) as well as outliers (single points). *P*-values calculated using two-sided phylogenetic ANOVA with Bonferroni correction are shown at the bottom right of each panel. * *p*-value < 0.05. Arrows between fish silhouettes represent transition rates between dietary strategies estimated with HiSSE. Plio. Pliocene, Q. Quaternary, EB early burst, BM Brownian motion. Fish silhouettes were obtained from R package *fishualize* (https://github.com/nschiett/fishualize/tree/master). Source data are provided as a Source Data file.

We estimated divergence times using a Bayesian tip-dating (total-evidence) framework under the Fossilized Birth–Death model in MrBayes v.3.2.7a^61^. To account for topological uncertainty, we assembled five independent datasets integrating the 107 morphological characters and ~200 non-overlapping molecular markers for 112 taxa (80 living, 32 fossil; Supplementary Fig. 8 and Supplementary Table 2). Each tip-dating analysis ran for over a year to ensure convergence (effective sampling size or estimated sample size (ESS) values > 200, Supplementary Table 4). From each of the five runs, we retained 100 post-burn-in trees, yielding a total of 500 trees used to account for tree uncertainty in downstream comparative analyses. Two Eocene fossils, †*Gazolaichthys vestenanovae* and †*Padovathurus gaudryi*, previously assigned to Acanthuridae^52^, were placed within that family in our morphology-only phylogeny (Supplementary Fig. 6) but fell outside Acanthuridae in the combined morphological + molecular analyses, instead forming the sister group to Zanclidae and Acanthuridae. Given anatomical evidence supporting the latter arrangement^62^, we present comparative results based on this topology (the two Eocene fossils sister to Zanclidae + Acanthuridae: Scheme 1 hereafter), with alternative results (the two Eocene fossils sister to Acanthuridae: Scheme 2 hereafter) reported in the Supplementary Information (Supplementary Figs. 10, 14, 16, 17, 19–21 and Supplementary Data 3–5). The resulting trees (Fig. 1A and Supplementary Fig. 9) were topologically stable and broadly consistent with previous molecular^11,48,51,52,59,60,63^ and morphological^64^ studies.

According to the time-calibrated trees, acanthuriforms originated in the Paleocene around 63.6 Ma based on the maximum clade credibility (MCC) tree (95% highest posterior density or HPD: 64–63.3 Ma), shortly after the K–Pg extinction. This estimate is notably younger than previous gene-only analyses, which placed the origin at approximately 80 Ma^52^. Luvaridae originated at 62.1 Ma, followed by Acanthuridae (unicornfishes, surgeonfishes, and tangs) at 61.7 Ma, and Zanclidae at 57.8 Ma. Within acanthurids, most fossil genera (e.g., *Tauichthys*, *Proacanthurus*) arose between 60–50 Ma, while extant genera diversified later: *Naso* at 26.4 Ma, *Paracanthurus* at 25.0 Ma, *Acanthurus* at 23.2 Ma, *Zebrasoma* at 17.9 Ma, and the clade uniting *Acanthurus* and *Ctenochaetus* at 11.6 Ma. These results confirm a post-K–Pg radiation of major lineages (Fig. 1A) and emphasize the value of integrating fossil and molecular evidence for reconstructing evolutionary history^65^.

### Multiple gains and losses of planktivory trace to early Tethyan origins and subsequent Indo-Pacific spread

The recurrent phenomenon of trophic transitions from an herbivorous/detritivorous diet to a planktivorous one is not limited to surgeonfishes, unicornfishes, and luvars, but extends to various other reef fish groups, including damselfishes, groupers, snappers, triggerfishes, and wrasses^18^, as well as freshwater species flocks like cichlids, whitefish, and stickleback^5,6,66^. With the newly inferred time trees including fossil and living acanthuriform species, we reconstructed their diets to investigate the number of times planktivory evolved, as well as the regions and timing of these transitions. For extant species, we collected diet data from the primary literature^11,12^ and FishBase^67^. For fossils, we inferred diet based on paleoenvironmental information rather than tooth morphology, a common approach^68,69^ that can be misleading in this group. In extant species, both planktivorous and non-planktivorous fishes often possess multi-denticulate or conical teeth (Supplementary Fig. 11), limiting the reliability of dental characters for reconstructing trophic ecology. Fossils found in oceanic sediments were coded as planktivores, while those from limestones were coded as non-planktivores^49,70^. Ancestral diet reconstruction using the “make.simmap” function in the R package *phytools*^71^ (SIMMAP hereafter) was conducted under the best-fit equal-rates (ER) model for both phylogenies; ER was marginally favored for the extant-only tree (AICw = 0.51 vs. 0.48 for all rates different or ARD) and similarly for the tree including fossils (AICw = 0.55 vs. 0.45 for ARD). Under both models, we inferred six independent transitions to planktivory in the extant-only tree (Supplementary Figs. 12 and 15). When fossils were incorporated, the number of independent origins increased to at least seven (range: 7–9 across posterior distribution [PD] trees). These included one origin (1–3 in PD trees) in extinct lineages, three (2–3) in stem clades that include both fossil and extant species, and three (3–4) in extant-only clades (Fig. 1A and Supplementary Figs. 13 and 16). We also identified three reversals to non-planktivory in the extant-only tree, and four (3–5) when fossils were included. These trophic transitions span most of acanthuriform history, occurring between 62.1 and 3.6 Ma (Fig. 1A).

**Fig. 2:**
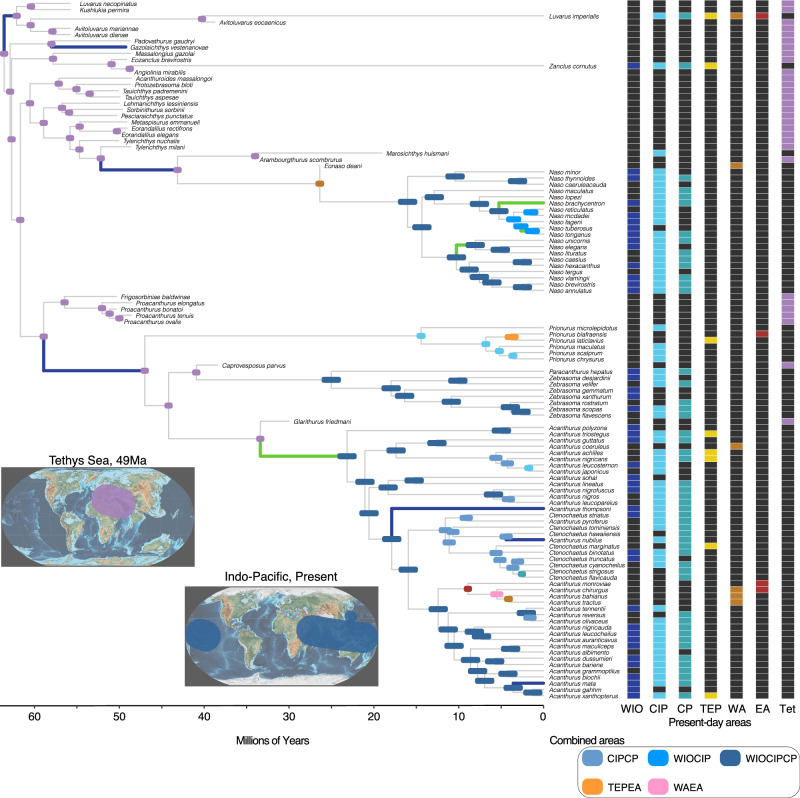
Historical biogeography of acanthuriforms and the geography of trophic transitions. Early transitions to planktivory occurred in the ancient Tethys Sea, while more recent ones took place in the Indo-Pacific Ocean. Ancestral ranges were estimated in *BioGeoBEARS* using the best-supported biogeographic model (BAYAREALIKE+w) applied to 20 trees subsampled across the five subsets from Scheme 1 and using the MCC tree as fixed topology. Boxes at internal nodes are color-coded by area or combination of areas with the highest likelihood. Boxes at the tips are colored according to the present-day distribution of each species. Branches undergoing a transition to planktivory are indicated in blue, while those reverting to non-planktivory are shown in green. The paleomaps (obtained from GPlates, Global_Scotese, https://www.gplates.org/) illustrate the location of the Tethys Sea at 49 million years ago (Ma), and the Indo-Pacific at present-day. WIO Western Indian Ocean, CIP Central Indo-Pacific, CP Central Pacific, TEP Tropical Eastern Pacific, WA Western Atlantic, EA Eastern Atlantic, Tet Tethys Sea.

To contextualize these transitions geographically, we conducted ancestral range reconstruction analyses in the R package *BioGeoBEARS*^72^. We evaluated 12 biogeographic models and ran analyses using either 16^73^ or 12 Ma^74–76^ as the final closure of the Tethys Seaway, with both timepoints yielding similar results (see Fig. 2 and Supplementary Fig. 18). Under the best-fit model (BAYAREALIKE+w; Supplementary Table 5), acanthuriforms were inferred to have originated in the Tethys Sea (Tet). From there, five lineages (from all genera except *Ctenochaetus*) expanded into the Indo-Pacific, and one lineage (*Eonaso*) dispersed into the Western Atlantic (WA). Subsequent dispersal from the Indo-Pacific included at least six events to the Tropical Eastern Pacific (TEP), one to the WA, and two to the Eastern Atlantic (EA). Two additional lineages crossed the Atlantic: (i) the ancestor of *Acanthurus chirurgus*, *A. bahianus*, and *A. tractus*, which dispersed from the EA to the WA; and (ii) an ancestral *Prionurus biafraensis* lineage that moved from the TEP to the WA. This biogeographic history aligns with a previous study on surgeonfishes and many other circumglobal shallow marine fish groups^9,52,77,78^. Although planktivory has evolved repeatedly across the world’s oceans, in acanthuriforms the earliest transitions (4–5 events) occurred in the Tethys Sea. More recent transitions (3–4 events) took place predominantly in the Indo-Pacific, particularly in the Western Indian Ocean (WIO), Central Indo-Pacific (CIP), and Central Pacific (CP) regions, characterized by complex reef topography that offers predator refuge and promotes high productivity through dynamic water flow^79,80^. No transitions were detected in the WA, EA, or TEP (Fig. 2). These results highlight the repeated and spatially structured evolution of planktivory in acanthuriforms, shaped by both deep-time biogeography and regional ecological opportunity.

### No evolutionary dead end associated with the evolution of planktivory

Planktivory is considered the principal evolutionary destination among trophic transitions in reef fishes^17^, often assumed to represent a terminal trophic state. This expectation forms the basis of the evolutionary dead end hypothesis^81^, which in our system yields two testable predictions: (i) planktivorous lineages should exhibit lower diversification (either through reduced speciation, elevated extinction, or both) relative to non-planktivorous lineages; and (ii) trophic transitions should be directionally biased, with shifts into planktivory occurring more frequently than reversals to non-planktivory. To evaluate these predictions, we estimated diversification and transition rates between non-planktivory and planktivory by conducting state-dependent speciation and extinction (SSE) analyses using *HiSSE*^82^ on the MCC tree, including only extant species (80 tips). To incorporate model uncertainty, we model-averaged rates for all tips and nodes across five models using AIC weights (Supplementary Table 6).

Our results indicate that both net diversification (*p* = 0.893 for the MCC tree and 0.001–0.995 for PD trees) and speciation rates (*p* = 0.893 for the MCC tree and 0.001–0.996 for PD trees) are similar between non-planktivorous and planktivorous species, while extinction rates are slightly lower in planktivores (*p* = 0.001 for the MCC tree and 0.001–0.895 for PD trees; Fig. 1C and Supplementary Table 7). Transition rates from non-planktivory to planktivory (0.010 for the MCC tree and 0.007–0.011 for PD trees) were lower than the reverse (0.026 for the MCC tree and 0.013–0.034 for PD trees; Supplementary Table 7). When we compared these results with transition rates from ancestral diet reconstructions, we found them to be highly consistent. Including fossils, SIMMAP estimated an ER rate of 0.015, with ARD rates of 0.012 from non-planktivory to planktivory and 0.025 for the reverse; excluding fossils, the ER rate was 0.012, with ARD rates of 0.010 and 0.025, respectively. These values closely match the HiSSE estimates (0.010 and 0.026), indicating that differences between approaches reflect model structure and sensitivity rather than conflicting evolutionary signals. Although the number of transitions from non-planktivory to planktivory has exceeded reversals over the course of acanthuriforms’ evolutionary history, it is noteworthy that planktivorous lineages are generally younger than their non-planktivorous counterparts. Consequently, younger lineages, such as *Naso* (unicornfishes), have experienced reversals to the ancestral condition (non-planktivory), leading to higher rates of transitions from planktivory to non-planktivory compared to the reverse direction. Some species in this genus, especially *N. brevirostris* and *N. vlamingii*, feed on benthic macroalgae as juveniles and become planktivorous later in life^83,84^, highlighting an exceptional dietary lability in *Naso*. These reversals occurred alongside similar diversification rates among both trophic guilds, contradicting our prediction based on an evolutionary dead end scenario. While planktivory is often considered an evolutionary destination^17^, some unicornfishes revert to their ancestral trophic states without further dietary specialization, representing a notable exception to this expectation. The transition to a planktivorous diet often coincides with a shift from bottom-dwelling to mid-water habitats, and it is conceivable that reliance on a specific dietary resource, especially under changing environmental conditions or competitive pressures, may constrain the adaptive potential of planktivorous species. Together, these findings suggest that planktivory does not represent an evolutionary dead end in acanthuriforms, but, rather, a dynamic state with the potential for ecological and evolutionary reversibility.

### Paleoclimate effects on the origin of planktivory

We tested whether shifts in ocean temperatures over the last 64 million years influenced the trophic diversification in acanthuriforms, with a particular emphasis on the emergence of planktivorous lineages. During this period, global temperatures fluctuated significantly^85^, driven by paleogeographic, tectonic, and oceanographic changes^86^. To explore these patterns, we generated lineage-through-time (LTT) plots for planktivores and non-planktivores using 500 tip-dated trees in *phytools*^71^, and compared them with global average and tropical temperature curves^86^ (Fig. 1A and Supplementary Figs. 14 and 17). We then statistically assessed correlations by leveraging a time-dependent threshold analysis, fitting various paleoclimate-dependent and -independent diversification models^87^ to the MCC tree, incorporating species’ dietary guilds and using both temperature curves.

These analyses revealed that acanthuriforms gradually started diversifying during the Paleocene and early Eocene (66–50 Ma), particularly benthic lineages whose trophic ecology (based on inference from extant relatives^11^) might have involved filamentous or macrophytic algae as well as detritus and microbes. The early Eocene period (56–48 Ma) marked a significant phase of elevated temperatures^88^, with ocean temperatures surging by 6 °C^85^. This warm interval encompassed both the Paleocene–Eocene Thermal Maximum (~56 Ma) and the Early Eocene Climatic Optimum (EECO; 53–49 Ma)^89^, the latter coinciding with Bolca (50.5–48.5 Ma) in the Tethy Sea, a rich site of reef fish fossils^49,90^. This climatic phase has been linked to elevated extinction rates in some marine fishes^26^ and foraminifera^91^, coincident with a coral reef crisis triggered by ocean acidification and a consequent decline in reef growth^92^. Several benthic-feeding acanthuriform lineages are restricted to this interval in the fossil record (Fig. 1A^49,50^), suggesting a contraction or turnover of benthic trophic lineages during this time. In contrast, zooplanktivorous species in the water column appear to have been less affected. Following the EECO ( ~ 49 Ma), global cooling set in and was accompanied by increased diversity in planktonic groups such as foraminifera, diatoms, and radiolarians^93,94^. This shift may have created new ecological opportunities for planktivores, facilitating their diversification, as supported by our results showing elevated origination rates for planktivorous lineages during the middle to late Eocene (44–30 Ma; Fig. 1A, B). However, it is important to recognize that strong taphonomic biases, such as the dominance of non-planktivorous acanthuriform fossils in Bolca deposits^49,90^, may exaggerate the apparent link between temperature shifts and extinction patterns.

A second wave of diversification occurred in the early Miocene ( ~ 20 Ma), when acanthuriform planktivores, alongside other reef fishes, increased in lineage diversity, although non-planktivores exhibited a steeper diversification slope (Fig. 1A). This diversification coincided with new reef configurations, coral diversification, and major changes in Indo-Pacific Ocean circulation^12,17,79,95,96^. Additionally, persistently warm tropical temperatures over the past ~20 Ma^85,97^ increased algal turf cover, boosting food availability and supporting higher abundances of herbivorous reef fishes^98^.

Although we observe a potential temporal correlation between the evolution of planktivory and major climatic transitions, our model fitting analyses indicate split support between climate-dependent and climate-independent models. Based on the MCC tree, the phylogenetic signal (lambda) model (AICw = 0.55 vs. 0.51) and the climate-dependent model (AICw = 0.44 vs. 0.48) both fit better than climate-independent models such as Brownian motion (AICw = 0.005 vs. 0.004) and early burst (AICw = 0.002 vs. 0.002; Fig. 1B and Supplementary Table 8). Across the 500 posterior trees, however, the lambda model received slightly stronger support, with AICw averages of 0.63 (global temperatures) and 0.82 (tropical temperatures), compared to 0.34 and 0.15, respectively, for the climate-dependent model (Fig. 1B and Supplementary Table 8). Very similar results were obtained under Scheme 2 fossil placements (Supplementary Figs. 14, 17, and 21 and Supplementary Data 3–5). Overall, these findings suggest that ancient climate shifts and lineage-specific evolutionary history both likely contributed to the timing and tempo of planktivory diversification in acanthuriform fishes.

### Signatures of convergence in positive selection following transitions to planktivory

Understanding whether trophic transition convergence is mirrored at the molecular level (i.e., through repeated use of the same genes or pathways) could offer critical insights into the genetic mechanisms underlying adaptation to planktivory. To explore this, we investigated genome-wide signatures of positive selection associated with dietary transitions in acanthuriforms using PhyloG2P approaches. First, we sequenced, assembled, and annotated a chromosome-level reference genome of *A. chirurgus* (doctorfish tang, Acanthuridae) using PacBio long-reads, Hi-C, and IsoSeq sequencing technologies. This resulted in a high-quality phased genome with 15 putative chromosomes for each haplotype. Haplotype 1 has a size of 776.87 Mb, with scaffold N50 of 47.48 Mb, while haplotype 2 has a size of 664.96 Mb, scaffold N50 of 47.66 Mb (Supplementary Fig. 22). We assessed the genome completeness in BUSCO^99,100^ based on the Actinopterygii database (odb12). Before annotation, BUSCO showed a completeness score of 98.1% (Supplementary Fig. 23). When BUSCO was run on the annotated genome and predicted 23,548 protein-coding genes, the completeness was 85.7%. Second, we sequenced a total of 48 short-read genomes through Illumina. We assembled them in MaSuRCA v.4.0.8^101^ and enhanced scaffolding of each genome by mapping to the reference genome in RagTag v.2.1.0^102^ to reorder, reorient, and fill gaps. After quality control and removal of duplicated *A. chirurgus*, we retained 44 assemblies, whose BUSCO completeness scores ranged from 67 to 97.6% (Supplementary Fig. 23).

**Fig. 3:**
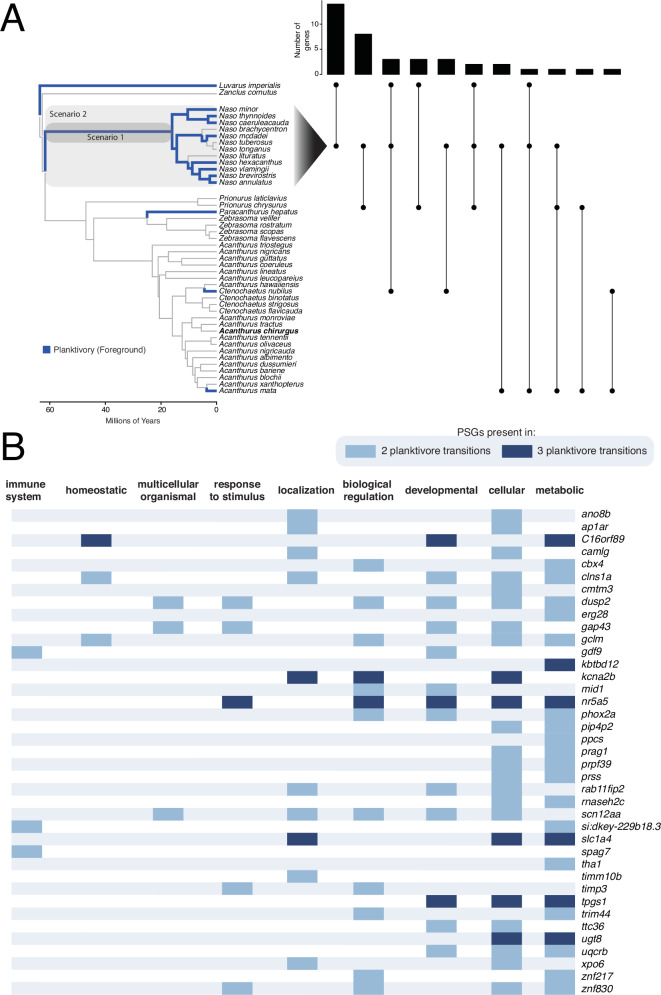
Convergent positive selection associated with transitions to planktivory. **A** Schematic representation of the aBSREL analysis used to identify positively selected genes (PSGs), with branches associated with transitions to planktivory shown in blue. For the genus *Naso*, two scenarios were analyzed: Scenario 1 captures selection occurring during the initial transition to planktivory (stem branch only), while Scenario 2 includes both stem and crown branches, capturing selection during and after the transition. The species for which we generated a reference genome (*Acanthurus chirurgus*) is shown in bold. The UpSet plot (right) illustrates the number of PSGs (combing both scenarios) shared across planktivorous lineages, with *Naso* exhibiting the highest degree of overlap, likely reflecting its extended evolutionary history as a planktivore (see main text). Source data are provided as a Source Data file. **B** Table listing the 39 PSGs convergently evolving in at least two lineages, along with their associated biological processes based on Gene Ontology annotations. Metabolic and cellular processes were the most frequently represented functional categories. Light blue cells indicate PSGs shared by two lineages; dark blue indicates convergence across three. Cell differentiation and growth function are grouped under the category “developmental processes”.

To detect genes under positive selection associated with planktivory, we used the dN/dS ratio as implemented in HyPhy^103^. We analyzed 3926 single-copy orthologs (2542 derived from the annotated reference genome and 1384 from the BUSCO databases) filtered through OrthoFinder and rigorous quality control procedures (see “Methods”). We targeted selection along the five branches where transitions to planktivory were inferred based on ancestral diet reconstructions (Fig. 3). Our approach combined two complementary methods: the adaptive Branch-Site Random Effects Likelihood model (aBSREL^104^), which detects selection on specific branches, and BUSTED-Phenotype (BUSTED-PH^105^), which compares selection pressures between foreground (planktivore) and background (non-planktivore) lineages (Supplementary Fig. 23). This two-step filtering strategy allowed us to identify positively selected genes (PSGs) evolving under selection specifically during or following transitions to planktivory. After correcting for multiple testing using a false discovery rate threshold (*p* < 0.05) and combining both aBSREL and BUSTED-PH results, we identified a total of 302 PSGs along branches associated with planktivory transitions (Supplementary Data 4). Among this, 39 convergently underwent positive selection in at least two lineages (Fig. 3 and Supplementary Data 5).

Among the lineages analyzed, *Naso* exhibited the highest number of shared PSGs with other planktivorous taxa (Fig. 3A). However, rather than indicating stronger molecular convergence per se, this pattern likely reflects the extended evolutionary duration of planktivory in *Naso*, which includes multiple species and relatively long branches, thereby providing more opportunities for positive selection to accumulate. Similarly, elevated convergence observed between *Naso* and *Luvarus*, or *Naso* and *Paracanthurus hepatus*, may result from this same “age effect,” as both *Luvarus* and *P. hepatus* represent some of the most ancient, long-branched planktivore lineages. This interpretation is consistent with recent findings that emphasize the importance of time and clade age in shaping patterns of molecular convergence across ecological transitions^38,45^.

Gene Ontology (GO) enrichment analyses based on the PANTHER^106^ and ZFIN^107^ databases revealed 10 biological processes associated with these PSGs (Fig. 3B). The most represented categories were metabolic (24 genes) and cellular processes (24 genes), followed by developmental/growth/cell differentiation processes (13 genes) and biological regulation (12 genes). Other notable functions included localization (10 genes) and response to stimulus (5 genes). Less frequent categories included multicellular organismal processes (3 genes), homeostatic processes (3 genes), and immune system processes (3 genes). All in all, these results suggest that dietary transitions to planktivory involved diverse physiologic and developmental pathways, rather than a single convergent genetic mechanism.

### Accelerated molecular evolution in metabolic genes linked to transitions to planktivory

**Fig. 4:**
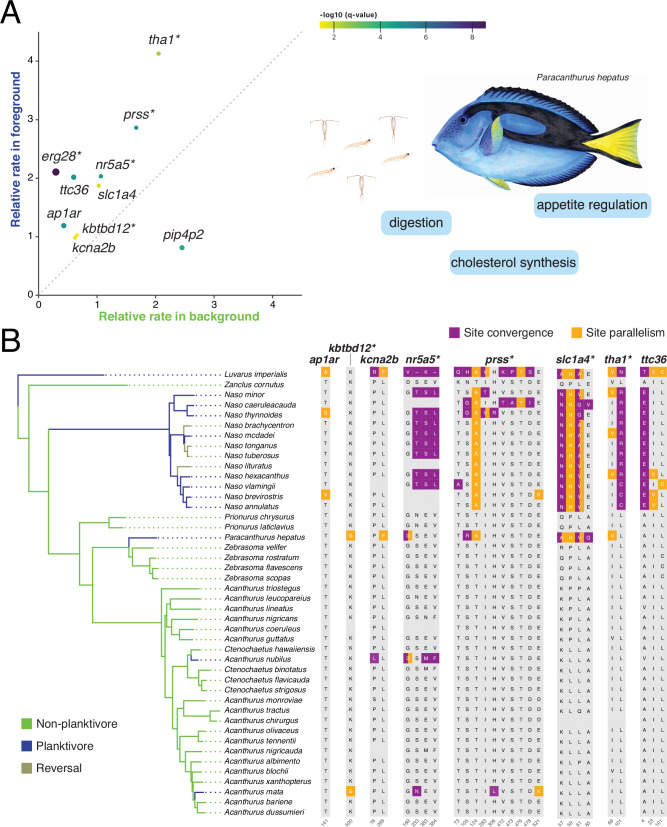
Evolutionary rate shifts and convergence patterns in positively selected genes, including parallel and convergent evolution at selected sites. **A** The scatterplot shows relative amino acid substitution rates in foreground (planktivorous) versus background (non-planktivorous) branches for each convergently evolved positively selected gene (PSG). Dots above the diagonal indicate acceleration in planktivores. Dot color represents the FDR-corrected *q*-value (as –log₁₀(*q*)); bubble size reflects the magnitude of rate shift [max(foreground/background, background/foreground)]. The asterisk following the gene name denotes that the gene is involved in metabolic processes. Key metabolic functions linked to the accelerated genes are highlighted in blue. The Palette surgeonfish (*Paracanthurus hepatus*) represents an example of a planktivore species. The illustrations of the surgeonfish, copepod, and krill were done by Enara Santaquiteria. Source data are provided as a Source Data file. **B** The phylogeny maps sites under episodic positive selection (identified with MEME), highlighting codons showing parallel evolution (same amino acid change across lineages, in orange) and convergent evolution (different amino acid changes at the same site, in purple). Sites with both colors suggest contributions from both mechanisms (e.g., when three lineages are involved). Codon positions are shown for each labeled site.

While genes related to metabolism have been implicated in dietary adaptations across various fish species, such as zebrafish^42^, goldfish^108^, parrotfish^47^, and clownfish^29^, it remains unclear whether these metabolic genes also exhibit signatures of rapid evolution in association with repeated shifts to planktivory. To address this, we tested whether the 39 convergently evolved PSGs showed accelerated or decelerated evolution in planktivorous lineages by estimating the relative evolutionary rates (RERs) in HyPhy. We detected ten genes with rate shifts associated with trophic transitions: nine exhibiting accelerated rates and one showing decelerated rates (Fig. 4 and Supplementary Data 5). Six of the accelerated genes are associated with metabolic processes and are likely involved in the digestion of zooplankton and small crustaceans, key components of a planktivorous diet (Figs. 3 and 4). Notably, *erg28* (ergosterol biosynthesis 28), a gene involved in cholesterol biosynthesis and membrane homeostasis, exhibited the strongest signal of evolutionary acceleration, largely driven by *Naso vlamingii*, *N. brevirostris*, and *N. annulatus*^109^. Selection on *erg28* may reflect adaptations to optimize energy metabolism and nutrient absorption under ecological pressures such as prey availability or predation risk. Similarly, *nr5a5* (nuclear receptor subfamily 5, group A, member 5), a regulator of cholesterol metabolism and steroidogenesis expressed in the digestive tract and linked to appetite regulation, may reflect adaptation to a lipid-rich zooplanktonic diet^110^, with acceleration likely influenced by species such as *Acanthurus nubilus*, *Luvarus imperialis*, *Naso minor*, and *N. thynnoides*.

Other accelerated genes include *prss*, a serine protease involved in protein digestion and expressed in the digestive system^29^, which showed signals of selection in three *Naso* species. *Slc1a4* (solute carrier family 1 member 4), a transporter of neutral amino acids like serine and alanine, abundant in zooplankton and expressed in the gut^111,112^, was accelerated in *N. mcdadei* and *Paracanthurus hepatus*. *Tha1* (threonine aldolase 1), involved in amino acid metabolism and gluconeogenesis, is accelerated in *N. hexacanthus*, while *kbtbd12* (kelch repeat and BTB domain containing 12), linked to protein ubiquitination and energy regulation, showed acceleration in *N. vlamingii* and *N. annulatus*^107^. *Kcna2b* (potassium voltage-gated channel, shaker-related subfamily, member 2b), though unrelated to metabolism, plays a role in neuronal signaling and action potential propagation and was accelerated in *Luvarus*^107^. *Ttc36* (tetratricopeptide repeat domain 36), involved in developmental processes such as cilium assembly and otolith morphogenesis, also showed acceleration in *Luvarus* and *N. vlamingii*, despite no known digestive role^107^. Lastly, *ap1ar* (AP-1 complex-associated regulatory protein), involved in intracellular transport and vesicle trafficking (vesicle targeting from trans-Golgi network to endosomes), is accelerated in *N. brevirostris*. The only gene with a significant deceleration was *pip4p2* (Phosphatidylinositol-4,5-Bisphosphate 4-Phosphatase 2), involved in lipid signaling, energy use, and nutrient uptake; this pattern was driven by *A. nubilus*, *P. hepatus*, and *N. minor*^107^. Altogether, the digestive functions and expression profiles of these genes highlight their likely role in facilitating dietary specialization during repeated evolutionary transitions to planktivory.

We further examined whether any of the nine accelerated genes exhibited evidence of episodic diversifying selection at specific codons, and whether these changes reflected convergence or parallelism. Using MEME^113^ we identified 93 positively selected sites across these genes, with counts ranging from 19 in *prss* to 3 in *erg28* (Supplementary Data 5). While many sites were flagged, only a subset showed clear evidence of convergence, parallelism, or both—potentially contributing to the repeated evolution of planktivory (notably, none were detected in *erg28*; Fig. 4). Site convergence refers to independent planktivory transitions resulting in different amino acid substitutions at the same codon, whereas site parallelism involves identical amino acid changes across transitions. Several genes showed both patterns. For example, *kcna2b* exhibited site convergence between *Luvarus imperialis* and *Acanthurus nubilus*, and site parallelism between *L. imperialis* and *Paracanthurus hepatus*. In *prss*, *slc1a4*, *ttc36*, and *tha1*, both convergence and parallelism were observed across *L. imperialis*, members of the genus *Naso*, and in some cases *P. hepatus*. Notably, *prss* and *nr5a5* had the highest number of positively selected sites (6 and 5, respectively), most of which reflected site convergence. These results suggest that episodic diversifying selection has significantly influenced the evolution of these genes, with both convergent and parallel mechanisms shaping their sequence variation, potentially as adaptations to specific dietary niches or environmental pressures. More broadly, our findings highlight how macroevolutionary patterns of trophic specialization in acanthuriforms emerge from a dynamic interplay of ecological filtering, mutation bias, and lineage-specific evolutionary history, funneling adaptation along repeatable yet historically contingent molecular trajectories^34,45,114^.

### No statistical associations between repetitive elements and planktivory

The quantity of repetitive DNA in a genome correlates with genome size^115,116^, and shifts in repeat content have been linked to adaptations to new environments^117–119^. For example, bursts of transposable elements (TEs) have been implicated in the diversification of clownfishes through their mutualistic interactions with sea anemones^120^. However, investigations specifically examining the relationship between repetitive genomic elements and dietary patterns remain lacking. To address this gap, we assessed the association between transposable element (TE) content and trophic guild in acanthuriforms. First, we categorized repeat elements in each genome into ten distinct classes or subfamilies. In the reference genome, repetitive sequences comprised 16.39% of the assembly, while short-read assemblies showed a range of 13.8–21.3% (Supplementary Fig. 24). Second, we performed a phylogenetic ANOVA using *phytools*^71^ to test for differences in TE content between trophic guilds. We found no significant association between overall TE content and diet (*p* = 0.989; Supplementary Fig. 24). Similarly, none of the individual repeat categories showed significant correlations with trophic guild (*p* = 0.178–0.975; Supplementary Fig. 25). These results indicate that repetitive elements do not systematically vary with dietary transitions to planktivory in acanthuriforms. This pattern aligns with findings from other recent studies, such as in catfishes^45^ and teleosts more broadly^121^, which similarly report weak or inconsistent associations between repeat content and ecological or behavioral traits. While TE expansions can occasionally correlate with environmental shifts or significant life history changes, such as mutualisms^120^, this pattern is not universal. Repeat expansions may occur under relaxed selection or during environmental stress, but they are not a consistent feature of ecological diversification^122^. In planktivory, dietary transitions appear driven primarily by selection on coding regions, with no detectable parallel changes in TE abundance. Although we cannot rule out the potential role of regulatory evolution, our results suggest that repetitive elements have played a minimal role in the trophic diversification of acanthuriform fishes.

## Final remarks

This study provides a comprehensive view of the evolutionary dynamics of trophic transitions in both fossil and extant acanthuriform fishes by examining their associations with biogeographic patterns, diversification rates, paleoclimatic changes, and genomic signatures. By integrating morphological and genome-wide data, we inferred the most comprehensive tip-dated phylogeny to date for the group, accommodating uncertainties in both topology and divergence time estimates for downstream analyses. We found that planktivory evolved independently at least seven times, including four early Cenozoic transitions in now-extinct lineages. Notably, our biogeographic reconstructions reveal that early transitions occurred in the ancient Tethy Sea, while more recent ones are concentrated in the reef-rich Indo-Pacific, highlighting the role of spatiotemporal ecological opportunity in shaping trophic evolution. Similar net diversification rates between non-planktivores and planktivores, coupled with the fact that transition rates to planktivory are lower than reversals, suggest that a planktivorous lifestyle is not an evolutionary dead end in this group. Our findings also suggest that dietary shifts reflect both paleoclimatic fluctuations and lineage-specific histories, with temporal patterns aligning planktivore diversification with cooler intervals and benthic herbivore diversification with warmer periods, while models also support strong phylogenetic influences.

Our genomic analyses reveal that repeated transitions to planktivory are accompanied not only by convergent adaptations but also by shared molecular signatures of positive selection and accelerated evolution, particularly in genes related to metabolism, digestion, and nutrient uptake. While both site convergence and parallelism were observed, the molecular pathways involved appear shaped by a complex interplay of evolutionary history, lineage-specific context, and time (lineage age). These findings emphasize that while convergent ecological pressures may prompt repeatable genomic responses, the molecular routes taken remain partly lineage-specific. Despite providing insights into the ecological and genomic basis of planktivory transitions in reef fishes, our PhyloG2P analyses have limitations. Notably, our focus on protein-coding genes overlooks regulatory elements or noncoding regions involved in dietary adaptation, and we did not identify any PSGs shared across all planktivory transitions. Future research can leverage the annotated reference genome generated here, alongside additional chromosome-level assemblies, to explore genomic features (e.g., rate acceleration in regulatory regions; genome expansions and contractions) that may contribute to trophic specializations, including genes like taste receptor^41^. Integrating skull shape data^123–125^ may also further reveal ecomorphological patterns and help link the genotype to the phenotype.

Together, our findings enhance understanding of the ecological and genetic factors shaping dietary evolution in acanthuriforms and marine fishes more broadly. They offer a framework for investigating how convergent ecological pressures interact with genomic features to influence diversification across environmental gradients. More broadly, they highlight the utility of combining high-resolution phylogenomics with phenotype-aware selection models to illuminate the genetic signatures of dietary specialization and adaptive evolution across the Tree of Life.

## Method

### DNA extractions, exon capture, sequencing, and assembly

We generated new exon capture data from tissue samples extracted from museum voucher specimens for a total of 57 acanthuriforms (*Acanthurus tractus* duplicated) and 9 outgroups from closely related families (Supplementary Data 1). High-quality DNA extractions were sent to Arbor Biosciences for library preparation and exon capture to target the 1105 single-copy exons developed for the FishLife project^126^ using the Eupercaria-specific probe set^55^, that also includes PCR-based 29 legacy markers (mtDNA and nuclear genes, see Supplementary Table 1) commonly used for fish phylogenetics^127–129^. Enriched libraries were sequenced using one lane of the Illumina HiSeq 4000 platform with paired-end 100 bp at the University of Chicago Genomics facility. The raw sequence data was assembled and aligned using the bioinformatic pipeline developed by Hughes et al.^55^ and available at https://github.com/lilychughes/FishLifeExonCapture/. The final step of the pipeline generated alignments in their correct reading frames for each exon using MACSE v.2.03^130^. We then removed any instance of single-taxon insertions, trimmed gappy edges, and filtered short sequences using the AlignmentCleanerCodons.py script from the GitHub link above. For species verification, we extracted COI and CYTB sequences for each sample and blasted them against the Barcode of Life Database and National Center for Biotechnology Information (NCBI) repositories using the “bold_identification” python script^131^. We also visually inspected all the alignments to adjust the reading frames, remove poor-quality reads, and correct misaligned sections in Geneious Prime v.2024.0^132^. Alignment summary statistics, such as the percentage of missing data, GC content, proportion of variable sites, and alignment length was assessed using the Python package *AMAS*^133^. After these quality control steps, we removed 9 low-quality markers and 98 markers with more than 50% missing data (present in fewer than 32 species). The final reduced molecular matrix assembled comprised 998 genes for 57 ingroup taxa (56 species out of the 86 extant acanthuriforms), representing all genera and families, along with 9 outgroup species as outlined above.

### Taxonomic sampling augmentation

To increase the number of species, we generated an expanded matrix combining the 66 newly sequenced taxa (reduced matrix, Supplementary Data 1) with 59 species obtained from GenBank (Appendix 1). We individually aligned each of the 16 legacy markers downloaded from GenBank using MACSE and then merged them with the corresponding legacy marker alignments from our reduced matrix (Supplementary Table 1). After manual inspection to ensure correct reading frames, we retained 22 of 29 legacy markers: 20 from our matrix and 9 from GenBank, with 7 shared between both sources (Supplementary Table 1). We combined these 22 markers with the reduced matrix, placing mitochondrial markers at the end to generate a concatenated dataset of 1002 markers for 148 individuals. This total reflects the combination of our focal taxa and all available GenBank sequences, prior to duplicate removal. We subsequently performed an initial quality control assessment of phylogenetic placement using FastTree-2^134^ to identify potential contamination or misidentification. We then removed 58 redundant tips representing duplicate species across datasets. Following phylogenomic inference, *Prionurus punctatus* was renamed *P. laticlavius* based on its recognition as a junior subjective synonym^135^ and the duplicated *P. laticlavius* specimen containing only legacy markers was pruned from the trees. The placement of *Acanthurus tristis* could not be reliably inferred due to incomplete data (COI only, 202 bp) and was therefore excluded from all downstream analyses. The final expanded molecular matrix comprises 1002 genes for 80 ingroup species ( ~ 93%) and 9 outgroup species (65 spp. with FishLife exons and 24 spp. with legacy markers only).

### Phylogenomic inference

For each assembled molecular matrix, reduced and expanded, we inferred maximum likelihood (ML) trees and multispecies coalescent species trees. First, we determined the best-fitting partition scheme for each matrix using PartitionFinder2^136^ based on a priori by-codon partitions for each protein-coding marker, and two partitions for each of the ribosomal markers (12S and 16S). We estimated concatenation-based ML trees in RAxML v.8.2.11^56^ using the best-fit partitioning schemes selected via the Bayesian Information Criterion and the GTRGAMMA model. Using the *raxml-ng* (extension of RAxML for supercomputers^137^), we ran 30 independent ML searches and used 100 nonparametric bootstrap replicates to assess edge support. To infer species trees while accounting for incomplete lineage sorting, we initially estimated individual gene trees in RAxML using by-codon partitions. All mtDNA markers were grouped into a single locus alignment, with by-codon partitions applied specifically for protein-coding genes and two partitions for 12S and 16S, as explained above. After inferring best trees from multiple runs and bootstrap support (BS) values, we collapsed gene tree branches with low BS ( < 33%). We then conducted multispecies coalescent species-tree analyses with multi-locus bootstrapping in ASTRAL-III^57^ using the collapsed gene trees as input to generate a species tree for each matrix. We also assessed gene concordance factors^138^ by calculating the percentage of gene trees in the data matrix that support a specific branch in the concatenation-based (RAxML) and multispecies coalescent-based (ASTRAL-III) species trees inferred for both datasets^138^.

### Integration of fossils and extant species

We newly coded a morphological matrix consisting of 107 characters for 32 fossil and 19 extant acanthuriform species plus 5 extant outgroups (see Supplementary Note 1 for the list of osteological characters and character states). To assess the phylogenetic placement of each species based on morphology, we inferred trees based on parsimony and ML approaches. We estimated the parsimony tree in TNT v.1.5^58^ using a driven-search strategy (sectorial ratchet, tree-fusing methodologies) with default parameters. The ML morphological tree was estimated using the MULTIGAMMA and Mk models with 30 iterations in RAxML. We combined the morphological and the expanded molecular matrices for a total of 112 fossil and extant ingroup species and nine outgroups. We estimated the combined matrix in RAxML using the MULTIGAMMA and Mk models, 100 bootstrap replicates, and six partitions: five for the molecular sequences (one for each codon position of all nuclear and mtDNA protein-coding markers, plus two for 12S and 16S) and one for the morphological dataset. Taxa with polymorphic character states were coded as missing (“?”) for RAxML, which cannot handle polymorphic characters.

### Total-evidence dating analyses and phylogenetic uncertainty

We conducted divergence time estimations under a total-evidence, or tip-dating, framework using the Fossilized Birth Death model in MrBayes v 3.2.7a^61^. To account for topological uncertainty, we assembled largely independent subsets (randomly subsampled from the expanded matrix with genes only), each with enough number of genes to overcome sampling error. Specifically, we partitioned the complete dataset into 20 subsets (50 loci ×13 + 49 loci ×7), 10 subsets (99 loci ×7 + 100 loci ×3), and 5 subsets (199 loci ×3 + 198 loci ×2). To maintain consistent taxon representation across subsets, all shared nine anchor genes. We then ran ML trees using RAxML-NG for each subset, revealing substantial topological discordance, particularly among trees estimated with smaller numbers of loci (Supplementary Fig. 8). Given this pattern, we retained the five largest subsets (198–199 loci each) for downstream total-evidence dating analyses.

Each genomic subset was combined with the morphological matrix (including fossil and extant taxa), yielding a total of 112 taxa per total-evidence analysis. The dataset was divided into five partitions (three codon positions, 12S + 16S, morphology). Molecular partitions were modeled under GTR + G, whereas the morphological partition was analyzed using the MK model. Fossil ages and calibration priors used for divergence time estimation are listed in Supplementary Table 2. To evaluate the effect of root calibration choice, we conducted two null analyses using one representative genomic subset. In the first, we applied a secondary calibration on stem Acanthuriformes (68–75 Ma), with *Chaetodon striatus* used as the outgroup (Supplementary Table 3). In the second, we omitted this root calibration entirely. These analyses yielded crown Acanthuriformes age estimates of 73.8 Ma (with root calibration) and 80.8 Ma (without root calibration), respectively. Because both estimates were older than those reported in recent large-scale phylogenetic studies (refs. ^48,63,139^; see Supplementary Table 3), we instead implemented a secondary calibration on crown Acanthuriformes using a uniform prior of 55.8–64 Ma. Speciation and extinction priors were estimated using the “fit.bd” function in *phytools*^71^, based on a well-sampled acanthuriform tree^140^. That tree was rescaled by adjusting its crown age from 77 Ma to 60 Ma to align with previously published age estimates (Supplementary Table 3).

After independently estimating phylogenies using morphology alone (Supplementary Fig. 6) and using a dataset combining morphology and molecular information (Supplementary Fig. 7), we constrained the three acanthuriform families (Luvaridae, Zanclidae, and Acanthuridae), including both fossil and extant species, to be monophyletic. We implemented this constraint because the full total-evidence dating runs were computationally intensive and expected to require months to complete, and we sought to avoid recovering paraphyletic families despite strong prior evidence supporting their monophyly. We also observed incongruent placements for two fossils, †*Gazolaichthys vestenanovae* and †*Padovathurus gaudryi*. In the combined matrix, both fossils were resolved as sister to Zanclidae + Acanthuridae (Scheme 1, Supplementary Fig. 7), whereas in the morphology-only analysis (Supplementary Fig. 6) and in a previous study^52^ they were placed as sister to Acanthuridae (Scheme 2). Although two constraints were applied for tip-dating analyses, we favor the placement in Scheme 1 over Scheme 2. This interpretation is consistent with Tyler^62^, who noted that osteological differences between the families Acanthuridae and Zanclidae are minimal and may not warrant separation at the family level. From an anatomical standpoint, the distinction between these two families appears to reflect historical taxonomic practice more than strong morphological discontinuity.

To address this topological ambiguity, we implemented both fossil-placement schemes as alternative constraints in the tip-dating analyses. This resulted in 10 MrBayes analyses (two schemes by five genomic subsets). Each analysis was run with eight independent runs and four Monte Carlo Markov chains (MCMCs) for over 350 million generations each, sampling every 10,000 generations. We used a sample probability of 0.94 and a relaxed clock model with the clock rate prior following a log normal distribution and independent gamma rate. The first 10% of trees sampled were discarded as relative burn-in and convergence of the MCMC was verified using the ESS criterion for each parameter in TRACER v.1.7^141^. After more than 14 months of total runtime, we found that 9 of the 10 analyses reached convergence where ESS values were close to or above 200 (Supplementary Table 4). Because these analyses ran for over a year and Subset 2 based on the Scheme 2 had not reached satisfactory ESS values, we removed it from all downstream analyses. We sampled ~2000 trees for Scheme 1 and ~2500 trees for Scheme 2, evenly distributed along the PD post-burn-in from each subset to have a total of 10,000 trees. For each scheme independently, we inferred a Maximum Clade Credibility tree (MCC tree) in TreeAnnotator v.2.7.5^142^. To obtain a PD of trees for phylogenetic comparative analyses, depending on the type of analysis and their computational time, we sampled either 100 or 4 trees from each subset, resulting in a total of 500 and 20 trees for each scheme.

### Diet and biogeographic data

We compiled a discrete diet database for extant and fossil taxa (i.e., non-planktivore vs. planktivore). While extant species were coded based on the diet composition from the literature^12^ and FishBase^67^, fossil diet is typically determined based on their tooth morphology, paleoecology, and paleoenvironment^68,69^. However, for acanthuriforms, this task is particularly daunting given the complex tooth morphology in the group. We thus examined if there is a correlation between tooth morphology and diet for extant species only by codifying three types of teeth: conical, multi-denticulate, and brush-like. We then conducted a phylogenetic regression to determine their relationship, finding no significant evolutionary correlation (*p* = 0.842; Supplementary Fig. 11). We find cases where both non-planktivorous and planktivorous fishes displayed multi-denticulate or conical tooth morphologies. This result indicates that factors other than tooth morphology should be used to determine the diet of acanthuriform fossils. Therefore, we focused on the paleoenvironment of each fossil, categorizing fossils derived from oceanic sediments as planktivores and from limestones as non-planktivores^49,70^. Two fossils (†*Gazolaichthys vestenanovae* and †*Padovathurus gaudryi*) were coded as ambiguous.

We also gathered geographic distribution data and built a presence/absence matrix by coding each extant and fossil species according to their geographic ranges, primarily based on the IUCN Red List^143^, Ocean Biogeographic Information System^144^, and Paleobiology database (https://paleobiodb.org/), as well as from the primary literature. We used a seven-region biogeographic scheme (based on refs. ^145,146^): WIO, CIP, CP, TEP, WA, EA, and Tet.

### Ancestral diet and ancestral range estimates

To examine the frequency of shifts to planktivory and their timing, we conducted ancestral diet reconstruction analyses using “make.simmap” function in the R package *phytools*^71^. We fixed the root state to non-planktivory, considering that close acanthuriform relatives primarily feed on algae and benthic invertebrates, such as Chaetodontidae (butterflyfishes^147^) and Pomacanthidae (angelfishes^78^). We first assessed whether these trophic transitions fit into a model where shifts between non-planktivory (state 0) and planktivory (state 1), and vice versa, exhibit equal rates (ER), or if instead these transitions are different (ARD^71^). We then conducted stochastic character mapping using the best-fit model on the MCC tree and across 20 trees of each scheme. Transitions were counted when a nodal pie is >50% of state 0 and one of its descendant branches is >50% for state 1 and vice versa. Exceptions were made following the parsimony principle when a nodal pie with one state is between two nodes with the alternative state, resulting in fewer transition counts. For comparison purposes only, we also ran the analysis excluding all the fossils for MCC tree from Scheme 1.

We also investigated where trophic transitions occurred. We conducted ancestral range reconstruction analyses in the R package *BioGeoBEARS*^72^ following the approach outlined in ref. ^77^. We evaluated 12 biogeographic models combining DEC^148^, DIVA^149^, and BayAREA^150^, with and without the jump-dispersal or founder-speciation event (j)^151^ and the dispersal matrix power exponential (w) parameters^152^. We analyzed each model using three time slices based on two major geological events: (i) prior to the closure of the Tethys Seaway (65–16 or 12 Ma), (ii) after the closure of the Tethys Seaway and prior to the last rising of the Panama Isthmus (16 or 12–2.8 Ma), and (iii) after the last rising of the Panama Isthmus (2.8–0 Ma^153^). Due to the ambiguous age of the total closure of the Tethys Seaway, the terminal Tethyan event, we ran separate biogeographic analyses assuming 16^73^ and 12 Ma^74–76^ as the final closure. We also accounted for connectivity between areas by implementing three different dispersal probability categories: 1 (high connectivity), 0.5 (intermediate separation), and 0.0001 (wide separation/no connectivity). We ran these analyses for both schemes using the MCC trees with fossils as input phylogenies. We then selected the best-fit biogeographic model based on the Akaike Information Criterion scores corrected for small sample size (Supplementary Table 5). The best-fit model was implemented in 20 time-trees, and the results were summarized by overlying average probabilities across compatible nodes on each MCC tree^154^.

### Diet-dependent diversification

We estimated diversification and transition rates between non-planktivorous and planktivorous diets by conducting SSE analyses in *HiSSE*^82^. Because SSE analyses cannot handle non-ultrametric trees, for these analyses, we used extant-only trees (but see below). We fitted a total of five SSE models with and without unknown (“hidden”) states: null BiSSE model (rates independent of the trait), full BiSSE or BiSSE-like HiSSE (rates dependent on the trait), full HiSSE model (rates dependent on the trait with hidden states), CID-2 model (character-independent model with two hidden states), and CID-4 model (character-independent model with four hidden states). We defined sampling fractions for each trophic guild to account for missing taxa; non-planktivores 91% and planktivores 100%. As with ancestral diet reconstruction analyses, we also fixed the root to non-planktivory. We ran this analysis on the MCC tree and across 20 trees of each scheme. To incorporate uncertainties in model choice, we then model-averaged rates for all tips and nodes in the trees calculated under five models using AIC weights. We then used the R package *ggplot2*^155^ to generate box plots and assess differences in diversification rates between the two states for all tips and nodes. Finally, we statistically compared tip-associated lineage diversification rates between the trophic guilds conducting a phylogenetic ANOVA in *phytools*^71^ with Bonferroni correction.

To incorporate fossil data into our diversification analyses, we estimated the number of lineages over the evolutionary history of this group for the two trophic guilds. We generated LTT plots in *phytools*^71^ using the 500 trees for each scheme. For comparison purposes only, we also ran this analysis excluding all the fossils for MCC tree from Scheme 1 with 100 simulations.

### Paleoclimate-dependent diet evolution

We compiled paleoclimatic temperature curves spanning the last ~64 Ma of acanthuriforms’ evolutionary history to examine the association of planktivory with two temperature datasets: the global average ocean temperatures obtained from oxygen isotope data and the tropical ocean temperatures based on sea surface temperatures from tropical latitudes^85^. We assessed the association of the global and tropical paleoclimatic temperature curves with the evolution of planktivory using a modified time-dependent regression threshold model recently developed by Melendez-Vázquez et al.^87^. We fitted a total of four different evolutionary models for discrete data separately for both temperature curves: three climate-independent models, Brownian motion, early burst, and phylogenetic signal or lambda, and a climate-dependent model. We used 100 integrations (*N* = 100) and a climatic spline interpolation function based on 500 degrees of freedom (df = 500). For each curve, we fitted all these models using both the MCC tree and the 500 trees of each scheme.

### Sequencing and assembly of a chromosome-level genome for *Acanthurus chirurgus*

We generated a phased chromosome-level genome for the Doctorfish tang *A. chirurgus*. We obtained flash-frozen muscle tissues from an individual caught using a hand net in the Florida Keys (24°59.564 N, 80°25.753 W), US, by Phillipp Rauch on the 29th of January of 2023. This specimen was collected under a valid Commercial Saltwater Products License with a Marine Life endorsement (MLD-299) issued by the Florida Fish and Wildlife Conservation Commission. The collection was conducted in accordance with applicable state regulations governing marine life harvest. The voucher specimen is deposited at Scripps Institution of Oceanography (SIO), collection number SIO 24-10. Subsequently, we outsourced the DNA and RNA extractions, library preparations, sequencing, assembly, and annotation to Cantata Bio LLC.

High molecular weight DNA extraction using the Qiagen Blood and Cell Culture DNA Kit following the manufacturer’s protocol. DNA samples were quantified using Qubit 2.0 Fluorometer (Life Technologies, Carlsbad, CA, USA). The PacBio SMRTbell library ( ~ 20 kb) for PacBio Sequel was constructed using SMRTbell Express Template Prep Kit 2.0 (PacBio, Menlo Park, CA, USA) and the manufacturer recommended protocol. The library was bound to polymerase using the Sequel II Binding Kit 2.0 (PacBio) and loaded onto PacBio Sequel II. Sequencing was performed on PacBio Sequel II 8 M SMRT cells. PacBio CCS reads were assembled into scaffolds using Hifiasm v.0.15.4-r347^156^ with default parameters. Blast results of the Hifiasm output assembly against the nt database were used as input for blobtools2 v.1.1.1^157^ and scaffolds identified as possible contamination were removed from the assembly. Finally, purge_dups3 v.1.2.5^158^ was used to remove haplotigs and contig overlaps, yielding one de novo assembly for each haplotype.

To improve genome architecture through scaffolding and read orientation, Dovetail Omni-C library sequencing was performed on an Illumina HiSeqX platform at ~30× sequence coverage^159^. Each Dovetail Omni-C library, chromatin was fixed in place with formaldehyde in the nucleus. Fixed chromatin was digested with and then extracted, chromatin ends were repaired and ligated to a biotinylated bridge adapter, followed by proximity ligation of adapter-containing ends. After proximity ligation, crosslinks were, the DNA purified. Purified DNA was treated to remove biotin that was not internal to ligated fragments. Sequencing libraries were generated using enzymes and Illumina-compatible adapters. Biotin-containing fragments were isolated using streptavidin beads before PCR enrichment of each library.

The de novo assembly and Dovetail reads were then assembled with Omni-C HiRise, a software pipeline designed specifically for using proximity ligation data to scaffold genome assemblies^160^. Dovetail sequences were aligned to the draft input assembly using bwa (https://github.com/lh3/bwa). The separations of Dovetail read pairs mapped within draft scaffolds were analyzed by produce a likelihood model for genomic distance between read pairs, and the model was used to identify and break putative, to score prospective joins, and make joins above a threshold.

Transcriptomic data was obtained by extracting RNA, preparing libraries, and sequencing. Total RNA extraction was done using the QIAGEN RNeasy Plus Kit following manufacturer protocols. Total RNA was quantified using Qubit RNA Assay and TapeStation 4200. Prior to library prep, we performed DNase treatment followed by AMPure bead clean up and QIAGEN FastSelect HMR rRNA depletion. Library preparation was done with the NEBNext Ultra II RNA Library Prep Kit following manufacturer protocols. Then these libraries were run on the Illumina NovaSeq 6000 platform in 2 × 150 bp configuration.

Genome completeness was assessed using Benchmarking Universal Single-Copy Orthologs (BUSCO) v.6.0.0^99,100^ based on the single-copy orthologs for ray-finned fishes database (actinopterygii_odb12). Scaffolds were assigned chromosomal numbers based on their length as no other surgeonfish genome was available at the time of assembly. Contiguity statistics of scaffolded assembly per haplotype was computed using Quast^161^.

For the genome annotation, first repeat families found in the genome assembly of *A. chirurgus* were identified de novo and classified using the software package RepeatModeler v.2.0.1^162^. The custom repeat library obtained from RepeatModeler were used to discover, identify, and mask the repeats in the assembly file using RepeatMasker v.4.1.0^163^. Coding sequences from *Acanthochromis polyacanthus* (GCF_021347895.1), *Chaetodon austriacus* (draft genome^164^), and *Chelmon rostratus* (GCF_017976325.1) were used to train the initial ab initio model for *A. chirurgus* using AUGUSTUS v.2.5.5^165^, with six rounds of optimization. The same coding sequences were also used to train a separate ab initio model for *A. chirurgus* with SNAP (v.2006-07-28; https://github.com/KorfLab/SNAP). RNAseq reads were mapped onto the genome using the STAR v.2.7 aligner software^166^ and intron hints generated with the bam2hints tools in AUGUSTUS. Gene prediction in the repeat-masked genome was performed using MAKER2^167^, SNAP, and AUGUSTUS (with intron-exon boundary hints provided from RNA-Seq). To guide predictions, Swiss-Prot peptide sequences from the UniProt database^168^ were downloaded and used in conjunction with the protein sequences from *A. polyacanthus*, *C. austriacus*, and *C. rostratus* to generate peptide evidence in the MAKER2 pipeline. Only genes that were predicted by both SNAP and AUGUSTUS software were retained in the final gene sets. To help assess the quality of the gene prediction, AED scores were generated for each of the predicted genes as part of the MAKER2 pipeline. Genes were further characterized for their putative function by performing a BLAST search of the peptide sequences against the UniProt database. tRNAs were predicted using the software tRNAscan-SE v.2.05^169^.

### Sequencing and assembly of short-read genomes

Using the libraries prepared at Arbor Biosciences, we sequenced genomes for a total of 48 acanthuriform species (average genome size 0.8 Gbp). We sequenced short reads aiming for 30× coverage using the Illumina NovaSeq 6000 S4 PE150 platform at the Oklahoma Medical Research Foundation sequencing facility. We pre-assembled the raw data in MaSuRCA v.4.0.8^101^ and removed three species due to poor sequencing quality. We then enhanced scaffolding of each genome (Supplementary Data 2) by using as a reference the chromosome-level genome for *A. chirurgus* in RagTag v.2.1.0^102^ to reorder, reorient, and fill gaps. Because *A. chirurgus* was represented by both a reference genome and a short-read genome, the latter was excluded from downstream analyses. Finally, we assessed genome completeness for the 44 species in BUSCO using the same procedure as for chromosome-level genome. This analysis also identified single-copy and duplicated genes within each species based on the ray-finned fishes database.

### Identification of orthologous sequences for positive selection analyses

We first identified single-copy genes among the 23,548 protein-coding genes in the reference genome, along with three reference genomes from closely related species available in NCBI: *Larimichthys crocea* (Sciaenidae), *Epinephelus lanceolatus* (Serranidae), and *Chelmo rostratus* (Chaeotodontidae). We used OrthoFinder^170^ with protein sequences as input (translated with SeqKit v2.8.2^171^), identifying 6381 single-copy orthologs among these four species. We then mined these genes from the 44 short-read data using HMMER v.3.1^172^. All 6381 mined genes were aligned using MACSE^130^, followed by cleaning and filtering steps as described for the exon capture data. Because positive selection analyses are highly sensitive to alignment errors^173^, we performed exhaustive quality control. Specifically, we trimmed alignments in MACSE, allowing a maximum of 20% gaps per site, and excluded genes containing fewer than 70% of species ( ≤ 31 taxa) or shorter than 200 bp. Alignments were visually inspected in Geneious Prime v.2024.0^132^, to adjust reading frames, remove poor-quality reads, and correct misalignments. After filtering, we translated the remaining 4940 gene alignments into protein sequences and ran them in OrthoFinder to confirm that they still represented one-to-one orthologs. Using the pruned MCC tree from Scheme 1 to match the species with available genome data, we ultimately retained a set of 2542 single-copy orthologous genes for positive selection analyses. In addition to these mined genes, we also identified single-copy orthologs from the BUSCO Actinopterygii database (odb10) and AUGUSTUS as gene predictor. OrthoFinder identified 1269 BUSCO orthologs, of which 1250 passed quality control filters explained above. Following the initial round of peer review, two referees recommended updating our ortholog search to incorporate the most recent Actinopterygii BUSCO database (odb12). We therefore re-ran the pipeline using this updated version and recovered 1666 single-copy orthologs. To evaluate their correspondence with the previously compiled gene set (derived from reference genome and BUSCO odb10, see Supplementary Data 3), we compared not only gene identifiers, which differ between database versions, but also sequence similarity using Geneious Prime. Of the 1666 orthologs, 1532 were duplicated from our earlier set. We added the remaining 134 newly recovered loci to the BUSCO dataset, resulting in a total of 1384 loci for positive selection analyses.

### Analyses of positive selection associated with trophic shifts

To identify instances of positive selection associated with planktivory, we apply alternative approaches implemented in HyPhy^103^ that use the dN/dS metric (the ratio of non-synonymous substitutions [dN] to synonymous substitutions [dS]). We interrogated a total of 3926 single-copy genes, including 2542 mined from the reference genome’s protein-coding genes and 1384 from the BUSCO databases. The MCC tree from Scheme 1 was pruned to retain only the species present in each gene alignment. We focused on convergently evolved PSGs by testing for positive selection exclusively along branches corresponding to transitions to planktivory, as inferred from ancestral diet reconstruction analyses (Fig. 3A). We first applied the adaptive Branch-Site Random Effects Likelihood method (aBSREL^104^). Although a full data-driven run across all branches is standard, it is computationally intensive. Thus, we adopted a hypothesis-driven approach by testing only the five foreground branches corresponding to dietary shifts (see Fig. 3A), greatly reducing computational burden. This approach allowed detection of genes under positive selection associated with the transition to planktivory (Scenario 1), selection acting after the transition (Scenario 2), and convergence across planktivore lineages.

aBSREL is restricted to focal branches and cannot detect positive selection in non-focal branches; we therefore complemented these analyses with BUSTED-PH. BUSTED-PH distinguishes positive selection between foreground and background lineages by explicitly modeling phenotype-associated selection pressures. In this analysis, stem and crown planktivore lineages were designated as foreground branches, non-planktivores as background, and reversals to a non-planktivore diet as nuisance branches, therefore excluded from inference (Fig. 4B and Supplementary Fig. 23). After correcting for multiple testing (FDR *p* < 0.05) in both aBSREL and BUSTED-PH, we initially identified 1461 PSGs (382 from the genome dataset and 1079 from BUSCO). Of these, 993 genes (68 genome-derived and 925 BUSCO-derived) also showed evidence of selection in background branches and were removed. Visual inspection in Geneious Prime of the remaining 467 genes (314 genome + 154 BUSCO) confirmed quality for 160 genes without changes, while 307 required manual editing (e.g., trimming gappy regions). One gene was excluded due to a possible instance of out-paralogy, and *Ctenochaetus striatus* was removed from all alignments due to sequencing error. We then reran aBSREL and BUSTED-PH on the remaining 466 genes, repeating the same filtering pipeline. We examined the overlap between both approaches and removed six duplicated genes from the dataset. This resulted in a final curated set of 302 PSGs (197 genome + 105 BUSCO). Of these, 39 PSGs (15 genome + 24 BUSCO) were identified in at least two of the five planktivory transitions. We visualized shared convergence across lineages using an UpSet plot generated with *UpSetR*^174^. GO terms and functional categories were retrieved for each PSG using the PANTHER Classification System (https://www.pantherdb.org^106^) and the ZFIN database^107^ to determine gene function and expression profiles.

We further tested whether the 39 convergently evolved PSGs exhibited accelerated or decelerated rates of evolution in planktivore lineages using the RER method in HyPhy, implemented via the RERconverge R package^175^. This approach identifies foreground branches with significantly different evolutionary rates relative to the background. As input, we used amino acid alignments for each gene and an unconstrained phylogram generated from all exons using RAxML. The unconstrained phylogram was pruned to match the species present in each gene alignment. To investigate site-specific signals of episodic diversifying selection, we applied MEME^113^ in HyPhy to the subset of genes showing accelerated rates. This analysis also allowed us to assess whether significant codons (*p* < 0.1) exhibited patterns of convergence, either via different amino acid changes at the same site or changes at different sites, or parallelism, defined as identical amino acid substitutions at the same site across the five planktivory transitions. For MEME, we used the same amino acid alignments and the RAxML-based phylogram used in the RER analysis.

### Variations in transposable elements linked to trophic shifts

First, we used Tandem Repeats Finder for the identification of tandem repeats^176^. We then conducted both homology-based and de novo methods to detect TEs. The homology-based TE identification for all species was carried out using RepeatMasker and based on the Repbase library^177^. Additionally, the de novo TE annotation was conducted using RepeatModeler with default settings to create a de novo repeat library for each assembled genome. Subsequently, we employed the de novo repeat library in conjunction with RepeatMasker to predict soft-masked repeats for each species. The identified repeat elements were categorized into ten distinct class/subfamilies, encompassing long interspersed nuclear elements, short interspersed nuclear elements, long terminal repeats, transposons, rolling circle elements, small RNAs, satellites, simple repeats, low complexity repeats, and unclassified elements. To evaluate the correlation between the quantity of TEs (total and for each class/subfamily) and trophic guilds, we conducted a phylogenetic ANOVA in *phytools*^71^ using Bonferroni correction.

### Reporting summary

Further information on research design is available in the Nature Portfolio Reporting Summary linked to this article.

## Supplementary information

Supplementary information

Descriptions of Additional Supplementary Files

Supplementary Data 1

Supplementary Data 2

Supplementary Data 3

Supplementary Data 4

Supplementary Data 5

Reporting Summary

Transparent Peer Review file

## Source data

Source Data

## Data Availability

The genomic data generated in this study have been deposited in the NCBI database under the umbrella BioProject PRJNA1433022. Raw sequence reads for exon-capture and short-reads are under the BioProject PRJNA1418135. Accession numbers for exon-capture data and short-read genomes are provided in Supplementary Data 1 and 2, respectively. Museum collection numbers and the collected geographic locations for all sampled specimens can also be found in Supplementary Data 1 and 2. The voucher specimen of *Acanthurus chirurgus* used to generate the chromosome-level genome in this study is deposited at Scripps Institution of Oceanography (SIO), under collection number SIO 24-10. PacBio and Hi-C raw reads for the *A. chirurgus* genome are also available under BioProject PRJNA1418135. The phased haplotypes assemblies are deposited under BioProjects PRJNA1430673 (principal haplotype, accession number JBVQTB000000000) and PRJNA1430672 (alternate haplotype, accession number JBVQTC000000000). All data generated and analyzed during this study are available on Figshare (10.6084/m9.figshare.29594255)^178^, including exon and single-copy ortholog alignments, phylogenetic trees, input files for comparative analyses, and chromosome-level genome annotation files. Source data for the main figures are provided as a Source Data file and on Figshare. Source data are provided with this paper.

## Code availability

All scripts used in this study are available on Figshare (10.6084/m9.figshare.29594255)^178^.

## References

[1] Losos, J. B. Convergence, adaptation, and constraint. *Evolution* **65**, 1827–1840 (2011). 21729041 10.1111/j.1558-5646.2011.01289.x

[2] Cooper, W. J. et al. Bentho-pelagic divergence of cichlid feeding architecture was prodigious and consistent during multiple adaptive radiations within African Rift-Lakes. *PLoS ONE* **5**, e9551 (2010). 20221400 10.1371/journal.pone.0009551 PMC2833203

[3] Burress, E. D., Holcomb, J. M., Tan, M. & Armbruster, J. W. Ecological diversification associated with the benthic-to-pelagic transition by North American minnows. *J. Evolut. Biol.* **30**, 549–560 (2017). 10.1111/jeb.13024 27925684

[4] Friedman, S. T. et al. Body shape diversification along the benthic–pelagic axis in marine fishes. *Proc. R. Soc. B: Biol. Sci.* **287**, 20201053 (2020). 10.1098/rspb.2020.1053 PMC7423681 32693721

[5] Præbel, K. et al. Ecological speciation in postglacial European whitefish: rapid adaptive radiations into the littoral, pelagic, and profundal lake habitats. *Ecol. Evol.* **3**, 4970–4986 (2013). 24455129 10.1002/ece3.867 PMC3892361

[6] Hulsey, C. D., Roberts, R. J., Loh, Y. H. E., Rupp, M. F. & Streelman, J. T. Lake Malawi cichlid evolution along a benthic/limnetic axis. *Ecol. Evol.* **3**, 2262–2272 (2013). 23919168 10.1002/ece3.633 PMC3728963

[7] Elmer, K. R. et al. Parallel evolution of Nicaraguan crater lake cichlid fishes via non-parallel routes. *Nat. Commun.* **5**, 1–8 (2014). 10.1038/ncomms6168 25346277

[8] Rundle, H. D., Nagel, L., Boughman, J. W. & Schluter, D. Natural selection and parallel speciation in sympatric sticklebacks. *Science* **287**, 306–308 (2000). 10634785 10.1126/science.287.5451.306

[9] Rincon-Sandoval, M. et al. Evolutionary determinism and convergence associated with water-column transitions in marine fishes. *Proc. Natl. Acad. Sci. USA* **177**, 33396–33403 (2020). 10.1073/pnas.2006511117 PMC7777220 33328271

[10] Tavera, J., Acero, P. A. & Wainwright, P. C. Multilocus phylogeny, divergence times, and a major role for the benthic-to-pelagic axis in the diversification of grunts (Haemulidae). *Mol. Phylogenet. Evol.* **121**, 212–223 (2018). 29307507 10.1016/j.ympev.2017.12.032

[11] Friedman, S. T., Price, S. A., Hoey, A. S. & Wainwright, P. C. Ecomorphological convergence in planktivorous surgeonfishes. *J. Evol. Biol.* **29**, 965–978 (2016). 26809907 10.1111/jeb.12837

[12] Tebbett, S. B., Siqueira, A. C. & Bellwood, D. R. The functional roles of surgeonfishes on coral reefs: past, present and future. *Rev. Fish. Biol. Fish.* **6**, 387–439 (2022).

[13] Lungstrom, L. L., Farjo, M., Isdonas, R., George, A. B. & Westneat, M. W. Phylogenetics, Trait Covariance Analysis, and the Evolution of Fin and Body Shape in the Surgeonfishes. *Evolution* qpag088 10.1093/evolut/qpag088 (2025). 10.1093/evolut/qpag088 42104957

[14] McCord, C. L., Nash, C. M., Cooper, W. J. & Westneat, M. W. Phylogeny of the damselfishes (Pomacentridae) and patterns of asymmetrical diversification in body size and feeding ecology. *PLoS ONE* **16**, e0258889 (2021). 34705840 10.1371/journal.pone.0258889 PMC8550381

[15] Hodge, J. R. et al. Unravelling the effects of ecology and evolutionary history in the phenotypic convergence of fishes. *Syst. Biol.* **74**, 881–896 (2025). 40359153 10.1093/sysbio/syaf034

[16] Ng, I., Bellwood, D. R., Strugnell, J. M., Floeter, S. R. & Siqueira, A. C. More than one way to be a planktivore: the vast morphospace of plankton-feeding reef fishes. *Rev. Fish. Biol. Fish.* **35**, 911–928 (2025).

[17] Siqueira, A. C., Morais, R. A., Bellwood, D. R. & Cowman, P. F. Trophic innovations fuel reef fish diversification. *Nat. Commun.* **11**, 1–11 (2020). 32472063 10.1038/s41467-020-16498-w PMC7260216

[18] Hobson, E. S. Trophic relationships of fishes specialized to feed on zooplankters above coral reefs. In *The Ecology of Fishes on Coral Reefs* (ed. Sale, P. F.) 69–95 (Academic Press, San Diego, 1991).

[19] Duarte-Ribeiro, E., Davis, A. M., Rivero-Vega, R. A., Ortí, G. & Betancur, R. Post-Cretaceous bursts of evolution along the benthic-pelagic axis in marine fishes. *Proc. R. Soc. B: Biol. Sci.* **285**, 20182010 (2018). 10.1098/rspb.2018.2010 PMC6304066 30963906

[20] Gray, J. S. Marine biodiversity: patterns, threats and conservation needs. *Biodivers. Conserv.* **6**, 153–175 (1997).

[21] Colles, A., Liow, L. H. & Prinzing, A. Are specialists at risk under environmental change? Neoecological, paleoecological and phylogenetic approaches. *Ecol. Lett.* **12**, 849–863 (2009). 19580588 10.1111/j.1461-0248.2009.01336.x PMC2730552

[22] Van Valkenburgh, B., Want, X. & Damuth, J. Cope’s Rule, hypercarnivory, and extinction in North American canids. *Science* **306**, 101–104 (2010). 10.1126/science.1102417 15459388

[23] Gajdzik, L., Aguilar-Medrano, R. & Frédérich, B. Diversification and functional evolution of reef fish feeding guilds. *Ecol. Lett.* **22**, 572–582 (2019). 30648337 10.1111/ele.13219

[24] Reddin, C. J., Kocsis, ÁT. & Kiessling, W. Climate change and the latitudinal selectivity of ancient marine extinctions. *Paleobiology* **45**, 70–84 (2019).

[25] Harnik, P. G. et al. Extinctions in ancient and modern seas. *Trends Ecol. Evol.* **27**, 608–617 (2012). 22889500 10.1016/j.tree.2012.07.010

[26] Arcila, D. & Tyler, J. C. Mass extinction in tetraodontiform fishes linked to the Palaeocene-Eocene thermal maximum. *Proc. R. Soc. B: Biol. Sci.* **284**, 20171771 (2017). 10.1098/rspb.2017.1771 PMC5698648 29118135

[27] Davis, K. E., Hill, J., Astrop, T. I. & Wills, M. A. Global cooling as a driver of diversification in a major marine clade. *Nat. Commun.* **7**, 1–8 (2016). 10.1038/ncomms13003 PMC5059450 27701377

[28] De-Kayne, R. et al. Genomic architecture of adaptive radiation and hybridization in Alpine whitefish. *Nat. Commun.* **13**, 1–13 (2022). 35918341 10.1038/s41467-022-32181-8 PMC9345977

[29] Herrera, M. et al. From genes to pathways: a curated gene approach to accurate pathway reconstruction in teleost fish transcriptomics. *J. Exp. Zool. Part B: Mol. Dev. Evol.* **344**, 1–15 (2025). 10.1002/jez.b.23299 40296566

[30] Ahi, E. P., Richter, F. & Sefc, K. M. Gene expression patterns associated with caudal fin shape in the cichlid *Lamprologus tigripictilis*. *Hydrobiologia* **850**, 2257–2273 (2023). 37325486 10.1007/s10750-022-05068-4 PMC10261199

[31] Vizueta, J., Macías-Hernández, N., Arnedo, M. A., Rozas, J. & Sánchez-Gracia, A. Chance and predictability in evolution: the genomic basis of convergent dietary specializations in an adaptive radiation. *Mol. Ecol.* **28**, 4028–4045 (2019). 31359512 10.1111/mec.15199

[32] Chen, H. I., Turakhia, Y., Bejerano, G. & Kingsley, D. M. Whole-genome comparisons identify repeated regulatory changes underlying convergent appendage evolution in diverse fish lineages. *Mol. Biol. Evol.* **40**, 1–18 (2023). 10.1093/molbev/msad188 PMC10516590 37739926

[33] Parker, J. et al. Genome-wide signatures of convergent evolution in echolocating mammals. *Nature* **502**, 228–231 (2013). 24005325 10.1038/nature12511 PMC3836225

[34] Eastment, R. V., Wong, B. B. M. & McGee, M. D. Convergent genomic signatures associated with vertebrate viviparity. *BMC Biol.* **22**, 1–15 (2024). 38331819 10.1186/s12915-024-01837-w PMC10854053

[35] Musilova, Z. et al. Vision using multiple distinct rod opsins in deep-sea fishes. *Science* **364**, 588–592 (2019). 31073066 10.1126/science.aav4632 PMC6628886

[36] Aristide, L. & Fernández, R. Genomic insights into mollusk terrestrialization: parallel and convergent gene family expansions as key facilitators in out-of-the-sea transitions. *Genome Biol. Evol.* **15**, 1–17 (2023). 10.1093/gbe/evad176 PMC10581543 37793176

[37] Singh, P. et al. Phylogenomics of trophically diverse cichlids disentangles processes driving adaptive radiation and repeated trophic transitions. *Ecol. Evol.* **12**, 1–15 (2022). 10.1002/ece3.9077 PMC9288888 35866021

[38] Roycroft, E. et al. Molecular evolution of ecological specialisation: genomic insights from the diversification of murine rodents. *Genome Biol. Evol.* **13**, 1–16 (2021). 10.1093/gbe/evab103 PMC8258016 33988699

[39] Seppey, M. et al. Genomic signatures accompanying the dietary shift to phytophagy in polyphagan beetles. *Genome Biol.* **20**, 1–14 (2019). 31101123 10.1186/s13059-019-1704-5 PMC6525341

[40] Li, D. & Zhang, J. Diet shapes the evolution of the vertebrate bitter taste receptor gene repertoire. *Mol. Biol. Evol.* **31**, 303–309 (2014). 24202612 10.1093/molbev/mst219 PMC3907052

[41] Hecker, N., Sharma, V. & Hiller, M. Convergent gene losses illuminate metabolic and physiological changes in herbivores and carnivores. *Proc. Natl. Acad. Sci. USA* **116**, 3036–3041 (2019). 30718421 10.1073/pnas.1818504116 PMC6386725

[42] Ahi, E. P., Brunel, M., Tsakoumis, E., Chen, J. & Schmitz, M. Appetite regulating genes in zebrafish gut; a gene expression study. *PLoS ONE* **17**, 1–25 (2022). 10.1371/journal.pone.0255201 PMC9295983 35853004

[43] DeLorenzo, L. et al. Genetic basis of ecologically relevant body shape variation among four genera of cichlid fishes. *Mol. Ecol.* **32**, 3975–3988 (2023). 37161914 10.1111/mec.16977 PMC10502943

[44] Glazer, A. M., Cleves, P. A., Erickson, P. A., Lam, A. Y. & Miller, C. T. Parallel developmental genetic features underlie stickleback gill raker evolution. *Evodevo* **5**, 1–16 (2014). 24851181 10.1186/2041-9139-5-19 PMC4029907

[45] Rincon-Sandoval, M. et al. Ecological diversification of sea catfishes is accompanied by genome-wide signatures of positive selection. *Nat. Commun.* **15**, 1–15 (2024). 39567489 10.1038/s41467-024-54184-3 PMC11579386

[46] Shapiro, M. D. et al. The genetic architecture of skeletal convergence and sex determination in ninespine sticklebacks. *Curr. Biol.* **19**, 1140–1145 (2009). 19500990 10.1016/j.cub.2009.05.029 PMC2735127

[47] Tea, Y.-K. et al. The spotted parrotfish genome provides insights into the evolution of a coral reef dietary specialist (Teleostei:Labridae:Scarini:*Cetoscarus ocellatus*). *Ecol. Evol.* **14**, 1–16 (2024). 10.1002/ece3.11148 PMC10932694 38476702

[48] Betancur-R, R. et al. Phylogenetic classification of bony fishes. *BMC Evolut. Biol.* **17**, 1–40 (2017). 10.1186/s12862-017-0958-3 PMC5501477 28683774

[49] Friedman, M. & Carnevale, G. The Bolca Lagerstätten: shallow marine life in the Eocene. *J. Geol. Soc.* **175**, 569–579 (2018).

[50] Bellwood, D. R., Goatley, C. H. R., Brandl, S. J. & Bellwood, O. Fifty million years of herbivory on coral reefs: fossils, fish and functional innovations. *Proc. R. Soc. B: Biol. Sci.* **281**, 20133046 (2014). 10.1098/rspb.2013.3046 PMC3953842 24573852

[51] Klanten, S. O., Van Herwerden, L., Choat, J. H. & Blair, D. Patterns of lineage diversification in the genus *Naso* (Acanthuridae). *Mol. Phylogenet. Evol.* **32**, 221–235 (2004). 15186809 10.1016/j.ympev.2003.11.008

[52] Siqueira, A. C., Bellwood, D. R. & Cowman, P. F. Historical biogeography of herbivorous coral reef fishes: the formation of an Atlantic fauna. *J. Biogeogr.* **46**, 1611–1624 (2019).

[53] Flügel, E. & Kiessling, W. Patterns of Phanerozoic reef crises. *Soc. Sediment. Geol.* **72**, 691–733 (2002).

[54] Smith, S. D., Pennell, M. W., Dunn, C. W. & Edwards, S. V. Phylogenetics is the new genetics (for most of biodiversity). *Trends Ecol. Evol.* **35**, 415–425 (2020). 32294423 10.1016/j.tree.2020.01.005

[55] Hughes, L. C. et al. Exon probe sets and bioinformatics pipelines for all levels of fish phylogenomics. *Mol. Ecol. Resour.* **21**, 816–833 (2020). 33084200 10.1111/1755-0998.13287

[56] Stamatakis, A. RAxML version 8: a tool for phylogenetic analysis and post-analysis of large phylogenies. *Bioinformatics* **30**, 1312–1313 (2014). 24451623 10.1093/bioinformatics/btu033 PMC3998144

[57] Zhang, C., Sayyari, E. & Mirarab, S. ASTRAL-III: increased scalability and impacts of contracting low support branches. *Lect. Notes Comput. Sci.* **10562 LNBI**, 53–75 (2017).

[58] Goloboff, P. A. & Catalano, S. A. TNT version 1.5, including a full implementation of phylogenetic morphometrics. *Cladistics* **32**, 221–238 (2016). 34727670 10.1111/cla.12160

[59] Clements, K. D., Gray, R. D. & Howard Choat, J. Rapid evolutionary divergences in reef fishes of the family Acanthuridae (Perciformes: Teleostei). *Mol. Phylogenet. Evol.* **26**, 190–201 (2003). 12565030 10.1016/s1055-7903(02)00325-1

[60] Sorenson, L., Santini, F., Carnevale, G. & Alfaro, M. E. A multi-locus timetree of surgeonfishes (Acanthuridae, Percomorpha), with revised family taxonomy. *Mol. Phylogenet. Evol.* **68**, 150–160 (2013). 23542000 10.1016/j.ympev.2013.03.014

[61] Ronquist, F. et al. Mrbayes 3.2: efficient Bayesian phylogenetic inference and model choice across a large model space. *Syst. Biol.* **61**, 539–542 (2012). 22357727 10.1093/sysbio/sys029 PMC3329765

[62] Tyler, J. C. Osteological aspects of interrelationships of surgeon fish genera (Acanthuridae). *Proc. Acad. Nat. Sci. Phila.* **122**, 87–124 (1970).

[63] Ghezelayagh, A. et al. Prolonged morphological expansion of spiny-rayed fishes following the end-Cretaceous. *Nat. Ecol. Evol.* **6**, 1211–1220 (2022). 35835827 10.1038/s41559-022-01801-3

[64] Winterbottom, R. Myological evidence for the phylogeny of recent genera of surgeonfishes (Percomorpha, Acanthuridae), with comments on the acanthuroidei. *Copeia* **1993**, 21–39 (1993).

[65] Cantalice, K. M., Alvarado-Ortega, J., Bellwood, D. R. & Siqueira, A. C. Rising from the Ashes: the biogeographic origins of modern coral reef fishes. *Bioscience* **72**, 769–777 (2022). 35923187 10.1093/biosci/biac045 PMC9343231

[66] Walker, J. A. Ecological morphology of lacustrine threespine stickleback *Gasterosteus aculeatus* L. (Gasterosteidae) body shape. *Biol. J. Linn. Soc.* **61**, 3–50 (1997).

[67] Froese, R. & Pauly, D. FishBase. *World Wide Web Electronic Publication*. www.fishbase.org (2025).

[68] DeSantis, L. R. G. Dental microwear textures: reconstructing diets of fossil mammals. *Surf. Topography: Metrol. Prop.* **4**, 023002 (2016).

[69] Purnell, M., Seehausen, O. & Galis, F. Quantitative three-dimensional microtextural analyses of tooth wear as a tool for dietary discrimination in fishes. *J. R. Soc. Interface* **9**, 2225–2233 (2012). 22491979 10.1098/rsif.2012.0140 PMC3405762

[70] Marramà, G., Garbelli, C. & Carnevale, G. A clade-level morphospace for the Eocene fishes of Bolca: patterns and relationships with modern tropical shallow marine assemblages. *Boll. Della Soc. Paleontol. Ital.* **55**, 139–156 (2016).

[71] Revell, L. J. phytools 2.0: an updated R ecosystem for phylogenetic comparative methods (and other things). *PeerJ* **12**, 16505 (2024). 10.7717/peerj.16505 PMC10773453 38192598

[72] Matzke, N. J. *BioGeoBEARS: BioGeography with Bayesian (and Likelihood) Evolutionary Analysis in R Scripts* (R Package, Version 0.2, 2013).

[73] Adams, C. G., Gentry, A. W. & Whybrow, P. J. Dating the terminal Tethyan event. *Utrecht Micropaleontol. Bull.* **30**, 273–298 (1983).

[74] Steininger, F. & Rögl, F. The Paratethys history. A contribution towards the Neogene geodynamics of the Alpine orogeny. *Ann. Géol. Pays Hell.* **3**, 1153–1165 (1979).

[75] Rogl, F. Palaeogeographic considerations for Mediterranean and Paratethys seaways. *Ann. Naturhist. Mus. Wien.* **99A**, 279–310 (1999).

[76] Steininger, F. F. & Rögl, F. Paleogeography and palinspastic reconstruction of the Neogene of the Mediterranean and Paratethys. *Geol. Soc., Lond., Spec. Publ.* **17**, 659–668 (1984).

[77] Santaquiteria, A. et al. Phylogenomics and historical biogeography of seahorses, dragonets, goatfishes, and allies (Teleostei: Syngnatharia): assessing factors driving uncertainty in biogeographic inferences. *Syst. Biol.* **70**, 1145–1162 (2021). 33892493 10.1093/sysbio/syab028

[78] Baraf, L. M., Pratchett, M. S. & Cowman, P. F. Ancestral biogeography and ecology of marine angelfishes (F: Pomacanthidae). *Mol. Phylogenet. Evol.* **140**, 106596 (2019). 31421243 10.1016/j.ympev.2019.106596

[79] Siqueira, A. C., Morais, R. A., Bellwood, D. R. & Cowman, P. F. Planktivores as trophic drivers of global coral reef fish diversity patterns. *Proc. Natl. Acad. Sci. USA* **118**, 1–8 (2021). 10.1073/pnas.2019404118 PMC7936278 33593939

[80] Johansen, J. L., Bellwood, D. R. & Fulton, C. J. Coral reef fishes exploit flow refuges in high-flow habitats. *Mar. Ecol. Prog. Ser.* **360**, 219–226 (2008).

[81] Cope, E. D. *The Primary Factors of Organic Evolution* (Chicago Open Court Pub. Co, 1904).

[82] Beaulieu, J. M. & O’Meara, B. C. Detecting hidden diversification shifts in models of trait-dependent speciation and extinction. *Syst. Biol.* **65**, 583–601 (2016). 27016728 10.1093/sysbio/syw022

[83] Choat, J. H., Clements, K. D. & Robbins, W. D. The trophic status of herbivorous fishes on coral reefs 1: dietary analyses. *Mar. Biol.* **140**, 613–623 (2002).

[84] Clements, K. D. *Gut Microorganisms of Surgeonfishes (Family Acanthuridae)*. PhD thesis http://researchonline.jcu.edu.au/33764/ (James Cook University, Townsville, 1991).

[85] Scotese, C. R., Song, H., Mills, B. J. W. & van der Meer, D. G. Phanerozoic paleotemperatures: the earth’s changing climate during the last 540 million years. *Earth-Sci. Rev.* **215**, 103503 (2021).

[86] Huber, M. & Caballero, R. The early Eocene equable climate problem revisited. *Climate* **7**, 603–633 (2011).

[87] Melendez-Vazquez, F. et al. Ecological interactions and genomic innovation fueled the evolution of ray-finned fish endothermy. *Sci. Adv.* **11**, eads8488 (2025). 40561012 10.1126/sciadv.ads8488 PMC12190012

[88] McInerney, F. A. & Wing, S. L. The paleocene-eocene thermal maximum: a perturbation of carbon cycle, climate, and biosphere with implications for the future. *Annu. Rev. Earth Planet. Sci.* **39**, 489–516 (2011).

[89] Zachos, J. C., Dickens, G. R. & Zeebe, R. E. An early Cenozoic perspective on greenhouse warming and carbon-cycle dynamics. *Nature* **451**, 279–283 (2008). 18202643 10.1038/nature06588

[90] Bellwood, D. R. The Eocene fishes of Monte Bolca: the earliest coral reef fish assemblage. *Coral Reefs* **15**, 11–19 (1996).

[91] Morard, R. et al. Renewal of planktonic foraminifera diversity after the Cretaceous Paleogene mass extinction by benthic colonizers. *Nat. Commun.* **13**, 1–9 (2022). 36414628 10.1038/s41467-022-34794-5 PMC9681854

[92] Kiessling, W. & Simpson, C. On the potential for ocean acidification to be a general cause of ancient reef crises. *Glob. Change Biol.* **17**, 56–67 (2011).

[93] Lowery, C. M., Bown, P. R., Fraass, A. J. & Hull, P. M. Ecological response of plankton to environmental change: thresholds for extinction. *Annu. Rev. Earth Planet. Sci.* **48**, 403–429 (2020).

[94] Filippi, G. et al. Impacts of the Early Eocene Climatic Optimum (EECO, ∼53-49 Ma) on planktic foraminiferal resilience. *Paleoceanogr. Paleoclimatol.* **39**, e2023PA004820 (2024).

[95] Bellwood, D. R., Goatley, C. H. R. & Bellwood, O. The evolution of fishes and corals on reefs: form, function and interdependence. *Biol. Rev.* **92**, 878–901 (2017). 26970292 10.1111/brv.12259

[96] Siqueira, A. C., Muruga, P. & Bellwood, D. R. On the evolution of fish–coral interactions. *Ecol. Lett.* **26**, 1348–1358 (2023). 37222494 10.1111/ele.14245

[97] Steinthorsdottir, M. et al. The Miocene: the future of the past. *Paleoceanogr. Paleoclimatol.* **36**, e2020PA004037 (2021).

[98] Foo, S. A., Teague, C. H. & Asner, G. P. Warming alters the relationship between benthic cover and herbivores on Hawaiian reefs. *Front. Mar. Sci.* **9**, 1–12 (2022).35450130

[99] Simão, F. A., Waterhouse, R. M., Ioannidis, P., Kriventseva, E. V. & Zdobnov, E. M. BUSCO: assessing genome assembly and annotation completeness with single-copy orthologs. *Bioinformatics* **31**, 3210–3212 (2015). 26059717 10.1093/bioinformatics/btv351

[100] Tegenfeldt, F. et al. OrthoDB and BUSCO update: annotation of orthologs with wider sampling of genomes. *Nucleic Acids Res.* **53**, D516–D522 (2025). 39535043 10.1093/nar/gkae987 PMC11701741

[101] Zimin, A. V. et al. The MaSuRCA genome assembler. *Bioinformatics* **29**, 2669–2677 (2013). 23990416 10.1093/bioinformatics/btt476 PMC3799473

[102] Alonge, M. et al. Automated assembly scaffolding using RagTag elevates a new tomato system for high-throughput genome editing. *Genome Biol.* **23**, 1–19 (2022). 36522651 10.1186/s13059-022-02823-7 PMC9753292

[103] Kosakovsky Pond, S. L. et al. HyPhy 2.5 - A customizable platform for evolutionary hypothesis testing using phylogenies. *Mol. Biol. Evol.* **37**, 295–299 (2020). 31504749 10.1093/molbev/msz197 PMC8204705

[104] Smith, M. D. et al. Less is more: an adaptive branch-site random effects model for efficient detection of episodic diversifying selection. *Mol. Biol. Evol.* **32**, 1342–1353 (2015). 25697341 10.1093/molbev/msv022 PMC4408413

[105] Selberg, A. et al. BUSTED-PH: isolating the genomic signatures of convergent phenotypes. Preprint at https://www.biorxiv.org/content/10.64898/2026.01.29.702612v1 (2026).

[106] Thomas, P. D. et al. PANTHER: making genome-scale phylogenetics accessible to all. *Protein Sci.* **31**, 8–22 (2022). 34717010 10.1002/pro.4218 PMC8740835

[107] Bradford, Y. et al. ZFIN: enhancements and updates to the zebrafish model organism database. *Nucleic Acids Res.* **39**, D822–D829 (2011). 21036866 10.1093/nar/gkq1077 PMC3013679

[108] Volkoff, H. The neuroendocrine regulation of food intake in fish: a review of current knowledge. *Front. Neurosci.* **10**, 540 (2016). 27965528 10.3389/fnins.2016.00540 PMC5126056

[109] Capell-Hattam, I. M., Fenton, N. M., Coates, H. W., Sharpe, L. J. & Brown, A. J. The non catalytic protein ERG28 has a functional role in cholesterol synthesis and is coregulated transcriptionally. *J. Lipid Res.* **63**, 100295 (2022). 36216146 10.1016/j.jlr.2022.100295 PMC9730225

[110] von Hofsten, J. & Olsson, P. E. Zebrafish sex determination and differentiation: involvement of FTZ-F1 genes. *Reprod. Biol. Endocrinol.* **3**, 1–11 (2005). 16281973 10.1186/1477-7827-3-63 PMC1298332

[111] Gesemann, M., Lesslauer, A., Maurer, C. M., Schönthaler, H. B. & Neuhauss, S. C. Phylogenetic analysis of the vertebrate excitatory/neutral amino acid transporter (SLC1/EAAT) family reveals lineage specific subfamilies. *BMC Evol. Biol.* **10**, 1–15 (2010). 20429920 10.1186/1471-2148-10-117 PMC2873418

[112] Barat, A. et al. Data on solute carrier transporter genes of a threatened Himalayan fish species – Schizothorax richardsonii. *Data Brief.* **23**, 103712 (2019). 31372384 10.1016/j.dib.2019.103712 PMC6660431

[113] Murrell, B. et al. Detecting individual sites subject to episodic diversifying selection. *PLoS Genet.* **8**, 1–10 (2012). 10.1371/journal.pgen.1002764 PMC3395634 22807683

[114] Sackton, T. B. & Clark, N. Convergent evolution in the genomics era: new insights and directions. *Philos. Trans. R. Soc. B: Biol. Sci.* **374**, 20190102 (2019). 10.1098/rstb.2019.0102 PMC6560275 31154976

[115] Yuan, Z. et al. Comparative genome analysis of 52 fish species suggests differential associations of repetitive elements with their living aquatic environments. *BMC Genomics* **19**, 1–10 (2018). 29439662 10.1186/s12864-018-4516-1 PMC5811955

[116] Pasquesi, G. I. M. et al. Squamate reptiles challenge paradigms of genomic repeat element evolution set by birds and mammals. *Nat. Commun.* **9**, 1–11 (2018). 30018307 10.1038/s41467-018-05279-1 PMC6050309

[117] Casacuberta, E. & González, J. The impact of transposable elements in environmental adaptation. *Mol. Ecol.* **22**, 1503–1517 (2013). 23293987 10.1111/mec.12170

[118] Bista, I. et al. Genomics of cold adaptations in the Antarctic notothenioid fish radiation. *Nat. Commun.* **14**, 1–16 (2023). 37296119 10.1038/s41467-023-38567-6 PMC10256766

[119] Schrader, L. & Schmitz, J. The impact of transposable elements in adaptive evolution. *Mol. Ecol.* **28**, 1537–1549 (2019). 30003608 10.1111/mec.14794

[120] Marcionetti, A. & Salamin, N. Insights into the genomics of clownfish adaptive radiation: the genomic substrate of the diversification. *Genome Biol. Evol.* **15**, 1–16 (2023). 10.1093/gbe/evad088 PMC10349533 37226990

[121] Carotti, E. et al. Transposable elements and teleost migratory behaviour. *Int. J. Mol. Sci.* **22**, 1–12 (2021). 10.3390/ijms22020602 PMC7827017 33435333

[122] Cerca, J. et al. No evidence of transposable element bursts in the Galápagos *Scalesia* adaptive radiation despite hybridization, diversification and ecological niche shifts. *Mob. DNA* **16**, 1–14 (2025). 40450335 10.1186/s13100-025-00362-z PMC12125827

[123] Buser, T. J., Sidlauskas, B. L. & Summers, A. P. 2D or Not 2D? testing the utility of 2D Vs. 3D landmark data in geometric morphometrics of the sculpin subfamily Oligocottinae (Pisces; Cottoidea). *Anat. Rec.* **301**, 806–818 (2018). 10.1002/ar.23752 29244247

[124] Evans, K. M. et al. Beaks promote rapid morphological diversification along distinct evolutionary trajectories in labrid fishes (Eupercaria: Labridae). *Evolution* **77**, 1–15 (2023). 37345732 10.1093/evolut/qpad115

[125] Evans, K. M. et al. Integration drives rapid phenotypic evolution in flatfishes. *Proc. Natl. Acad. Sci. USA* **118**, 1–10 (2021). 10.1073/pnas.2101330118 PMC8106320 33931506

[126] Hughes, L. C. et al. Comprehensive phylogeny of ray-finned fishes (Actinopterygii) based on transcriptomic and genomic data. *Proc. Natl. Acad. Sci. USA* **115**, 6249–6254 (2018). 29760103 10.1073/pnas.1719358115 PMC6004478

[127] Betancur-R, R. et al. The tree of life and a new classification of bony fishes. *PLoS Curr.* **5**, 0–45 (2013). 10.1371/currents.tol.53ba26640df0ccaee75bb165c8c26288 PMC3644299 23653398

[128] Broughton, R. E., Betancur-R., R., Li, C., Arratia, G. & Ortí, G. Multi-locus phylogenetic analysis reveals the pattern and tempo of bony fish evolution. *PLoS Curr.* **5**, ecurrents.tol.2ca8041495ff afd0c92756e75247483e (2013). 10.1371/currents.tol.2ca8041495ffafd0c92756e75247483e PMC3682800 23788273

[129] Li, C., Ortí, G., Zhang, G. & Lu, G. A practical approach to phylogenomics: the phylogeny of ray-finned fish (Actinopterygii) as a case study. *BMC Evol. Biol.* **7**, 1–11 (2007). 17374158 10.1186/1471-2148-7-44 PMC1838417

[130] Ranwez, V., Douzery, E. J. P., Cambon, C., Chantret, N. & Delsuc, F. MACSE v2: toolkit for the alignment of coding sequences accounting for frameshifts and stop codons. *Mol. Biol. Evol.* **35**, 2582–2584 (2018). 30165589 10.1093/molbev/msy159 PMC6188553

[131] Yang, C. et al. Efficient COI barcoding using high throughput single-end 400 bp sequencing. *BMC Genomics* **21**, 1–10 (2020). 10.1186/s12864-020-07255-w PMC7716423 33276723

[132] Kearse, M. et al. Geneious Basic: an integrated and extendable desktop software platform for the organization and analysis of sequence data. *Bioinformatics* **28**, 1647–1649 (2012). 22543367 10.1093/bioinformatics/bts199 PMC3371832

[133] Borowiec, M. L. AMAS: a fast tool for alignment manipulation and computing of summary statistics. *PeerJ.* **4**, e1660 (2016). 26835189 10.7717/peerj.1660 PMC4734057

[134] Price, M. N., Dehal, P. S. & Arkin, A. P. FastTree 2 - Approximately maximum-likelihood trees for large alignments. *PLoS ONE* **5**, e9490 (2010). 20224823 10.1371/journal.pone.0009490 PMC2835736

[135] Ludt, W. B., Bernal, M. A., Kenworthy, E., Salas, E. & Chakrabarty, P. Genomic, ecological, and morphological approaches to investigating species limits: a case study in modern taxonomy from Tropical Eastern Pacific surgeonfishes. *Ecol. Evol.* **9**, 4001–4012 (2019). 31015983 10.1002/ece3.5029 PMC6467843

[136] Lanfear, R., Frandsen, P. B., Wright, A. M., Senfeld, T. & Calcott, B. Partitionfinder 2: new methods for selecting partitioned models of evolution for molecular and morphological phylogenetic analyses. *Mol. Biol. Evol.* **34**, 772–773 (2017). 28013191 10.1093/molbev/msw260

[137] Kozlov, A. M., Darriba, D., Flouri, T., Morel, B. & Stamatakis, A. RAxML-NG: a fast, scalable and user-friendly tool for maximum likelihood phylogenetic inference. *Bioinformatics* **35**, 4453–4455 (2019). 31070718 10.1093/bioinformatics/btz305 PMC6821337

[138] Minh, B. Q., Hahn, M. W. & Lanfear, R. New methods to calculate concordance factors for phylogenomic datasets. *Mol. Biol. Evol.* **37**, 2727–2733 (2020). 32365179 10.1093/molbev/msaa106 PMC7475031

[139] Near, T. J. et al. Phylogeny and tempo of diversification in the superradiation of spiny-rayed fishes. *Proc. Natl. Acad. Sci. USA* **110**, 12738–12743 (2013). 23858462 10.1073/pnas.1304661110 PMC3732986

[140] Rabosky, D. L. et al. An inverse latitudinal gradient in speciation rate for marine fishes. *Nature* **559**, 392–395 (2018). 29973726 10.1038/s41586-018-0273-1

[141] Rambaut, A., Drummond, A. J., Xie, D., Baele, G. & Suchard, M. A. Posterior summarization in Bayesian phylogenetics using Tracer 1.7. *Syst. Biol.* **67**, 901–904 (2018). 29718447 10.1093/sysbio/syy032 PMC6101584

[142] Drummond, A. J. & Rambaut, A. BEAST: Bayesian evolutionary analysis by sampling trees. *BMC Evol. Biol.* **7**, 1–8 (2007). 17996036 10.1186/1471-2148-7-214 PMC2247476

[143] IUCN. *The IUCN Red List of Threatened Species* https://www.iucnredlist.org (2021).

[144] OBIS. D*ata from the Ocean Biogeographic Information System* (Intergovernmental Oceanographic Commission of UNESCO, 2021).

[145] Kulbicki, M. et al. Global biogeography of reef fishes: a hierarchical quantitative delineation of regions. *PLoS ONE* **8**, e81847 (2013). 24386083 10.1371/journal.pone.0081847 PMC3875412

[146] Spalding, M. D. et al. Marine ecoregions of the world: a bioregionalization of coastal and shelf areas. *Bioscience* **57**, 573–583 (2007).

[147] Hodge, J. R. et al. Constraints on the ecomorphological convergence of Zooplanktivorous Butterflyfishes. *Integr. Org. Biol.* **3**, 1–20 (2021). 10.1093/iob/obab014 PMC8341894 34377941

[148] Ree, R. H. & Smith, S. A. Maximum likelihood inference of geographic range evolution by dispersal, local extinction, and cladogenesis. *Syst. Biol.* **57**, 4–14 (2008). 18253896 10.1080/10635150701883881

[149] Ronquist, F. Dispersal-vicariance analysis: a new approach to the quantification of historical biogeography. *Syst. Biol.* **46**, 195–203 (1997).

[150] Landis, M. J., Matzke, N. J., Moore, B. R. & Huelsenbeck, J. P. Bayesian analysis of biogeography when the number of areas is large. *Syst. Biol.* **62**, 789–804 (2013). 23736102 10.1093/sysbio/syt040 PMC4064008

[151] Matzke, N. J. Model selection in historical biogeography reveals that founder-event speciation is a crucial process in island clades. *Syst. Biol.* **63**, 951–970 (2014). 25123369 10.1093/sysbio/syu056

[152] Dupin, J. et al. Bayesian estimation of the global biogeographical history of the Solanaceae. *J. Biogeogr.* **44**, 887–899 (2017).

[153] O’Dea et al. Formation of the isthmus of panama. *Sci. Adv.* **2**, 1–12 (2016). 10.1126/sciadv.1600883 PMC4988774 27540590

[154] Matzke, N. J. *BioGeoBEARS - Run BioGeoBEARS on Multiple Trees* https://github.com/nmatzke/BioGeoBEARS/blob/master/R/BioGeoBEARS_on_multiple_trees_v1.R (2019).

[155] Wickham, H. *Ggplot2: Elegant Graphics for Data Analysis* (Springer- Verlag, New York, 2016).

[156] Cheng, H. et al. Haplotype-resolved assembly of diploid genomes without parental data. *Nat. Biotechnol.* **40**, 1332–1335 (2022). 35332338 10.1038/s41587-022-01261-x PMC9464699

[157] Challis, R., Richards, E., Rajan, J., Cochrane, G. & Blaxter, M. BlobToolKit - interactive quality assessment of genome assemblies. *G3: Genes, Genomes, Genet.* **10**, 1361–1374 (2020). 10.1534/g3.119.400908 PMC7144090 32071071

[158] Guan, D. et al. Identifying and removing haplotypic duplication in primary genome assemblies. *Bioinformatics* **36**, 2896–2898 (2020). 31971576 10.1093/bioinformatics/btaa025 PMC7203741

[159] Lieberman-Aiden, E. et al. Comprehensive mapping of long-range interactions reveals folding principles of the human genome. *Science* **326**, 289–294 (2009). 19815776 10.1126/science.1181369 PMC2858594

[160] Putnam, N. H. et al. Chromosome-scale shotgun assembly using an in vitro method for long-range linkage. *Genome Res.* **26**, 342–350 (2016). 26848124 10.1101/gr.193474.115 PMC4772016

[161] Gurevich, A., Saveliev, V., Vyahhi, N. & Tesler, G. QUAST: quality assessment tool for genome assemblies. *Bioinformatics* **29**, 1072–1075 (2013). 23422339 10.1093/bioinformatics/btt086 PMC3624806

[162] Flynn, J. M. et al. RepeatModeler2 for automated genomic discovery of transposable element families. *Proc. Natl. Acad. Sci. USA* **117**, 9451–9457 (2020). 32300014 10.1073/pnas.1921046117 PMC7196820

[163] Smit, A. F. A., Hubley, R. & Green, P. *RepeatMasker Open-4.0* http://www.repeatmasker.org (2015).

[164] DiBattista, J. D. et al. Draft genome of an iconic Red Sea reef fish, the blacktail butterflyfish (*Chaetodon austriacus*): current status and its characteristics. *Mol. Ecol. Resour.* **18**, 347–355 (2018). 27488138 10.1111/1755-0998.12588

[165] Stanke, M. et al. AUGUSTUS: Ab initio prediction of alternative transcripts. *Nucleic Acids Res.* **34**, 435–439 (2006). 10.1093/nar/gkl200 PMC1538822 16845043

[166] Dobin, A. et al. STAR: ultrafast universal RNA-seq aligner. *Bioinformatics* **29**, 15–21 (2013). 23104886 10.1093/bioinformatics/bts635 PMC3530905

[167] Holt, C. & Yandell, M. MAKER2: an annotation pipeline and genome-database management tool for second-generation genome projects. *BMC Bioinforma.* **12**, 1–14 (2011). 10.1186/1471-2105-12-491 PMC3280279 22192575

[168] Bateman, A. UniProt: a worldwide hub of protein knowledge. *Nucleic Acids Res.* **47**, D506–D515 (2019). 30395287 10.1093/nar/gky1049 PMC6323992

[169] Chan, P. P. & Lowe, T. M. tRNAscan-SE: searching for tRNA genes in genomic sequences. *Methods Mol. Biol.* **1962**, 1–29 (2019). 31020551 10.1007/978-1-4939-9173-0_1 PMC6768409

[170] Emms, D. M. & Kelly, S. OrthoFinder: phylogenetic orthology inference for comparative genomics. *Genome Biol.* **20**, 1–14 (2019). 31727128 10.1186/s13059-019-1832-y PMC6857279

[171] Shen, W., Sipos, B. & Zhao, L. SeqKit2: a Swiss army knife for sequence and alignment processing. *iMeta* **3**, 1–5 (2024). 10.1002/imt2.191 PMC11183193 38898985

[172] Wheeler, T. J. & Eddy, S. R. Nhmmer: DNA homology search with profile HMMs. *Bioinformatics* **29**, 2487–2489 (2013). 23842809 10.1093/bioinformatics/btt403 PMC3777106

[173] Jordan, G. & Goldman, N. The effects of alignment error and alignment filtering on the sitewise detection of positive selection. *Mol. Biol. Evol.* **29**, 1125–1139 (2012). 22049066 10.1093/molbev/msr272

[174] Conway, J. R., Lex, A. & Gehlenborg, N. UpSetR: an R package for the visualization of intersecting sets and their properties. *Bioinformatics* **33**, 2938–2940 (2017). 28645171 10.1093/bioinformatics/btx364 PMC5870712

[175] Kowalczyk, A. et al. RERconverge: an R package for associating evolutionary rates with convergent traits. *Bioinformatics* **35**, 4815–4817 (2019). 31192356 10.1093/bioinformatics/btz468 PMC6853647

[176] Benson, G. Tandem repeats finder: a program to analyze DNA sequences. *Nucleic Acids Res.* **27**, 573–580 (1999). 9862982 10.1093/nar/27.2.573 PMC148217

[177] Bao, W., Kojima, K. K. & Kohany, O. Repbase update, a database of repetitive elements in eukaryotic genomes. *Mob. DNA* **6**, 4–9 (2015). 26045719 10.1186/s13100-015-0041-9 PMC4455052

[178] Santaquiteria, A. et al. Data for: Ecological and genomic signatures of the convergent evolution of planktivory in fossil and living reef fishes over deep time. 10.6084/m9.figshare.29594255 (2026). 10.1038/s41467-026-73110-3 PMC13385881 42173838

