# Supplementary material for: Ecological and genomic signatures of the convergent evolution of planktivory in fossil and living reef fishes over deep time
Source: Nat Commun. 2026 May 22;17:6739. doi: 10.1038/s41467-026-73110-3 (PMC13385881; doi:10.1038/s41467-026-73110-3)
Supplement: Supplementary file 1 — Supplementary information [file 41467_2026_73110_MOESM1_ESM.pdf]

Supplementary Information

Ecological and genomic signatures of the convergent evolution of planktivory in fossil and living reef fishes over deep time

Aintzane Santaquiteria *et al.*

Supplementary Note 1: Morphological characters

List of 107 osteological characters (including synapomorphies, autapomorphies, and other character states) observed in juvenile and adult (but not larval) stages, used to distinguish generic taxa among acanthuriforms and their immediate outgroups. Characters were compiled from the following sources: [1–13], and personal observations by J.C. Tyler and G. Carnevale.

Skull

1) Skull bone surfaces. Relatively smooth to variously striated or ridged but not cancellous=0; cancellous, especially the frontal and supraoccipital, with pitted indentations and/or spongy-like deep sculpturing, connective tissue or fatty filled=1.

2) Ascending process of premaxilla, length. Moderate, about one-third to two-thirds length of alveolar process, whether to front edge of block-like ethmoid or to level of approximately one-third to one-half back on more elongate ethmoid=0; short, less than one-third length of alveolar process, not reaching behind anterior end of ethmoid=1; long, usually reaching more than one-half back on ethmoid=2.

3) Maxilla-premaxilla articulation. Moveable=0; slightly moveable=1; relatively immoveable=2.

4) Jaw protrusibility. Protrusibility well developed=0; only slightly protrusible=1; essentially no protrusibility, rotating around ethmoid (presumed for fossils based on premaxilla and maxilla conditions)=2.

5) Articular, size relative to dentary. Articular distinctly longer and larger than dentary=0; articular about equal in length and size to dentary=1; articular distinctly smaller than dentary=2.

6) Articular/dentary articulation. Broad anterior surface of articular attached firmly to dentary=0; area of contact between articular and dentary much reduced and joint flexible=1.

7) Palatine, position, and articulation. At least posterior portion of palatine alongside or immediately in front of lateral ethmoid and articulated to it, usually by a condylar process=0; palatine lies well forward of lateral ethmoid and no articulation with it=1.

8) Mesethmoid, position. Mostly situated anterior to lateral ethmoids, often with a convex and usually wedge-shaped surface anteriorly along which rostral cartilage slides during protrusion of upper jaw=0; not extended forward of lateral ethmoids and inverted posteriorly with lateral walls that extend back into orbit to accommodate ascending processes of premaxillae=1.

9) Frontal, spiny process. Absent=0; a conical spine-like protuberance or prominent bump present on each frontal near anterodorsal margin of orbit=1.

10) Ectopterygoid shape. Elongate and extending ventrally at least along about one-half of anterior edge of quadrate=0; greatly reduced in size, its ventral region extending only a short distance along anterior edge of quadrate=1.

11) Symplectic length. About 25% or more of length of ventrolateral margin of quadrate=0; reduced in length, about 8% to 15% of quadrate length=1; symplectic absent=2.

12) Symplectic shape. A simple straight cylindrical bone=0; with a gentle sigmoid flexure=1; not applicable= –.

13) Parietal. Present=0; absent=1.

14) Parasphenoid apophysis. Absent=0; present as a bony process or spur=1; present as a cartilage without bony process=2.

15) Preopercular margin. Serrate=0; smooth=1.

16) Infraorbitals, number. Six=0; seven=1; five=2; four=3; three=4; two=5.

17) Second infraorbital articulation with lachrymal. A close association between lachrymal and second infraorbital, and main body of lachrymal lies below the path of infraorbital ring=0; second infraorbital articulates loosely and less closely with lachrymal, often out of contact with one another, and point of connection is at posteroventral corner of lachrymal, and main body of lachrymal lies above the projected path of infraorbital ring=1; not applicable when second infraorbital absent.

18) Infraorbital series orientation and position, and lachrymal articulation. Lachrymal with a condylar articulation with lateral ethmoid, and its posterodorsal margin conforms to anteroventral border of orbit=0; infraorbital series turns anteriorly below lateral ethmoid and extends forward alongside of snout, with lachrymal displaced anteriorly and removed from anterior border of orbit, and not articulating with lateral ethmoid=1.

19) Suborbital shelf. Present=0; absent=1.

20) Supraoccipital crest, height when present. High, often forming triangular peak with thickened anterior edge=0; moderate height=1; absent or very low, often as short ridge=2.

21) Supraoccipital association with exoccipitals ventrally. Ventral extension of supraoccipital (spina occipitalis) embraced laterally by dorsal extensions of exoccipitals that are tightly attached to supraoccipital on each side of the extension=0; supraoccipital does not contact exoccipitals ventrally=1.

22) Supraoccipital, spina occipitalis. Well developed, extending ventrally between epioccipitals to dorsal margin of foramen magnum and embraced laterally by dorsal processes of exoccipitals=0; no spina occipitalis and epioccipitals meet synchondrally, broadly separating supraoccipital from exoccipitals and foramen magnum=1.

23) Hyomandibula, anterodorsal surface. Relatively flat and smooth=0; a distinct transverse ridge present=1.

24) Hyomandibula, dorsomedial flange, if present. Without a foramen=0; with a large foramen=1; not applicable= –.

25) Opercle, presence of dilator process at dorsal end. Poorly developed, and posterior edge of upper region of opercle slightly to moderately concave to moderately or distinctly convex=0; well developed as a tapering dorsal projection, and posterior edge of upper region of opercle only slightly convex to relatively straight=1.

26) Interopercle, shape. Approximately ovoid=0; broad posteriorly with dorsal portion continuing anteriorly as a narrow extension=1; or as a narrow ligamentous band =2.

Teeth

27) Teeth, articulation. Fixed, relatively inflexible=0; slightly bendable, flexible, often setiform, brush-like in multiple rows=1; especially bendable, flexible=2; teeth absent=3.

28) Teeth, shape. Relatively conical and of moderate length, with smooth edges at least in adults, and without prominent lobes, denticulate edges, or notches=0; compressed and spatulate through most of their length, with well-developed denticulations/lobations=1; mostly conical to somewhat compressed at least distally, with denticulations variously small, minute or absent=2; stout, compressed, with one or two prominent notches=3; slender, elongate, sometimes laterally compressed, tapering to the end and without prominent lobes, denticulations, and notches=4; slender, elongate, with small lobes on one side only of distal region=5; slender, elongate, with trident notching at distal end=6; relatively stoutly conical, with shallow to moderately deep notches or widely spaced indentations, sometimes only on posterodorsal edge=7; simple, greatly reduced in size or absent, at least in adults=8.

Hyoid arch and branchiostegals

29) Branchiostegals, number. Seven (3+4)=0; six (2+4)=1; five (1+4)=2; four (0+4)=3.

30) First branchiostegal ray, shape. Slender, almost straight to curved scimitar-shaped=0; somewhat broadened but scimitar-shaped=1; broadly flattened, thin, sometimes irregular shaped=2.

31) Urohyal, shape. Broad triangular outline, with a moderately concave and sometimes irregular posterior edge, and the greatest depth about equal to or greater than the length, with the somewhat thickened anteroventral edge not laterally expanded as a flange=0; narrow triangular outline, only moderately expanding in depth posteriorly from the articular head, much longer than deep, and ventral edge moderately to prominently laterally expanded as a flange of increasing width posteriorly=1; relatively square plate, somewhat concave along posterior edge, and moderately expanded laterally as a flange along anteroventral edge=2; deeply and broadly concave along posterior edge, often sickle-shaped, much higher than long, with distance from anterior articular to deepest edge of concavity one-half or less than height=3; elongate ovoid, gradually increasing moderately in depth from articular head to gently rounded posterior end=4; enormously expanded from articular region of lower jaw to ventral end of cleithrum, with a thickened area vertically in middle of broad lateral surface, and a relatively straight posterior edge=5.

Vertebral column
32) Vertebrae, total number. Twenty four=0; twenty three=1; twenty two=2; twenty five=3; twenty six=4; twenty seven=5; twenty eight=6; twenty nine to thirty=7.

33) Vertebrae, abdominal number. Ten=0; nine=1.

34) Vertebrae, caudal number. Fourteen=0; thirteen=1; nineteen to twenty=2.

35) Vertebral formula. 10+14=0; 10+13=1; 9+13=2; 10+19-20=3; 9+15=4; 10+12=5.

36) Neural spine of first vertebra. Autogenous=0; fixed to its centrum=1.

37) Vacant interneural spaces, number (in most specimens, if about equally frequent in several spaces recorded as polymorphic). None=0; one=1; two=2; three=3.

38) Vacant interneural space, when only one space vacant, location of that space. First space=0; second space=1; third space=2; fourth space=3; fifth space=4; sixth space=5; seventh space=6; eighth space=7; not applicable= –.

39) Vacant interneural spaces, when two or more spaces vacant, number of spaces. Two=0; three=1; not applicable= –.

40) Vacant interneural spaces, when two or more spaces vacant, number of groups of spaces. One=0; two=1; three=2; not applicable= –.

41) Supraneurals, number. Three or more=0; two=1; one=2; none=3.

42) Pleural ribs, presence on anterior abdominal vertebrae. Present on first and immediately following centra=0; absent on first centrum but present on second and immediately following centra=1; absent on first two centra but present on third and immediately following centra=2; absent on first three centra but present on fourth and immediately following centra=3.

43) Pleural ribs, most posterior rib, centrum to which articulated. Last abdominal centrum=0; penultimate abdominal centrum=1; antepenultimate=2.

44) Pleural ribs, most posterior rib, length and shape. Long and slender=0; moderate length and slender=1; moderate length and moderate width=2; short and broad, with its posterior edge situated internal to an anterolateral flange on haemal spine of first caudal vertebra=3; short, slender, and sometimes only a short rudiment that is difficult to determine in radiographs=4.

45) Epineurals. Present=0; absent=1.

46) Neural spine of first abdominal vertebra, length and shape. Long and slender=0; long and relatively broad=1; moderate length and slender=2; short and slender=3; short and broad=4.

47) Abdominal vertebrae parapophyses. Present on at least some of more posterior centra=0; essentially absent or very small=1.

48) Neural and haemal spines, thickness. Most neural and haemal spines robust and at least moderately wide=0; most neural and haemal spines (except sometimes for first two neurals and haemals) exceptionally slender=1.

Dorsal fin

49) Number of dorsal-fin spines. Zero=0; one=1; two=2; three=3; four=4; five=5; six=6; seven=7; eight=8; nine=9; ten=10; eleven=11; twelve=12; thirteen=13; fourteen=14.

50) Number of supernumerary dorsal spines (or soft rays if dorsal spines absent). Zero=0; one=1; two=2.

51) Dorsal-fin spines (or more anterior soft rays if dorsal spines absent), elongation. Not greatly elongated and filamentous distally=0; all but first two or three dorsal spines elongated and filamentous=1.

52) Number of soft dorsal rays. Zero to ten=0; eleven to twenty=1; twenty-one to thirty=2; thirty-one to forty=3; forty-one to fifty=4; fifty-one to sixty=5.

53) First dorsal spine (or first soft ray if dorsal spines absent/?). Visible externally, protruding through skin=0; not apparent externally, specialized as bony cap rotating on pterygiophore beneath skin=1; reduced to nubbin on surface of pterygiophore just in front of base of well-developed second spine=2.

54) First dorsal spine, when visible externally. Relatively long, at least one-half length of second spine (or of first soft ray if only one spine present)=0; relatively short, less than one-half length of second spine (or about one-half if second spine is relatively short)=1; not applicable= –.

55) First dorsal-fin pterygiophore, position of ventral shaft relative to interneural spaces (in most specimens; if about equally frequent in several spaces recorded as polymorphic). In preneural space, often to rear of skull=0; in first interneural space (or over shorten open neural spine of first vertebra)=1; in second interneural space=2; in third interneural space=3; in fourth interneural space=4.

56) Number of anterior dorsal-fin pterygiophores present in preneural space, when preneural space is occupied. Only the first spiny-dorsal pterygiophore=0; both the first and second spiny-dorsal pterygiophores=1; not applicable= –.

57) Dorsal fin pterygiophores, number in tenth interneural space. One=0; two=1; three=2.

58) Specialized locking mechanism of first dorsal-fin spine. No specialized mechanism involving a deep indentation in dorsal region of first pterygiophore (proximal radial) and a median flange that is ribbed or roughened around which deeply concave base of first dorsal spine can rotate and lock=0; moderately specialized mechanism present, with a slightly to moderately deep indentation (maximum depth of indentation at level of base of first dorsal spine, and not well below it), and

roughened or somewhat ribbed median flange=1; highly specialized mechanism, with an exceptionally deep (to well below level of base of first dorsal spine) indentation and a strongly ribbed median flange=2; not applicable when dorsal spines absent= –.

59) Number of dorsal-fin spines and median pterygial flanges involved when specialized locking mechanism present. One (first spine only)=0; three (first, second, and third spines)=1; not applicable= –.

60) Shape of anterodistal region of first pterygiophore (proximal radial) when most specialized first dorsal spine locking mechanism present (i.e., Acanthuridae). Anterodistal region only moderately if at all posterolaterally expanded and not encompassing basal region of first dorsal spine=0; prominently expanded posterolaterally and encompassing basal region of reduced first dorsal spine=1; not applicable= –.

61) Distal ends of dorsal- and anal-fin pterygiophores. Not forming a median truss around most of body=0; forming a median truss around most of body=1.

62) Dorsal- and anal-fin rays segmentation. Dorsal and anal rays segmented=0; unsegmented=1.

63) Dorsal, anal, caudal, pectoral, and pelvic rays, spinules. Rays of these fins without spinules=0; small spinules present on most fins laterally along all or most of their lengths=1.

64) Distal ends of dorsal- and anal-fin pterygiophores. Not exceptionally expanded laterally=0; prominently expanded laterally=1.

65) First two haemal spines, orientation to one another. Parallel or divergent to one another=0; middle regions curved toward one another, at least in adults=1.

Anal fin

66) Number of anal spines. Zero=0; one=1; two=2; three=3; four=4; five=5; six=6; seven=7; eight=8.

67) Number of supernumerary anal spines (or soft rays if anal spines absent). Zero=0; one=1; two=2.

68) Number of soft anal rays. Zero to ten=0; eleven to twenty=1; twenty-one to thirty=2; thirty-one to forty=3; forty-one to fifty=4; fifty-one to sixty=5.

69) First anal spine (or first ray if anal spines absent). Visible externally, protruding through skin=0; not apparent externally, specialized as bony cap rotating on pterygiophore beneath skin=1.

70) First anal spine, when visible externally. Relatively long, at least one-half length of second spine=0; relatively short, less than one-half length of second spine (or about one-half if second spine is relatively short)=1; not applicable= –.

71) Anal fin pterygiophores, number in first interhaemal space. One=0; two=1; three=2; four or five=3; six or seven=4.

72) Specialized locking mechanism of first anal-fin spine. No specialized mechanism involving a deep indentation in ventral region of first pterygiophore (proximal radial) and no median flange that is ribbed or roughened around which deeply concave base of first anal spine can rotate and lock=0; moderately specialized mechanism present, with a slightly to moderately deep indentation (maximum depth of indentation at level of base of first anal spine and not well above it), and roughened or somewhat ribbed median flange=1; highly specialized mechanism, with an exceptionally deep indentation (to well above level of base of first anal spine) and a strongly ribbed median flange=2; not applicable= –.

73) Shape of anterodistal region of first proximal radial (pterygiophore) when most specialized first anal spine locking mechanism is present (i.e., Acanthuridae). Anterodistal region only slightly if at all posterolaterally expanded and not encompassing basal region of first anal spine=0; moderately to prominently expanded posterolaterally and partially encompassing basal region of reduced first anal spine=1; not applicable= –.

74) First anal-fin pterygiophore, orientation. Relatively vertical=0; slightly to moderately oblique=1; highly oblique=2.

75) Distal end of first anal-fin pterygiophore. Not greatly prolonged or positioned anteriorly=0; greatly prolonged or positioned anteriorly=1.

Caudal fin and caudal skeleton

76) Caudal fin, number of principal rays. Seventeen=0; sixteen=1; fifteen=2; fourteen=3; thirteen=4; twelve=5.

77) Caudal fin, number of procurrent rays (largest number either below or above). Three or more=0; one or two=1; none=2.

78) Caudal fin shape. Rounded=0; truncate=1; somewhat concave=2; deeply and decidedly concave or forked=3.

79) Hypurostegy. None, or only slight overlapping=0; moderate overlapping=1; extensive overlapping, with hypural plate nearly fully covered by proximal ends of caudal-fin rays=2.

80) Caudal peduncle depth. Relatively deep (~6 to 12 times in SL, or between ~8 to 20% of SL)=0; moderate depth (~13 to 17 times in SL, or between ~6 and 8% of SL)=1; relatively slender (~18 to 21 or more times in SL, or between ~ 4 and 6% or less of SL)=2.

81) Hypurals one to four, number of separate elements in adults. Four=0; two, with hypurals 1+2 consolidated to one another and 3+4 consolidated to one another, and one or both of these plates fused to compound centrum=1; two, with hypurals 1+2 and 3+4 consolidated as in preceding state but with neither of these plates fused to compound centrum=2; one, with hypurals 1+4 consolidated with one another and fused to compound centrum=3; one, with hypurals 1+4 consolidated with

one another but not fused to compound centrum=4; three, with hypurals 1 and 2 separate and hypurals 3+4 consolidated with one another, and none these three elements fused to compound centrum=5.

82) Hypural five. Hypural 5 and the anterior uroneural remain autogenous=0; hypural five remains separate from hypural four but at least its anterior end fuses to the embracing uroneural pair which in turn fuses to the compound centrum=1.

83) Epurals, number of separate elements in adults. Three=0; two=1; one=2.

84) Parhypural foramen, posterior margin. Formed by anteroventral margin of first hypural=0; formed by a dorsally positioned flange of bone from parhypural that separates foramen from first hypural=1.

85) Hypurapophysis. Terminates as a more or less sharp pointed to blunt or knob-like process=0; terminates in a T-shaped expansion oriented about 45 degrees to horizontal=1; terminates as a T-shaped expansion oriented about horizontally=2.

86) Uroneurals (including stegural), number (exclusive of nubbins). Two pairs=0; one pair (halves may be fused together)=1; none=2.

87) Uroneurals, length of first pair. Long, extending posteriorly well beyond region of compound centrum, reaching to above uppermost hypural or second uroneural=0; short, not extending prominently beyond region of compound centrum and not reaching to above uppermost hypural=1.

88) Neural spine of second preural centrum (NPU2), length. Long=0; short=1.

89) Autogenous haemal spines on centra anterior to terminal centrum and parhypural. One (on HPU2)=0; two (on HPU2 and 3)=1; three (on HPU2, 3, and 4)=2; four (on HPU2, 3, 4, and 5)=3; none=4.

Pectoral girdle and pectoral fin

90) Postcleithrum, number of elements. Two=0; one=1,

91) Postcleithrum, shape. Relatively elongate and shaft-like=0; a large plate, greatly expanded posteriorly=1.

92) Postcleithrum, ventral extension. Distal end of postcleithrum not in contact with first anal-fin pterygiophore=0; distal end of postcleithrum contacting or in close association with anterior extension of first anal-fin pterygiophore=1.

93) Pectoral fin situated in about middle of body or lower=0; situated high on body=1.

94) Coracoid, postcoracoid process. Present=0; absent=1.

95) Supracleithrum, sensory canal. Present, with posttemporal canal joining main trunk lateral line through a short bony canal at dorsal end of supracleithrum=0; absent, with main trunk lateral line canal communicating directly with posttemporal=1.

Pelvic girdle and pelvic fin

96) Number of pelvic-fin spines. None (at least in adults; entire fin in some species may become rudimentary with increasing specimen size)=0; one (on outer side of fin rays)=1; spine and rays consolidated into an operculum ani in adults=2.

97) Number of soft pelvic-fin rays. None (at least in adults)=0; one=1; two=2; three=3; four=4; five=5; six=6.

98) Pelvis, posterior process (ishiac). Posterior extension of short to moderate length or essentially absent=0; a long posterior extension making contact and sutured to anterior extension of first anal-fin pterygiophore=1.

99) Subpelvic keel (anterior iliac process from anteroventral end of pelvis). Anterior process prominent, and area above it distinctly concave=0; anterior process only poorly developed, and area above it only slightly if at all concave=1; no anterior prong-like process and no distinct concave area along anteroventral region of pelvis=2.

100) Pelvis (basipterygium) greatest depth to length ratio (length from anterior end of either anterodorsal ascending pubic process or from anteroventral iliac process to posterior end of pelvis, including any posterior ishiac process, whichever measurement is longer). 56–65% or more=0; 46–55%=1; 36–45%=2; 26–35%=3; 21–25%=4; 16–20%=5; 11–15%=6; 6–10%=7.

Scales

101) Scales. Ctenoid=0; spinoid/spinous (with upright or posterior edge non-articulated spinules or large granulations or hillocks)=1; cycloid=2; apparently without scales=3.

102) Greatly enlarged specialized scales on caudal peduncle. Absent=0; present either as fixed plates or a folding spine=1.

103) Greatly enlarged specialized scales on caudal peduncle, structure when present. Fixed plates=0; folding spine in a groove of variable depths and widths=1; not applicable= –.

104) Greatly enlarged specialized scales on caudal peduncle, when present as fixed plates. One or two fixed plates=0; three or more fixed plates=1; not applicable=–.

105) Tubercular scale plates on snout. Absent=0; large (approximately one-half pupil diameter, or about one-fourth to one-fifth orbit diameter) tubercular scale plates present on the snout between eye and upper jaw=1.

106) Somewhat enlarged scales (approximately twice or more as large as the surrounding small body scales) on the body between the posterior regions of the soft dorsal and anal fin bases and anterior caudal peduncle. Absent=0; present=1; not applicable when scaleless= –.

Miscellaneous

107) Anus, position (in fossils inferred as just in front of anterodistal end of first anal-fin pterygiophore). Anus not much displaced anteriorly=0; anus moderately displaced anteriorly=1; anus displaced far forward=2.

Supplementary figures and tables

Supplementary Table 1. Legacy markers and their corresponding exon IDs that are present in the FishLife dataset and/or in the sequences downloaded from GenBank.

Legacy marker	Exon ID	FishLife	GenBank
TBR1	E1541	YES	NO
RAG1	E1684	YES	YES
KIAA1239	E1728	YES	NO
MYH6	E1730	YES	YES
ENC1	E1732	YES	NO
PLAGL2	E1735	YES	YES
RIPK4	E1737	YES	NO
SH3PX3	E1738	YES	NO
SIDKEY	E1739	YES	NO
SREB2	E1740	YES	NO
ZIC1	E1741	YES	YES
SVEP1	E1746	YES	NO
GPR61	E1747	YES	NO
IRBP	E1748	YES	NO
RNF213	E1749	YES	NO
RHOD	E1750	YES	YES
UBEA3	E1752	YES	NO
UBEA3like (modified)	E1753	YES	NO
COI	Mitochondrial DNA	YES	YES
CYTB	Mitochondrial DNA	YES	YES
12S	Mitochondrial DNA	NO	YES
16S	Mitochondrial DNA	NO	YES

Supplementary Table 2. List of fossils used in this study, including their estimated ages, calibration distributions, and geographic and stratigraphic localities.

Fossil	Age (Ma)	Calibration distribution	Locality	References
Eozanclus brevirostris	48.5–50.5	Uniform	Bolca; late Ypresian	[14]
Angiolinia mirabilis	48.5–50.5	Uniform	Bolca; late Ypresian	[15]
Massalongius gazolai	48.5–50.5	Uniform	Bolca; late Ypresian	[16]
Gazolaichthys vestenanovae	48.5–50.5	Uniform	Bolca; late Ypresian	[17,18]
Eonaso deani	23–28.5	Uniform	Oliver Nugent quarry, Antigua and Barbuda; Oligocene	[19]
Tauichthys padremenini	48.5–50.5	Uniform	Bolca; late Ypresian	[20]
Tauichthys aspesae	48.5–50.5	Uniform	Bolca; late Ypresian	[13]
Pesciaraichthys punctatus	48.5–50.5	Uniform	Bolca; late Ypresian	[17]
Frigosorbiniae baldwinae	48.5–50.5	Uniform	Bolca; late Ypresian	[21]
Protozebrasoma bloti	48.5–50.5	Uniform	Bolca; late Ypresian	[21]
Arambourgthurus scombrurus	34		Istehbanat, Iran; late Eocene	[22]
Sorbinithurus sorbinii	48.5–50.5	Uniform	Bolca; late Ypresian	[20]
Marosichthys huismani	16–20.4	Uniform	Patoenoeang Asoe, Tonasa Formation, Sulawesi, Indonesia; Burdigalian	[23]
Glarithurus friedmani	30	Fixed	Landesplattenberg slate quarry, Matt Formation, Canton Glarus, Switzerland; Rupelian	[3]
Caprovesposus parvus	38.4–42.1	Uniform	Kuma Horizon (Kuma Formation), Gumista River near Sukumi, Abkhazia, Georgia; middle-late Eocene	[24]
Padovathurus gaudryi	48.5–50.5	Uniform	Bolca; late Ypresian	[16]
Proacanthurus bonatoi	48.5–50.5	Uniform	Bolca; late Ypresian	[17]
Proacanthurus elongatus	48.5–50.5	Uniform	Bolca; late Ypresian	[17]
Proacanthurus ovalis	48.5–50.5	Uniform	Bolca; late Ypresian	[25]
Proacanthurus tenuis	48.5–50.5	Uniform	Bolca; late Ypresian	[25]
Eorandallius rectifrons	48.5–50.5	Uniform	Bolca; late Ypresian	[25]
Eorandallius elegans	48.5–50.5	Uniform	Bolca; late Ypresian	[17]
Tylerichthys nuchalis	48.5–50.5	Uniform	Bolca; late Ypresian	[25]
Tylerichthys milani	48.5–50.5	Uniform	Bolca; late Ypresian	[17]
Metaspisurus emmanueli	48.5–50.5	Uniform	Bolca; late Ypresian	[17]
Lehmanichthys lessiniensis	48.5–50.5	Uniform	Bolca; late Ypresian	[17]

Acanthuroides massalongoi	48.5–50.5	Uniform	Bolca; late Ypresian	17
Luvarus necopinatus	55.8	Fixed	Uylya-Kushlyuk, Danatinsk Formation, Turkmenistan; basal Eocene	4
Avitoluvarus eocaenicus	38.4–42.1	Uniform	Left bank of Pshekha River, about 0.5 km from the Gorny Luch farmstead, Apsheronsk District, Krasnodar Region, Kuma Formation, North Caucasus, Russia; upper part of the Middle Eocene	26
Avitoluvarus dianae	55.8	Fixed	Uylya-Kushlyuk, Danatinsk Formation, Turkmenistan; basal Eocene	4
Avitoluvarus mariannae	55.8	Fixed	Uylya-Kushlyuk, Danatinsk Formation, Turkmenistan; basal Eocene	4
Kushlukia permira	55.8	Fixed	Uylya-Kushlyuk, Danatinsk Formation, Turkmenistan; basal Eocene	4

Supplementary Table 3. Ages of Acanthuriformes (crown and stem) estimated by previous studies.

Study	Crown Acanthuriformes	Acanthuriformes + Chaetodontidae	Acanthuriformes + Ephippidae	Acanthuriformes + (Chaetodontidae+ Ephippidae)	Acanthuridae	Comments
Alfaro *et al.* (2018)[27]		68 (63–72)			31 (16–52)*	No Ephippidae, Luvaridae, Zanclidae. One species per genus
Betancur *et al.* (2013)[28]	55*	70	100			
Betancur *et al.* (2017)[29]	64			90	50	
Ghezelayagh *et al.* (2022)[30]	59 (55–65)	69 (62–74)	76 (72–82)			
Hughes *et al.* (1018)[31]		88 (77–90)*	90 (89–95)			
Near *et al.* (2012)[32]	64	75			60	No Ephippidae
Near *et al.* (2013)[33]	61	68			51	No Ephippidae
Rabosky *et al.* (2018)[34]	77.8*		96.1		57.7	
Siqueira *et al.* (2019)[35]					80.9 (67.5–95.6)*	Using Zanclidae and Luvaridae as outgroup
Sorenson *et al.* (2013)[36]					54 (51–62)	Using Zanclidae and Luvaridae as outgroup

* = outliers

Supplementary Table 4. MrBayes runs and convergence statistics for each subset and scheme (Scheme 1 and Scheme 2).

Type of subset	Number of generations for each run	Number of generations combined	Estimated sample size (ESS) values
Null test, Subset1, No constraints, no root	265,930,000	1,914,720,000	>204 ESS
Null test, Subset1, No constraints, Second calibration at the root (stem Acanthuriformes)	209,560,000	1,508,880,000	>123 ESS
Subset1, Scheme1, With constraints, Secondary calibration at the root (crown Acanthuriformes)	400,000,000	2,880,000,000	>166 ESS
Subset2, Scheme1, With constraints, Secondary calibration at the root (crown Acanthuriformes)	347,310,000	2,500,640,000	>203 ESS
Subset3, Scheme1, With constraints, Secondary calibration at the root (crown Acanthuriformes)	379,300,000	2,739,960,000	>257 ESS
Subset4, Scheme1, With constraints, Secondary calibration at the root (crown Acanthuriformes)	285,900,000	2,778,480,000	>220 ESS
Subset5, Scheme1, With constraints, Secondary calibration at the root (crown Acanthuriformes)	333,710,000	2,402,720,000	>168 ESS
Subset1, Scheme2, With constraints, Secondary calibration at the root (crown Acanthuriformes)	400,000,000	2,880,000,000	>234 ESS
Subset2, Scheme2, With constraints, Secondary calibration at the root (crown Acanthuriformes)	150,420,000	1,083,040,000	>58 ESS
Subset3, Scheme2, With constraints, Secondary calibration at the root (crown Acanthuriformes)	389,420,000	2,803,840,000	>151 ESS
Subset4, Scheme2, With constraints, Secondary calibration at the root (crown Acanthuriformes)	349,050,000	2,513,200,000	>117 ESS
Subset5, Scheme2, With constraints, Secondary calibration at the root (crown Acanthuriformes)	360,500,000	2,595,560,000	>162 ESS

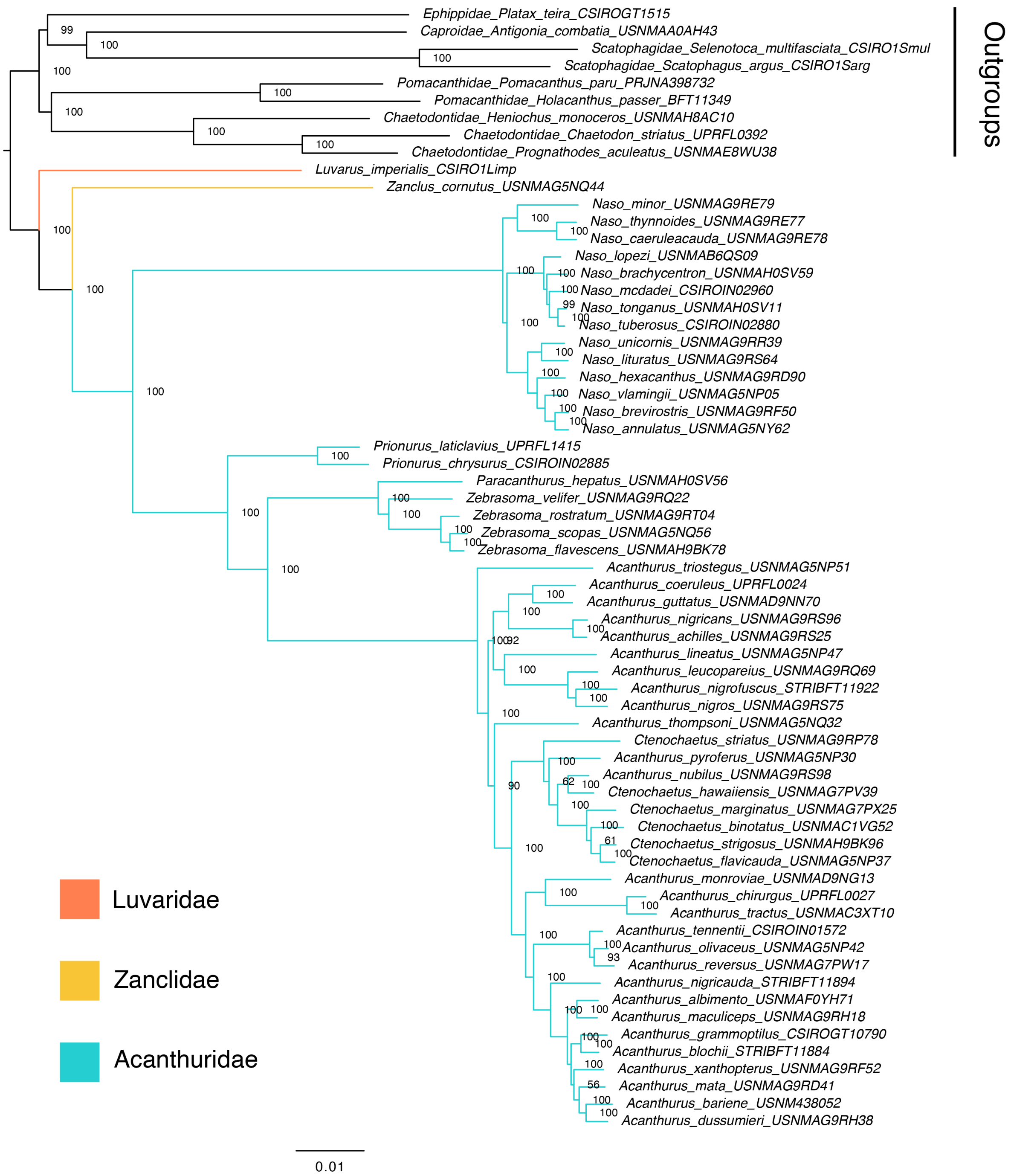

Supplementary Figure 1. Phylogeny of Acanthuriformes based on concatenation analysis of the reduced matrix comprised by 998 loci and 66 species (56 Acanthuriformes, 9 outgroups). Phylogenetic tree inferred with RAxML using the best-fit partition scheme identified with PartitionFinder for all newly sequenced taxa. Colors indicate families; nodal values indicate bootstrap support.

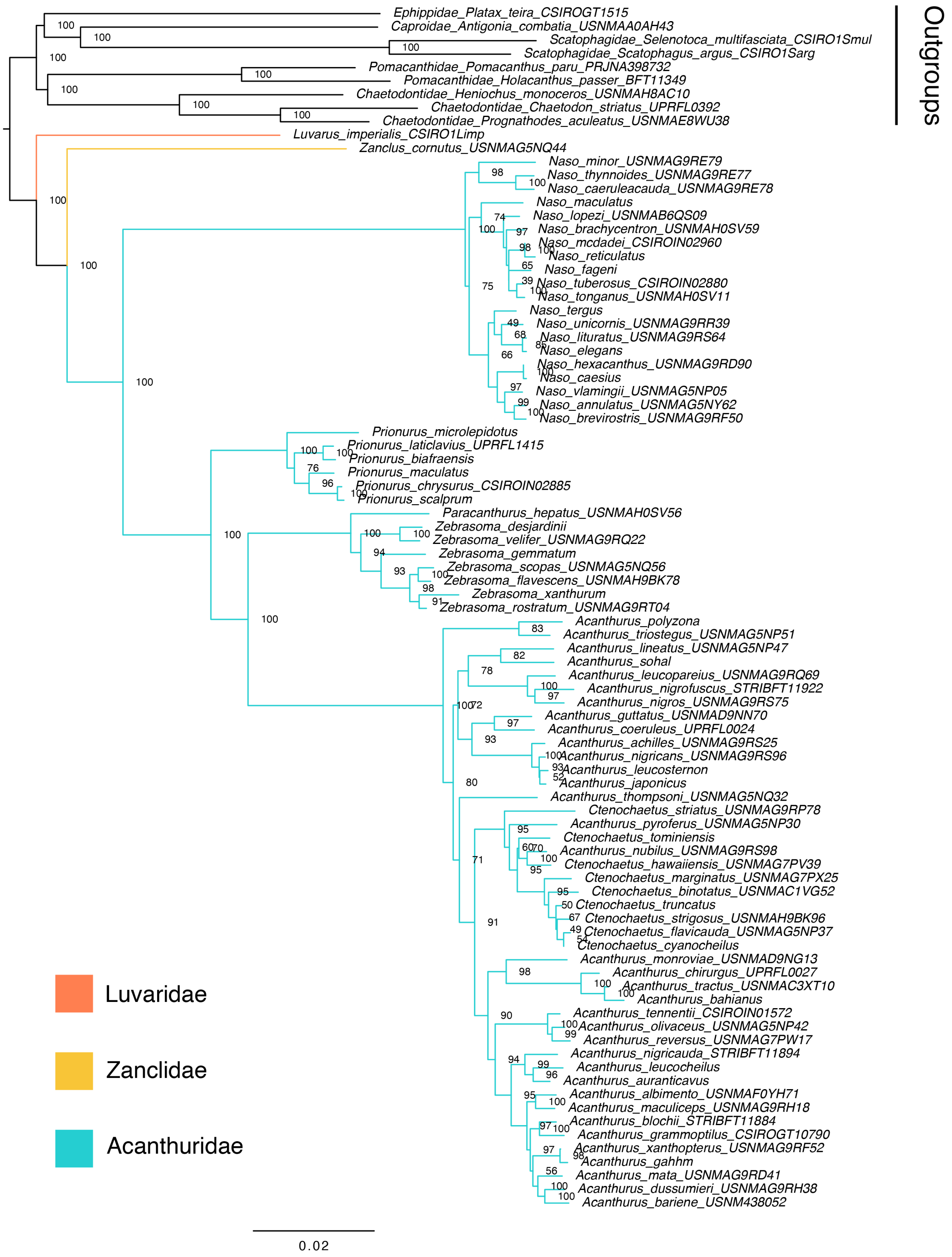

Supplementary Figure 2. Phylogeny of Acanthuriformes based on concatenation analysis of the expanded matrix comprised by 999 loci and 89 species (80 Acanthuriformes, 9 outgroups). Phylogenetic tree inferred with RAxML using the best fit partition scheme identified with PartitionFinder for all newly sequenced taxa. Colors indicate families; nodal values indicate bootstrap support.

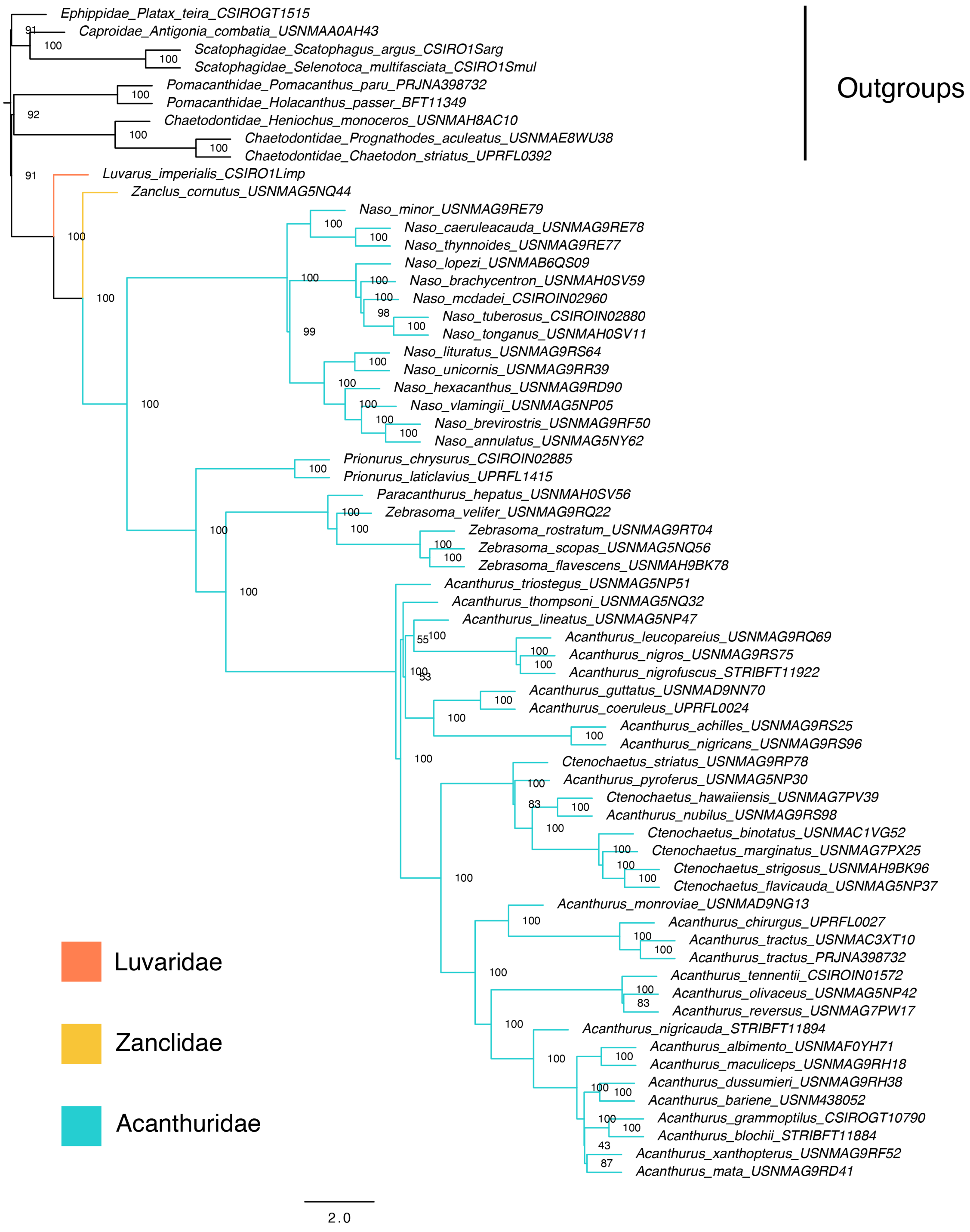

Supplementary Figure 3. Phylogeny of Acanthuriformes based on multi-species coalescent analysis of the reduced matrix comprised by 998 loci and 66 species (56 Acanthuriformes, 9 outgroups). Phylogenetic tree inferred with ASTRAL-III for all newly sequenced taxa. Colors indicate families; nodal values indication bootstrap support.

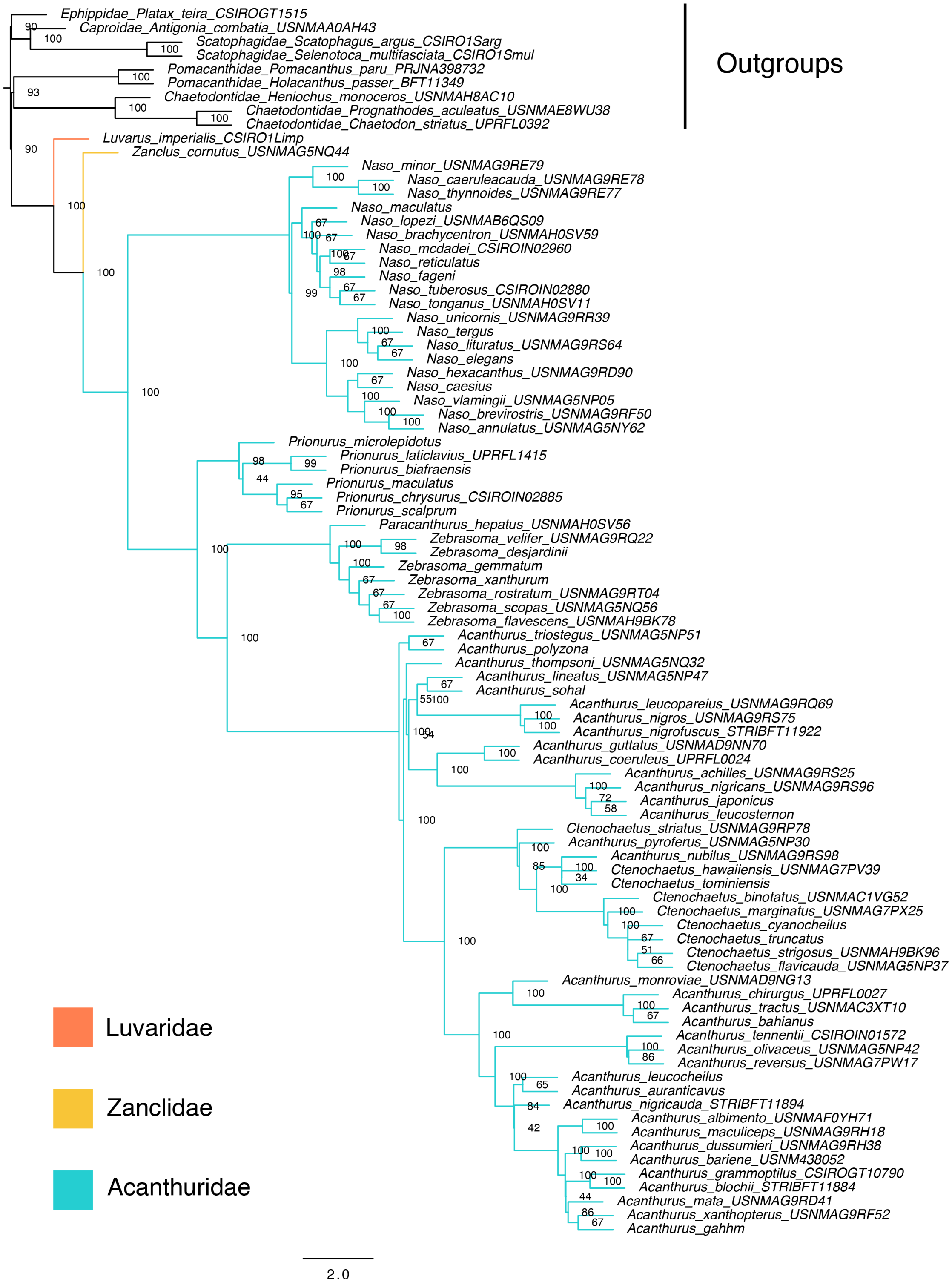

Supplementary Figure 4. Phylogeny of Acanthuriformes based on multi-species coalescent analysis of the expanded matrix comprised by 999 loci and 89 species (80 Acanthuriformes, 9 outgroups). Phylogenetic tree inferred with ASTRAL-III for all newly sequenced taxa. Colors indicate families. Nodal values indicate bootstrap support.

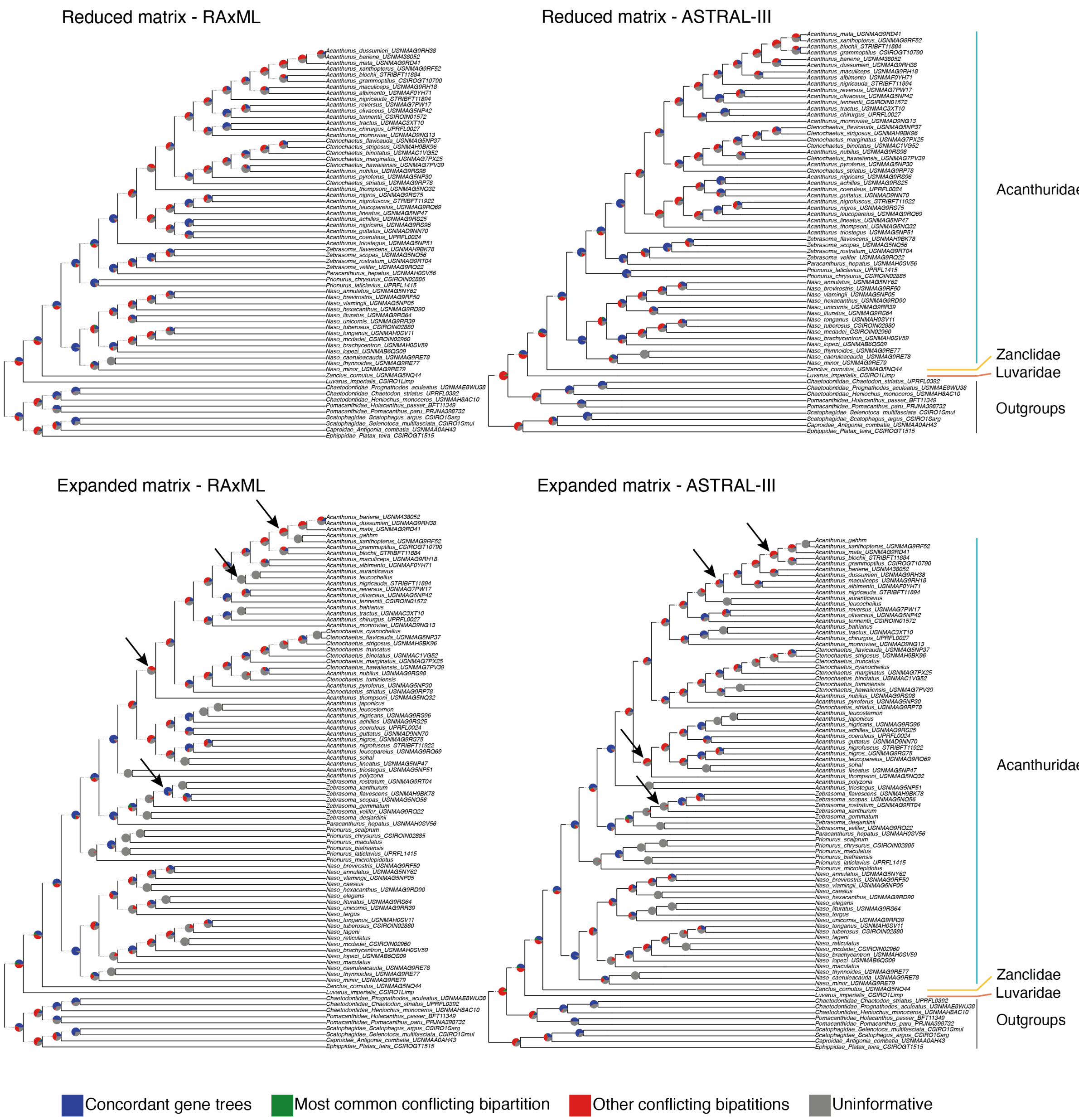

Supplementary Figure 5. Discordance among the phylogenetic trees inferred in RAxML and ASTRAL-III based on reduced and expanded matrices, and their corresponding gene trees. Pie charts at each node indicate the proportion of gene trees that support the illustrated clade (blue), the proportion that supports the most common conflicting bipartition (green), the proportion that supports other conflicting bipartitions (red), and the proportion that are uninformative for each branch with less 50% bootstrap support, representing gene trees with no information (gray). Arrows indicate the more unstable nodes among RAxML and ASTRAL-III trees with the expanded matrix. Gene concordance factor analyses based on only molecular data reveal several clades with consistent gene tree and species tree relationships (e.g., the genera *Naso*, *Zebrasoma* or *Ctenochaetus*), with few exceptions such as the placement of *Acanthurus thompsoni* or the clade containing the most recent common ancestor of *A. dussumieri* and *A. grammoptilus*.

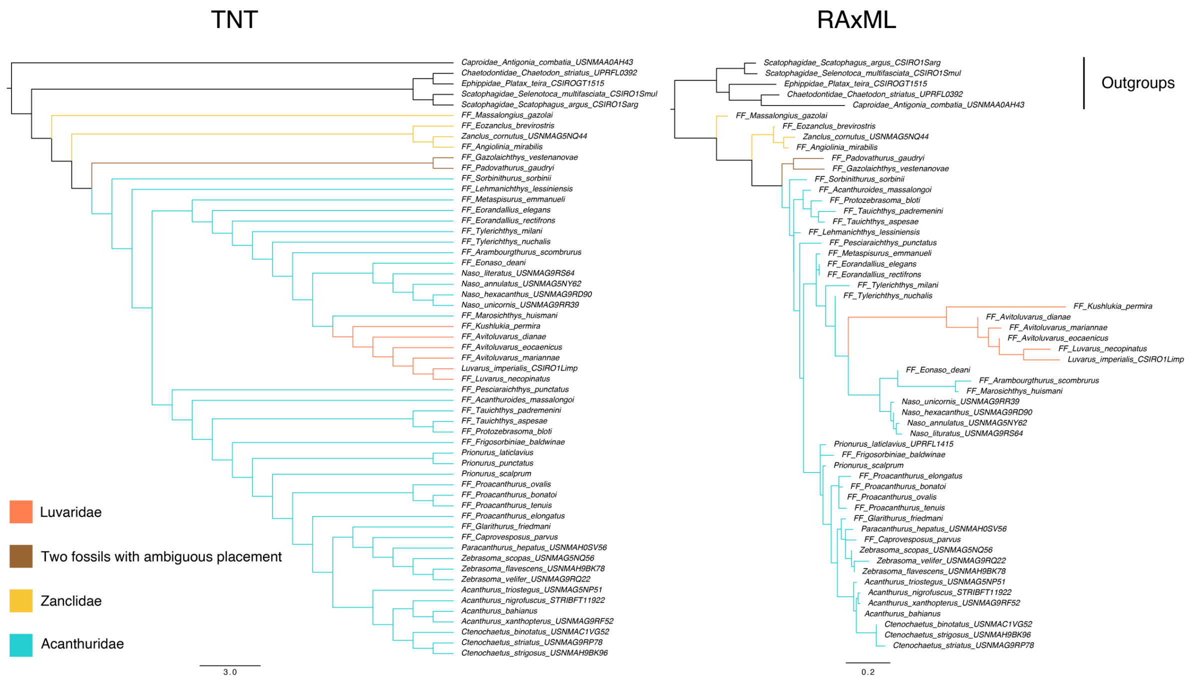

Supplementary Figure 6. Phylogenies of Acanthuriformes based on the morphological matrix comprised by 107 characters and 56 species (32 fossil + 19 extant Acanthuriformes, 5 outgroups). The phylogeny on the left was estimated using TNT (parsimony), while the one on the right was inferred with RAxML (maximum likelihood, MK model). Colors indicate different families. Note that the brown color represents two fossils with ambiguous placements in combined molecular and morphological analyses (see Materials and methods and Supplementary Figs. 7, 9-10).

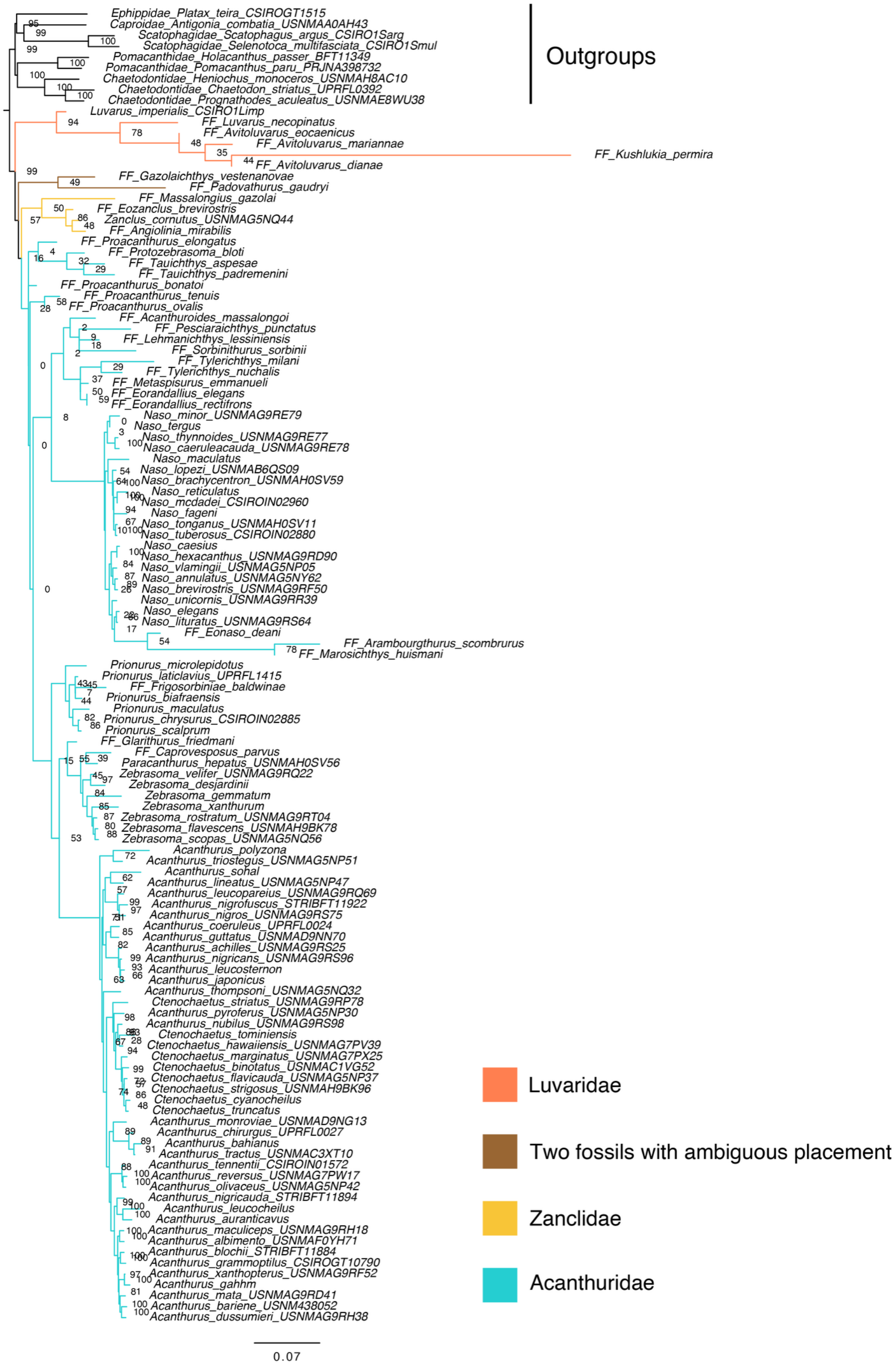

Supplementary Figure 7. Phylogeny of Acanthuriformes based on a concatenation analysis of the combined matrix comprised by 999 loci, 107 morphological characters and 121 species (32 fossils + 80 extant Acanthuriformes, 9 outgroups). Phylogenetic tree was inferred with RAxML using the MK model for the morphological partition, and the MULTIGAMMA model for the DNA sequences using five by-gene and by-codon partitions. Colors indicate families. Nodal values indicate bootstrap support.

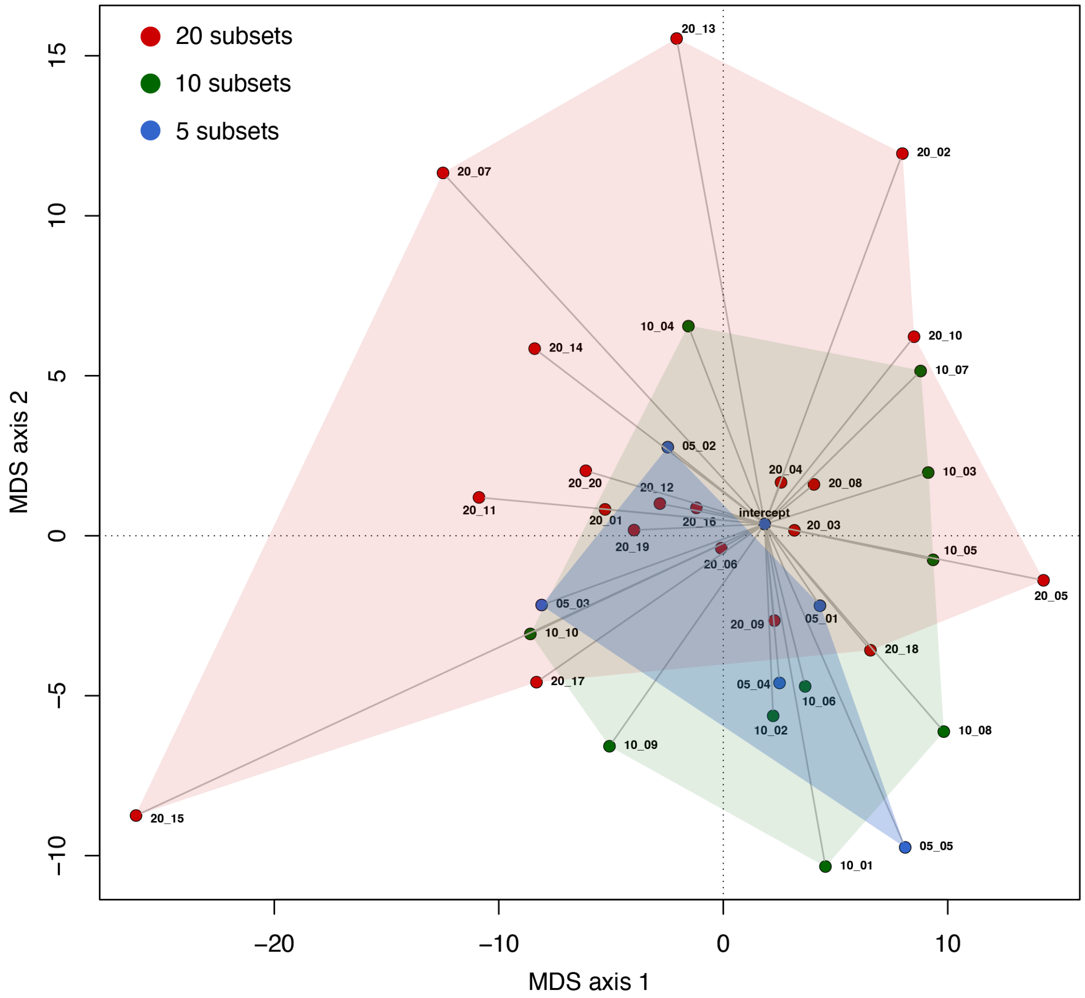

Supplementary Figure 8. Tree space plot for trees inferred in RAxML based on different subsets of the expanded matrix. This matrix was divided into 20 (50 loci x13 + 49 loci x7), 10 (99 loci x7 + 100 loci x3), and 5 (199 loci x3 + 198 loci x2) subsets. Colors represent the number of subsets. Labels next to the circles indicate the type of dataset and the subset number. The 20- and 10-gene subsets, which include fewer genes than the 5-gene subsets, are more dispersed in tree space (particularly the 20-gene subsets), suggesting a higher degree of sampling error.

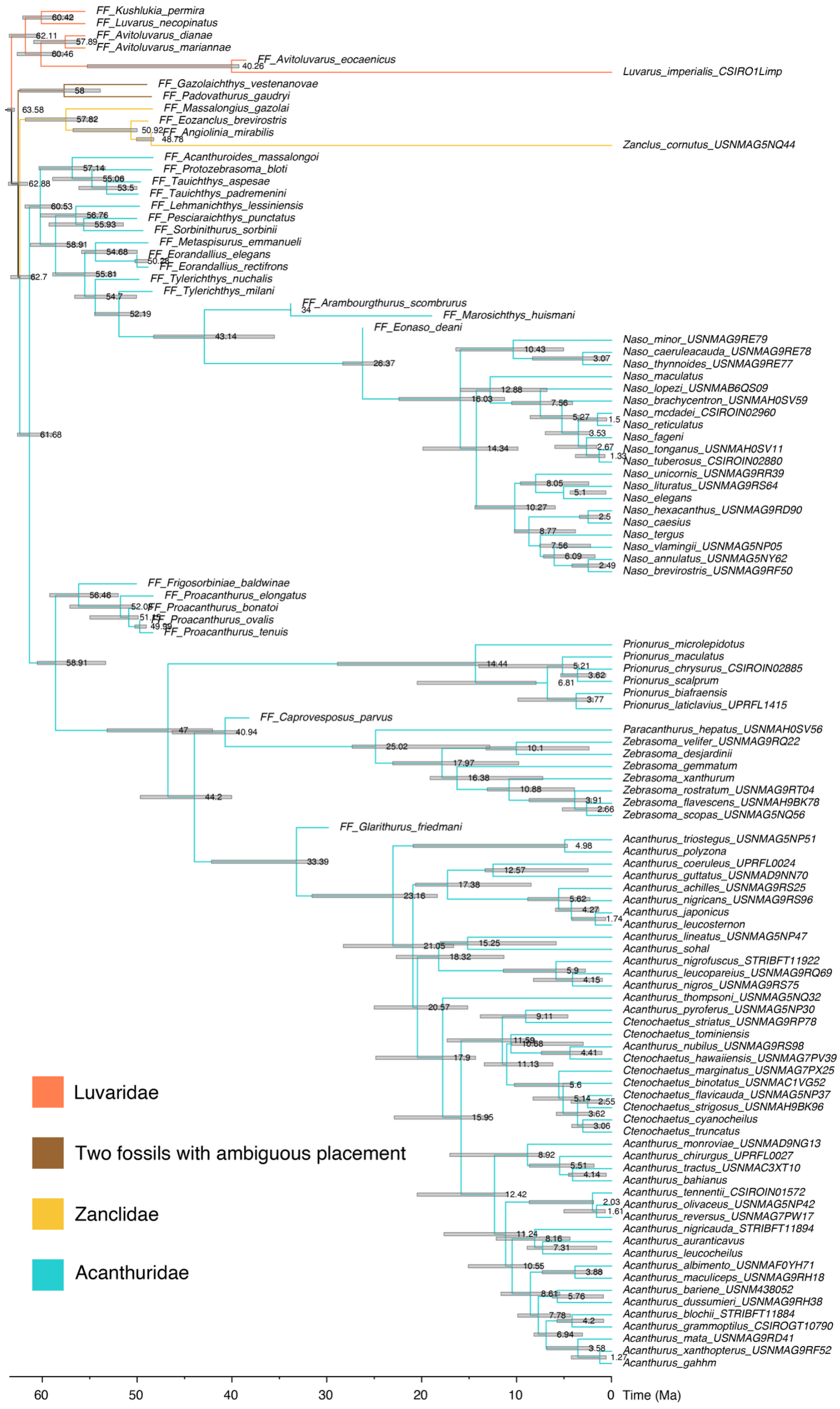

Supplementary Figure 9. Maximum clade credibility (MCC) tree of Acanthuriformes based on Scheme 1. Time-calibrated phylogenetic tree using a total-evidence framework based on Bayesian inference of 999 exons and 112 species (32 fossil, 80 extant). MCC tree generated from 10,000 trees evenly selected from the posterior distribution of five subsets. Colors indicate families. Posterior probability ages are given for each node. Ma: millions of years. Acanthuriforms

originated in the Paleocene at 63.6 Ma (95% highest posterior density [HPD]: 64–63.3 Ma), soon after the K-Pg mass extinction event, rather than the previously suggested 80 Ma based on more limited dataset that lacked a morphological matrix for fossil placement [35]. Luvars originated 62.1 Ma (95% HPD: 63.8–60.6 Ma), followed by unicornfishes, surgeonfishes and tangs (Acanthuridae) at 61.7 Ma (95% HPD: 62.9–59.3 Ma), and zanclids at 57.8 Ma (95% HPD: 62.1–54.6 Ma). At the genus level within acanthurids, most fossil genera originated between 60 and 50 Ma (e.g., *Avitoluvarus*, *Tauichthys*, and *Proacanthurus*), with *Naso* diversifying at 26.4 Ma (95% HPD: 28.5–23.5 Ma), *Prionurus* at 14.4 Ma (95% HPD: 29.1–12.2 Ma), *Paracanthurus* at 25.0 Ma (95% HPD: 27.5–12.9 Ma), *Zebrasoma* at 17.9 Ma (95% HPD: 23.2–9.8 Ma), *Acanthurus* at 23.2 Ma (95% HPD: 31.7–18.5 Ma), and the clade including *Acanthurus* + *Ctenochaetus* at 11.6 Ma (95% HPD: 17.4–8.2 Ma).

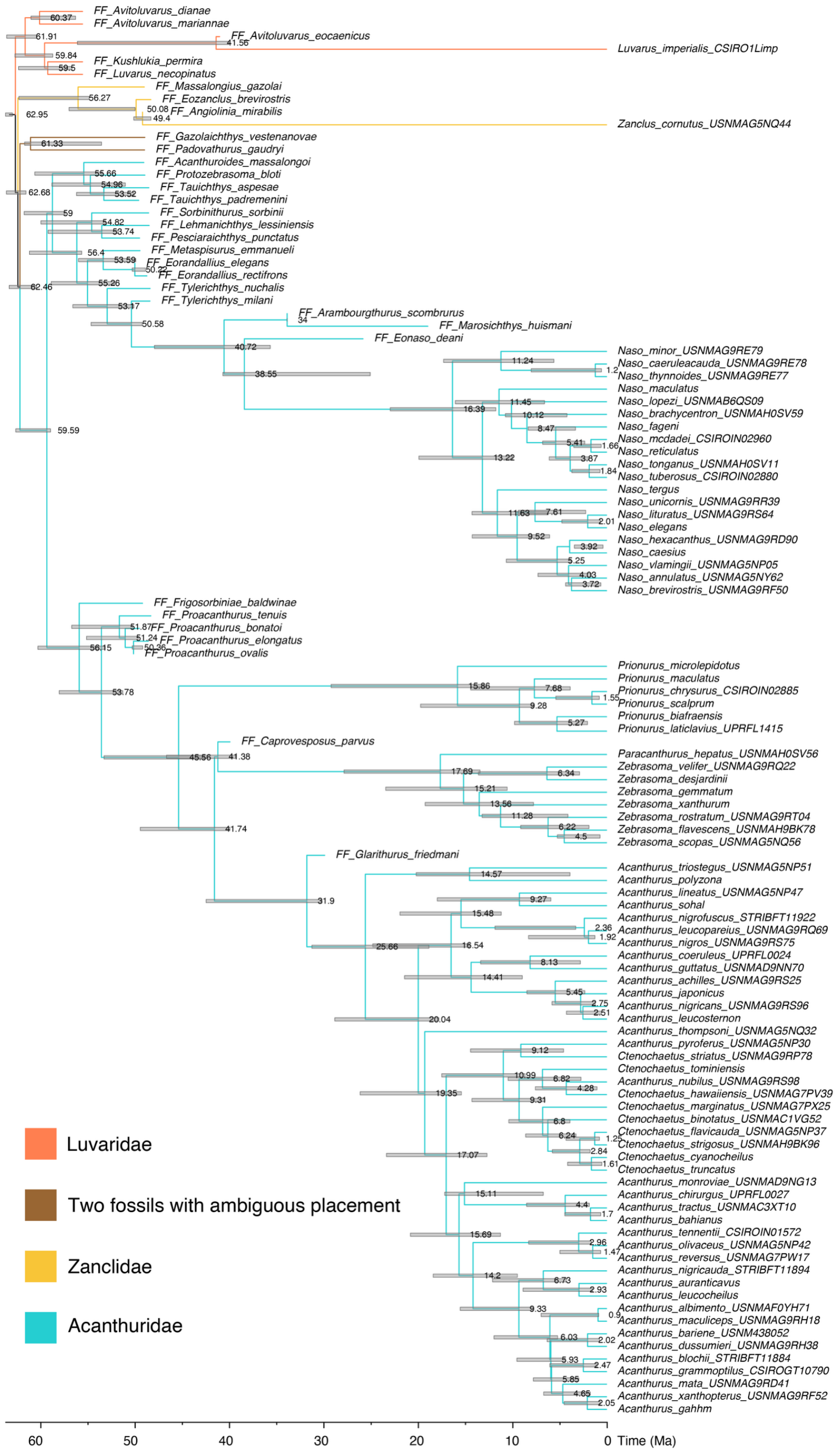

Supplementary Figure 10. Maximum clade credibility (MCC) tree of Acanthuriformes based on Scheme 2. Time-calibrated phylogenetic tree using a total-evidence framework based on Bayesian inference of 999 exons and 112 species (32 fossil, 80 extant). MCC tree generated from 10,000 trees evenly selected from the posterior distribution of five subsets. Colors indicate families. Posterior probability ages are given for each node. Ma: millions of years.

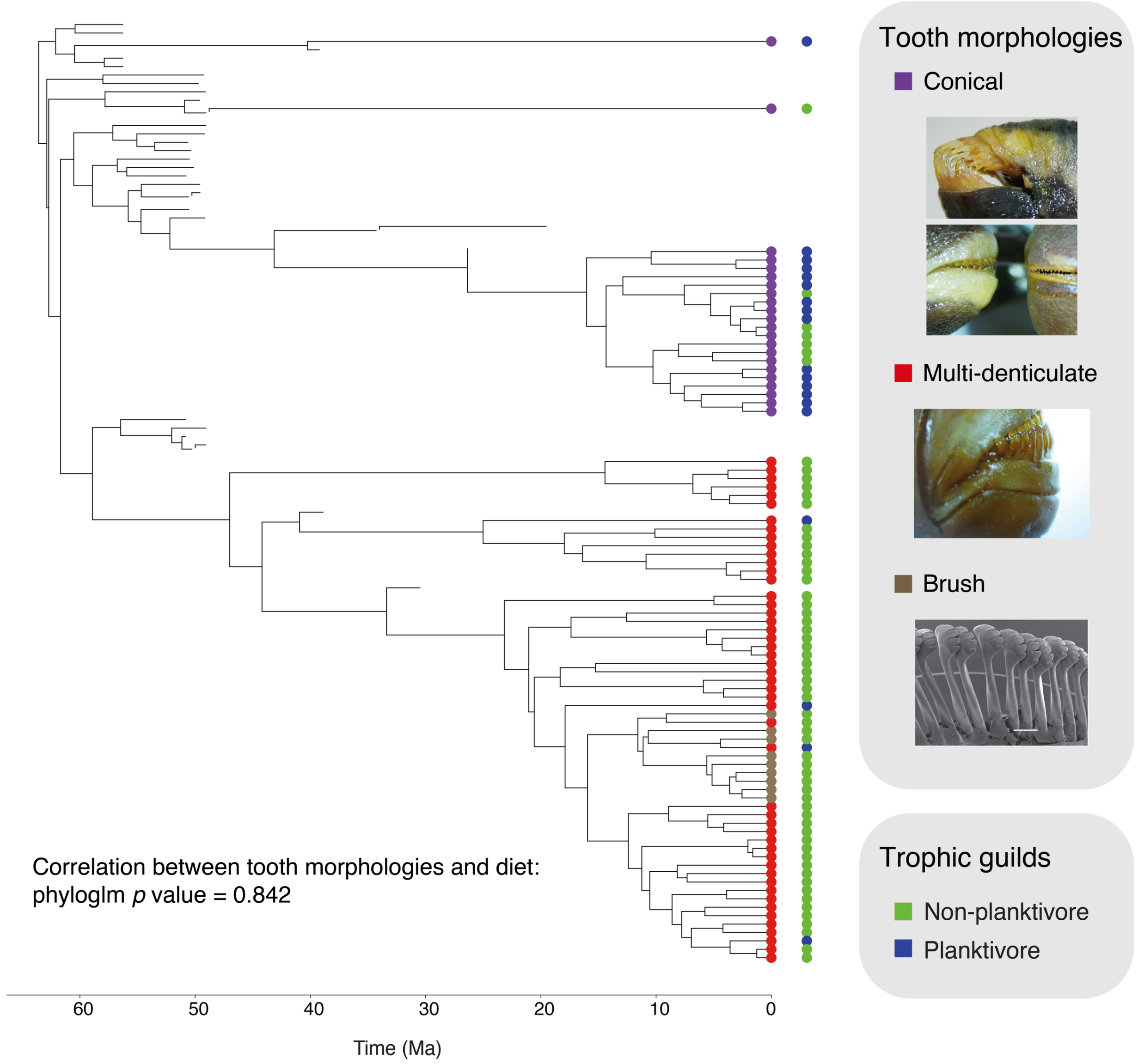

Supplementary Figure 11. Relationship between tooth morphology and diet in extant species only. The pies are color-coded according to different tooth morphologies and diets. Images depicting teeth are included for each tooth morphology category: conical (*Zanclus* and *Naso*), multi-denticulate (*Acanthurus*), and brush (*Ctenochaetus*). Most photographs were taken by A. Santaquiteria; the SEM image of the *Ctenochaetus* specimen, was sourced from [37]. Ma: millions of years.

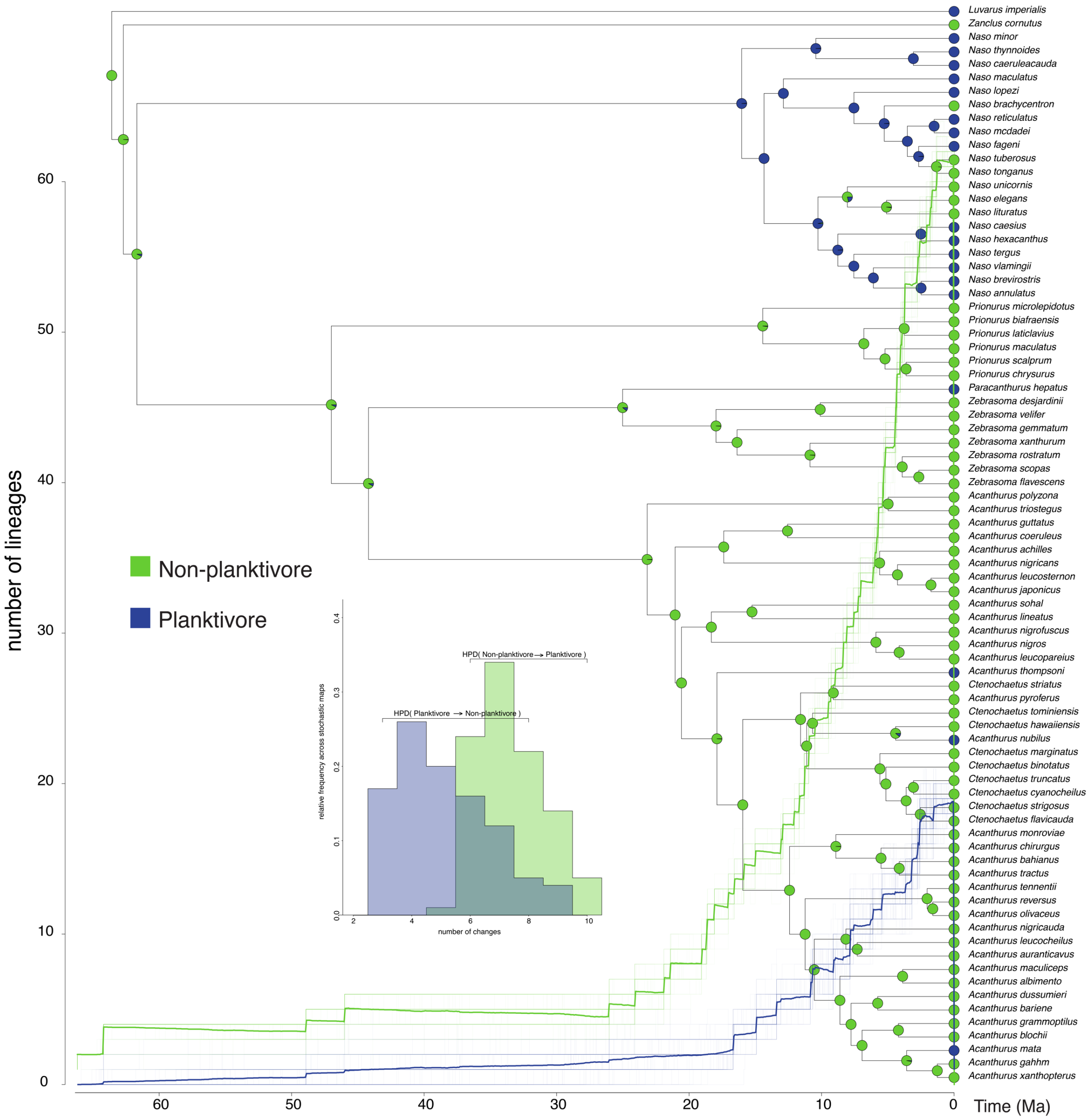

Supplementary Figure 12. Ancestral diet reconstruction of extant acanthuriforms based on MCC tree of Scheme 1. SIMMAP analyses based on the best-fit model, equal rates. The lineage through time (LTT) plot illustrates the number of non-planktivore and planktivore lineages over time (the line solid depicts the mean values from 100 simulations). Colors indicate each trophic guild. Ma: millions of years.

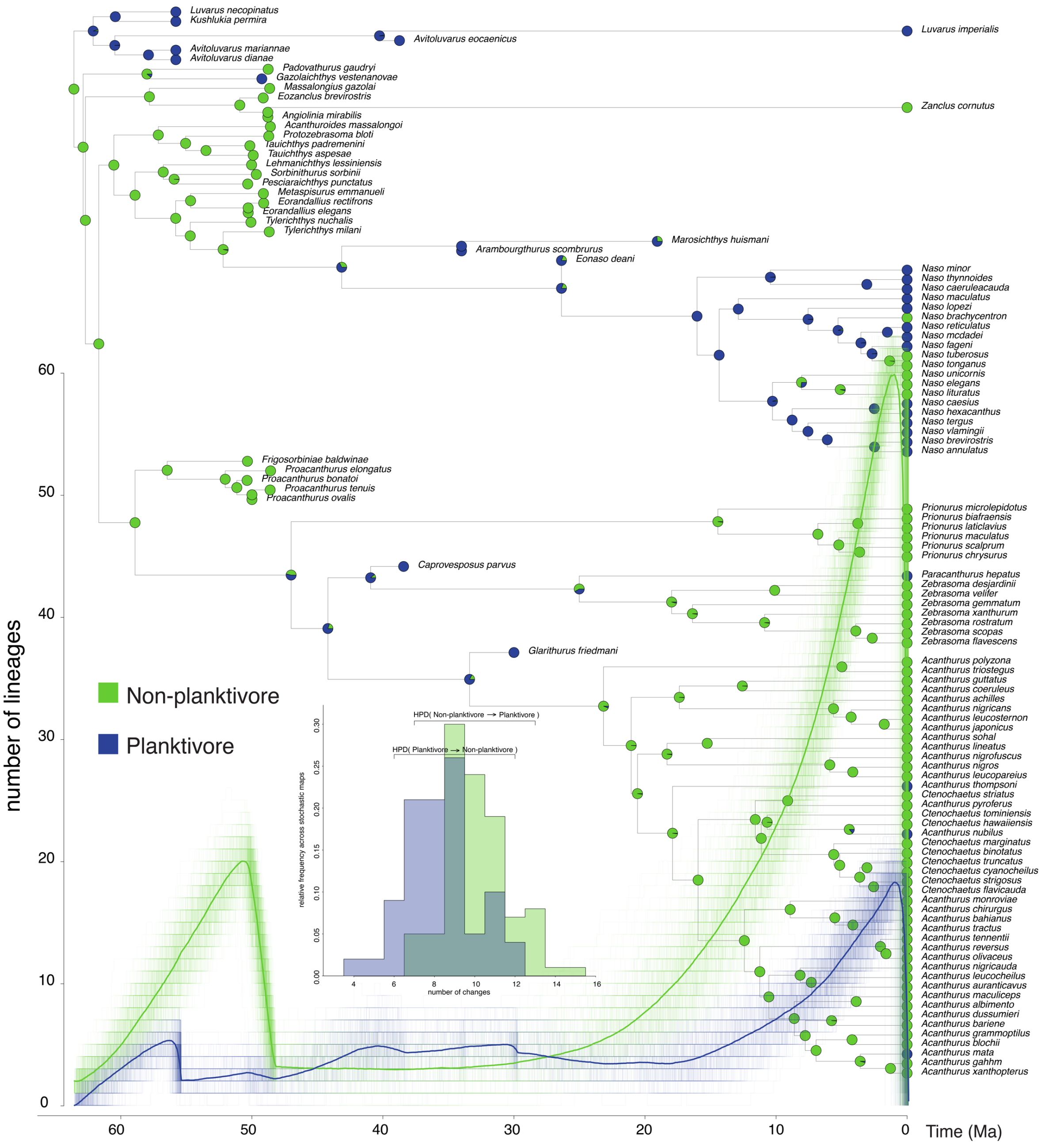

Supplementary Figure 13. Ancestral diet reconstruction of extant and fossil acanthuriforms based on MCC tree of Scheme 1. SIMMAP analyses based on the best-fit model, equal rates. The lineage through time (LTT) plot illustrates the number of non-planktivore and planktivore lineages across time (the solid line depicts the mean values from 500 trees). Colors indicate each trophic guild. Ma: millions of years.

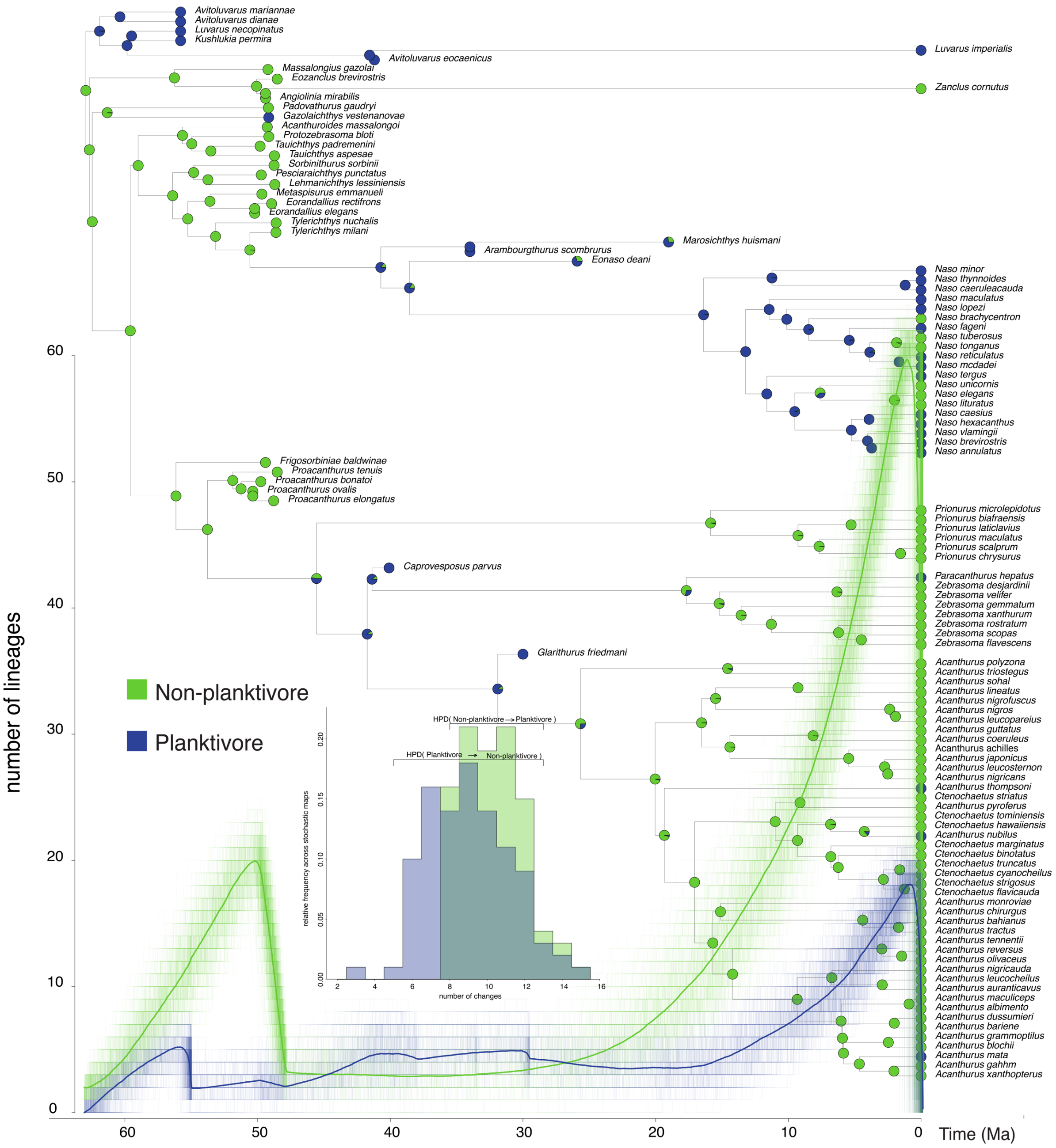

Supplementary Figure 14. Ancestral diet reconstruction of both extant and fossil acanthuriforms based on MCC tree of Scheme 2. SIMMAP analyses based on the best-fit model, equal rates. The lineage through time (LTT) plot illustrates the number of non-planktivore and planktivore lineages across time (the solid line depicts the mean values from 500 trees). Colors indicate each trophic guild. Ma: millions of years.

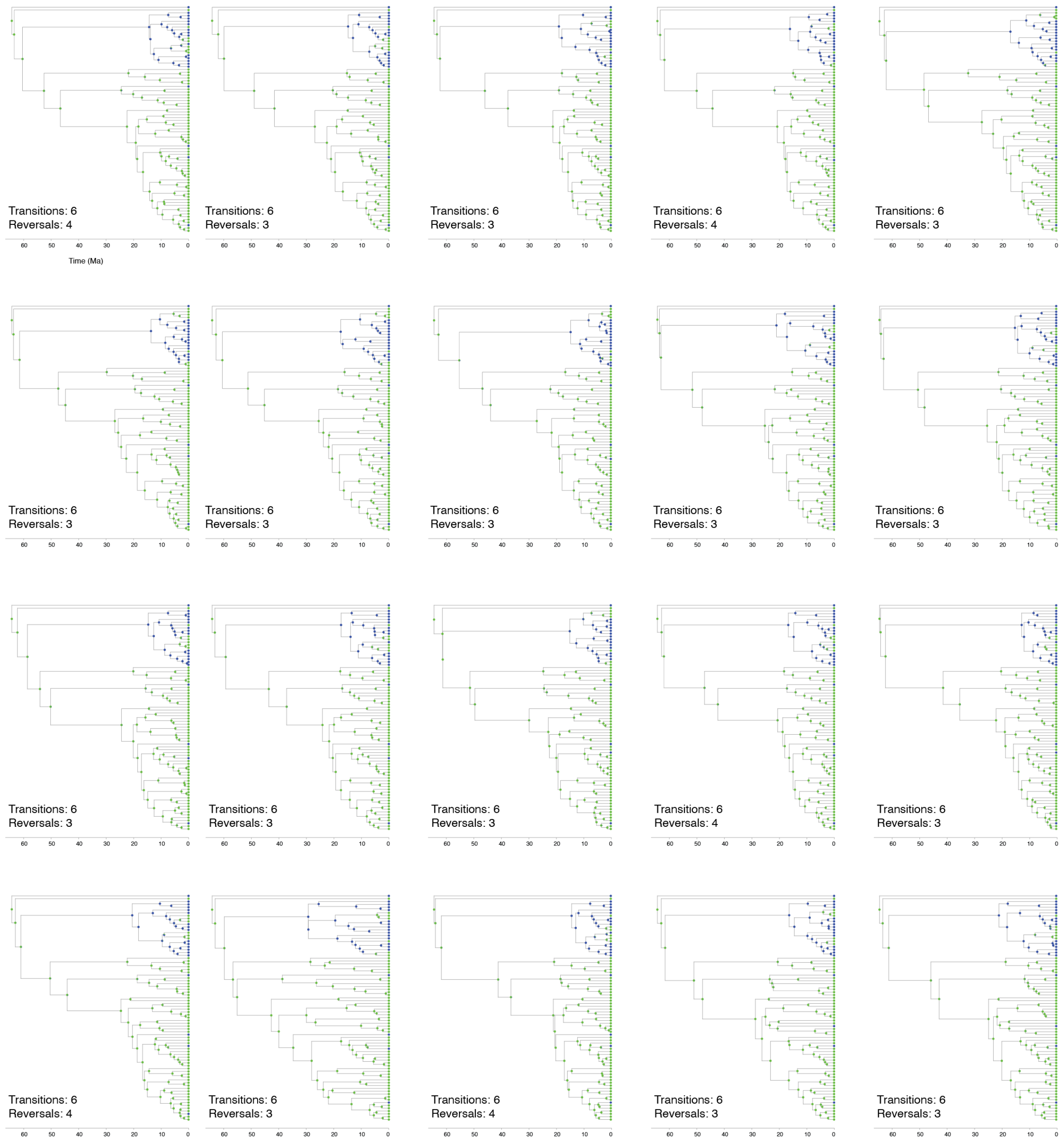

Supplementary Figure 15. Uncertainty in ancestral diet reconstruction analyses using extant species only. SIMMAP analyses based on the best-fit model with equal rates, conducted across 20 trees evenly sampled from the 500 trees in Scheme 1 (excluding fossils). Pie charts are color-coded by trophic guild: non-planktivores are shown in green, and planktivores in blue. At the bottom left of each panel, the number of transitions to planktivory and reversals to non-planktivory is indicated. Ma: millions of years.

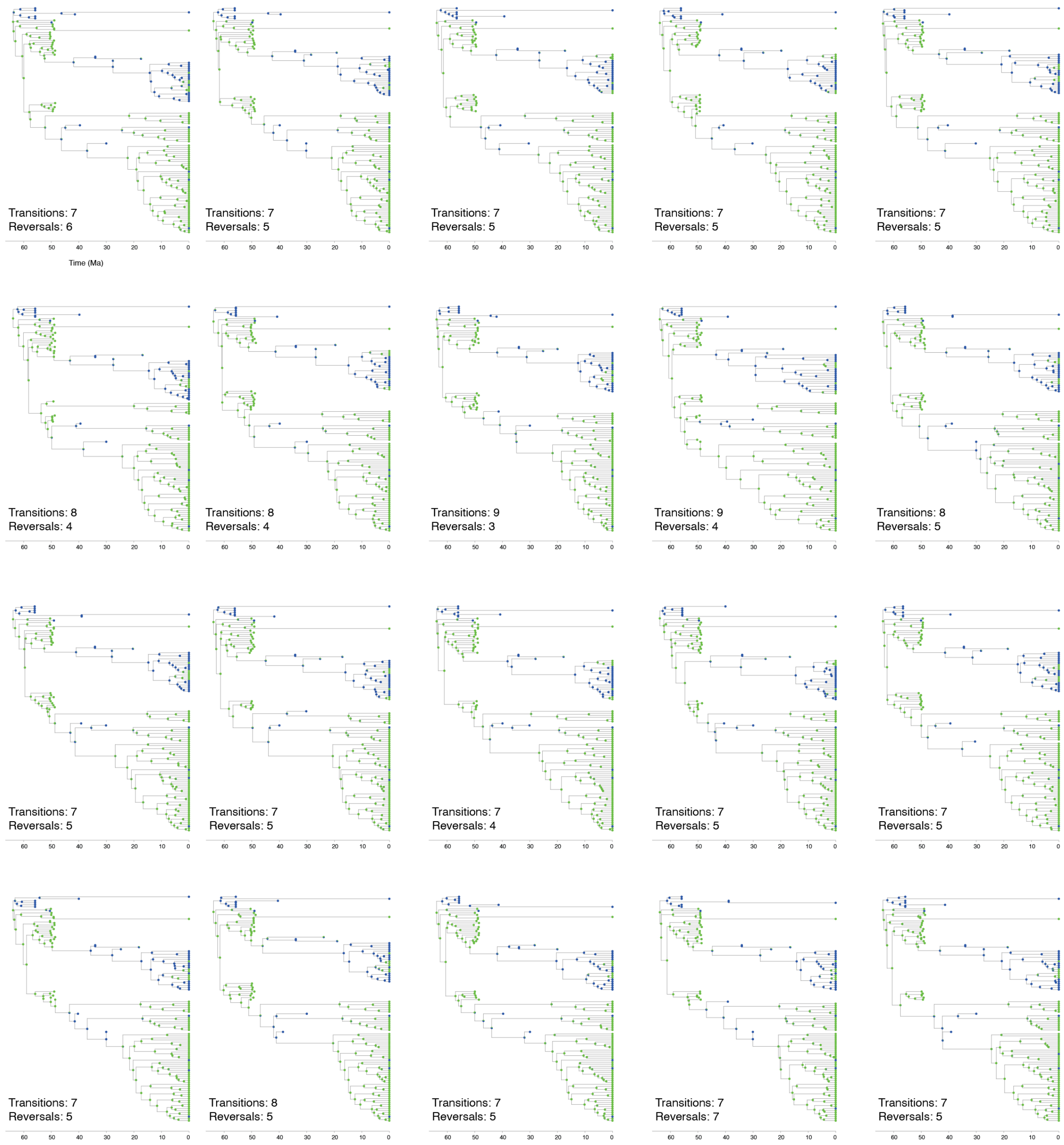

Supplementary Figure 16. Uncertainty in ancestral diet reconstruction analyses. SIMMAP analyses based on the best-fit model, equal rates for a total of 20 trees evenly sampled across the 500 trees from Scheme 1. Pies are color-coded based on trophic guild, with non-planktivores represented in green and planktivores in blue. At the bottom left of each panel, the number of transitions to planktivory and reversals to non-planktivory is indicated. Ma: millions of years.

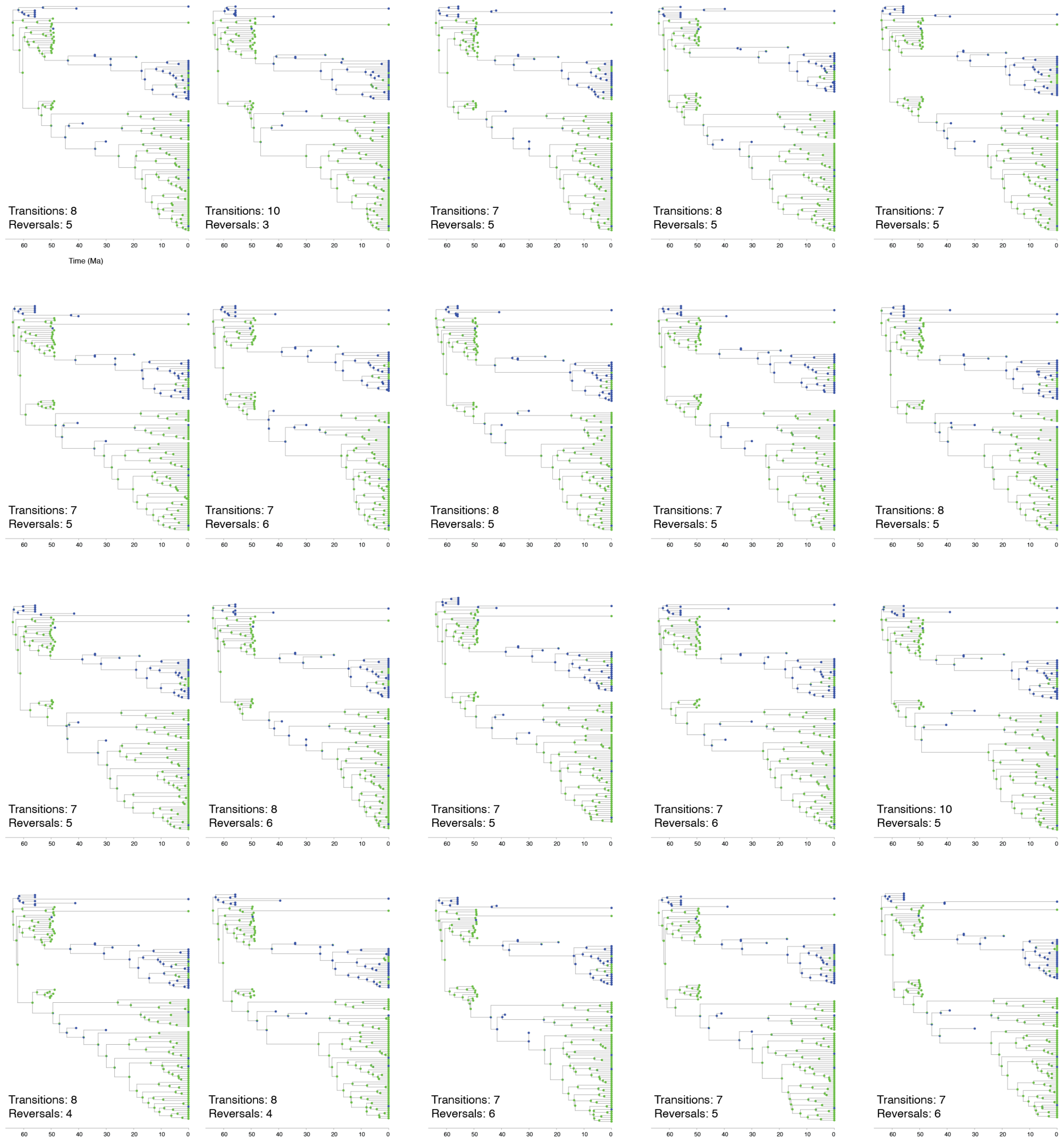

Supplementary Figure 17. Uncertainty in ancestral diet reconstruction analyses. SIMMAP analyses based on the best-fit model, equal rates for a total of 20 trees evenly sampled across the 500 trees from Scheme 2. Pies are color-coded based on trophic guild, with non-planktivores represented in green and planktivores in blue. At the bottom left of each panel, the number of transitions to planktivory and reversals to non-planktivory is indicated. Ma: millions of years.

Supplementary Table 5. Summary statistics of the 12 biogeographic models implemented in *BioGeoBEARS* based on the MCMC tree of Scheme 1 and 2. The best-fit model for each scheme is indicated in bold.

		Model	LnL	numparams	d	e	j	w	AICc	AICc weight
Scheme 1	*Tethys closure at 12Ma*	DEC	-304.2	2	0.047	0.011	0	1	612.4	1.10E-07
		DEC+J	-304.2	3	0.047	0.011	1.00E-05	1	614.5	3.80E-08
		DEC+W	-304.9	3	0.037	0.0087	0	0.27	616	1.90E-08
		DEC+J+W	-328.5	4	0.019	0.0074	1.00E-05	0.082	665.4	3.50E-19
		DIVALIKE	-319.4	2	0.052	0.011	0	1	642.9	2.70E-14
		DIVALIKE+J	-319.4	3	0.052	0.011	1.00E-05	1	645	9.30E-15
		DIVALIKE+W	-321.7	3	0.039	0.0083	0	0.23	649.7	9.10E-16
		DIVALIKE+J+W	-315.2	4	0.044	0.0077	1.00E-05	0.4	638.7	2.10E-13
		BAYAREALIKE	-294.7	2	0.023	0.038	0	1	593.4	0.0015
		BAYAREALIKE+J	-291	3	0.021	0.031	0.014	1	588.3	0.02
		**BAYAREALIKE+W**	**-287.5**	**3**	**0.015**	**0.033**	**0**	**0.25**	**581.3**	**0.64**
		BAYAREALIKE+J+W	-287.1	4	0.0075	0.026	0.0063	0.086	582.6	0.34
	Tethys closure at 16Ma	DEC	-302.7	2	0.049	0.0097	0	1	609.5	1.60E-07
		DEC+J	-302.7	3	0.049	0.0097	1.00E-05	1	611.6	5.50E-08
		DEC+W	-311.8	3	0.027	0.0043	0	0.17	629.8	6.30E-12
		DEC+J+W	-300.7	4	0.038	0.0098	1.00E-05	0.3	609.8	1.30E-07
		DIVALIKE	-318.1	2	0.055	0.0094	0	1	640.4	3.10E-14
		DIVALIKE+J	-318.1	3	0.055	0.0094	1.00E-05	1	642.5	1.10E-14
		DIVALIKE+W	-349.2	3	0.021	0.0033	0	0.056	704.5	3.70E-28
		DIVALIKE+J+W	-312.7	4	0.045	0.0072	1.00E-05	0.37	633.7	8.70E-13
		BAYAREALIKE	-292.8	2	0.025	0.038	0	1	589.7	0.0032
		BAYAREALIKE+J	-289.6	3	0.022	0.032	0.013	1	585.4	0.027
		**BAYAREALIKE+W**	**-286**	**3**	**0.017**	**0.033**	**0**	**0.3**	**578.3**	**0.94**
		BAYAREALIKE+J+W	-288.5	4	0.0067	0.025	0.0083	0.058	585.5	0.026
Scheme 2	*Tethys closure at 12Ma*	DEC	-305.5	2	0.049	0.0098	0	1	615.1	2.00E-10
		DEC+J	-305.5	3	0.049	0.0098	1.00E-05	1	617.2	6.80E-11
		DEC+W	-306.2	3	0.037	0.0089	0	0.28	618.5	3.50E-11
		DEC+J+W	-306.8	4	0.036	0.0081	1.00E-05	0.27	622.1	6.00E-12
		DIVALIKE	-322.6	2	0.054	0.0098	0	1	649.2	7.60E-18
		DIVALIKE+J	-322.5	3	0.056	0.0095	1.00E-05	1	651.3	2.70E-18
		DIVALIKE+W	-326	3	0.037	0.006	0	0.23	658.3	8.20E-20
		DIVALIKE+J+W	-318.8	4	0.044	0.0064	1.00E-05	0.37	646	3.70E-17
		BAYAREALIKE	-288.1	2	0.025	0.042	0	1	580.3	0.007
		BAYAREALIKE+J	-285.4	3	0.022	0.034	0.012	1	577.1	0.035
		**BAYAREALIKE+W**	**-282.1**	**3**	**0.018**	**0.036**	**0**	**0.31**	**570.5**	**0.95**
		BAYAREALIKE+J+W	-286.7	4	0.0051	0.025	0.0054	0.036	581.7	0.0034
	Tethys closure at 16Ma	DEC	-303.1	2	0.051	0.0092	0	1	610.3	4.30E-10
		DEC+J	-303.1	3	0.051	0.0092	1.00E-05	1	612.5	1.50E-10
		DEC+W	-298.6	3	0.046	0.0076	0	0.43	603.5	1.30E-08
		DEC+J+W	-298.6	4	0.044	0.0076	1.00E-05	0.43	605.5	4.90E-09
		DIVALIKE	-320.7	2	0.065	0.0094	0	1	645.5	1.00E-17
		DIVALIKE+J	-320.1	3	0.058	0.0081	1.00E-05	1	646.4	6.30E-18
		DIVALIKE+W	-315.5	3	0.051	0.0075	0	0.39	637.2	6.40E-16
		DIVALIKE+J+W	-337.6	4	0.028	0.0063	1.00E-05	0.13	683.6	5.40E-26
		BAYAREALIKE	-286.2	2	0.026	0.042	0	1	576.5	0.0099
		BAYAREALIKE+J	-283.8	3	0.023	0.033	0.013	1	573.8	0.038
		**BAYAREALIKE+W**	**-280.5**	**3**	**0.019**	**0.036**	**0**	**0.31**	**567.3**	**0.95**
		BAYAREALIKE+J+W	-287	4	0.0048	0.027	0.0057	0.028	582.4	0.0005

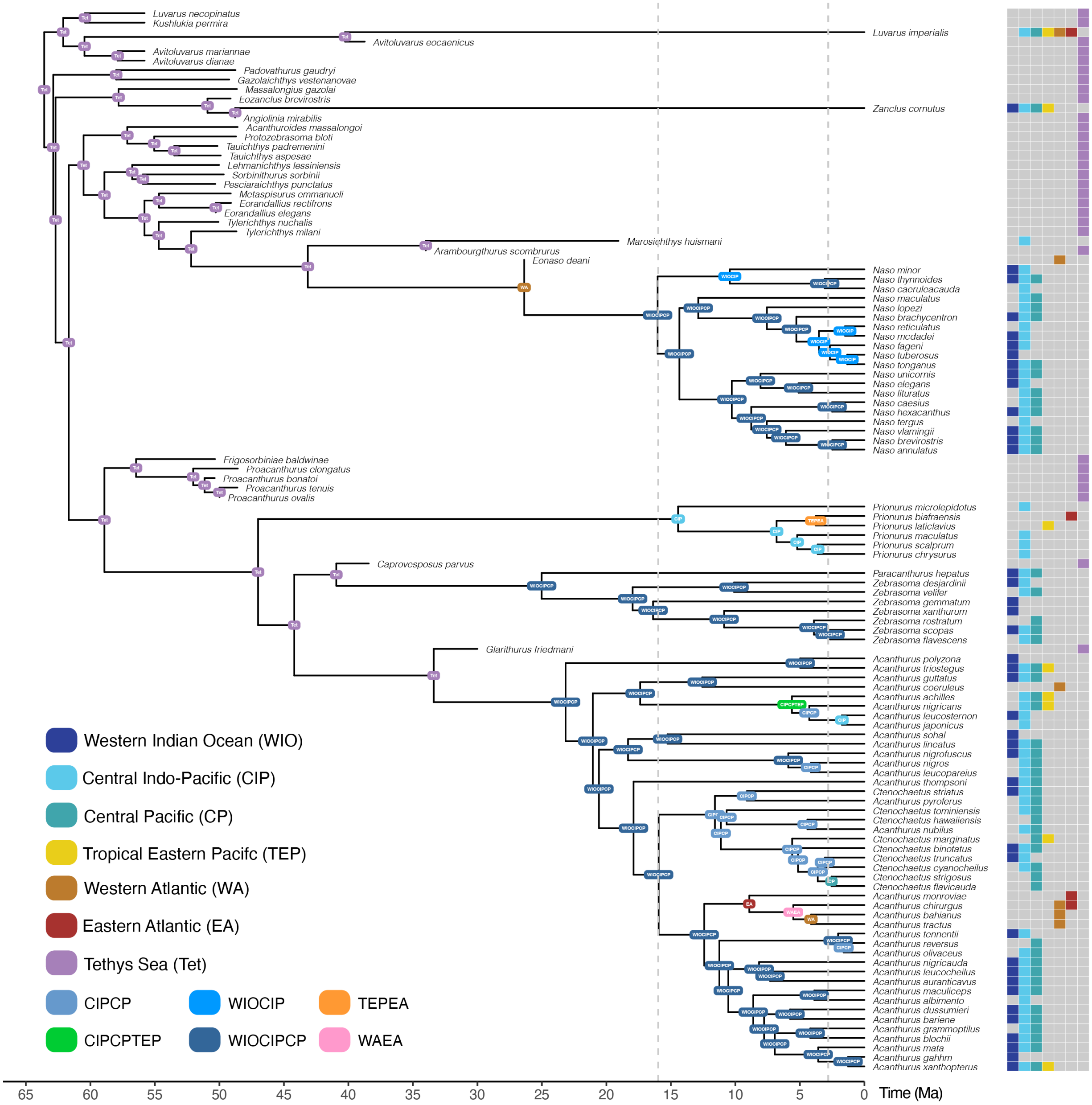

Supplementary Figure 18. Ancestral range estimations for Acanthuriformes using the best-supported biogeographic model (BAYAREALIKE+w) applied to 20 trees subsampled across the five subsets from Scheme 1 and using the MCC tree of Scheme 1 as a fixed topology in *BioGeoBEARS*. Note that this analysis assumes that the final closure of the Tethys Seaway occurred at 16 Ma. Boxes at nodes and tips are color-coded by area, or areas with the highest ML probability. Ma: millions of years.

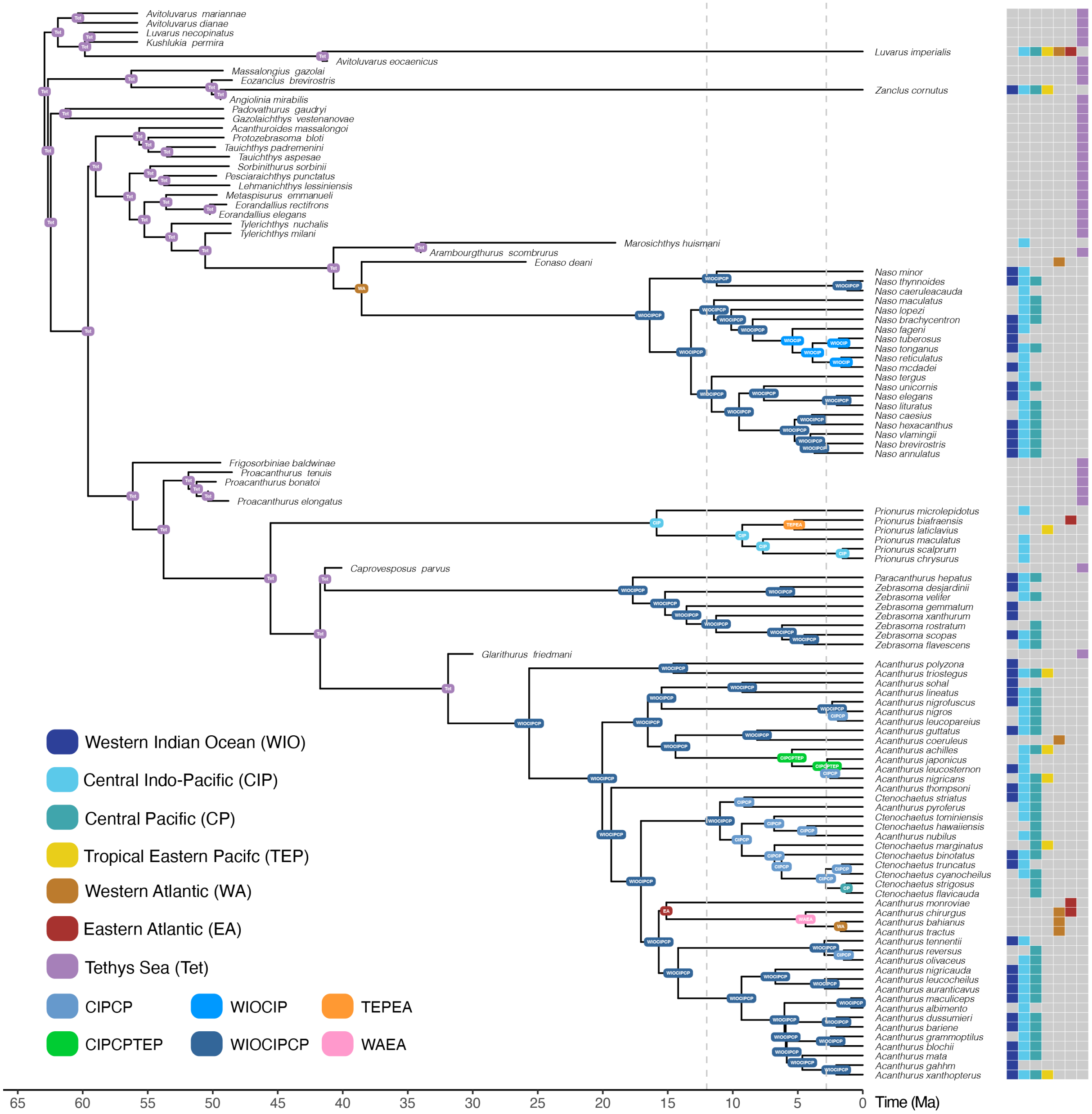

Supplementary Figure 19. Ancestral range estimations for Acanthuriformes using the best-supported biogeographic model (BAYAREALIKE+w) applied to 20 trees subsampled across the five subsets from Scheme 2 and using the MCC tree of Scheme 2 as a fixed topology in *BioGeoBEARS*. Note that this analysis assumes that the final closure of the Tethys Seaway occurred at 12 Ma. Boxes at nodes and tips are color-coded by area, or areas with the highest ML probability. Ma: millions of years.

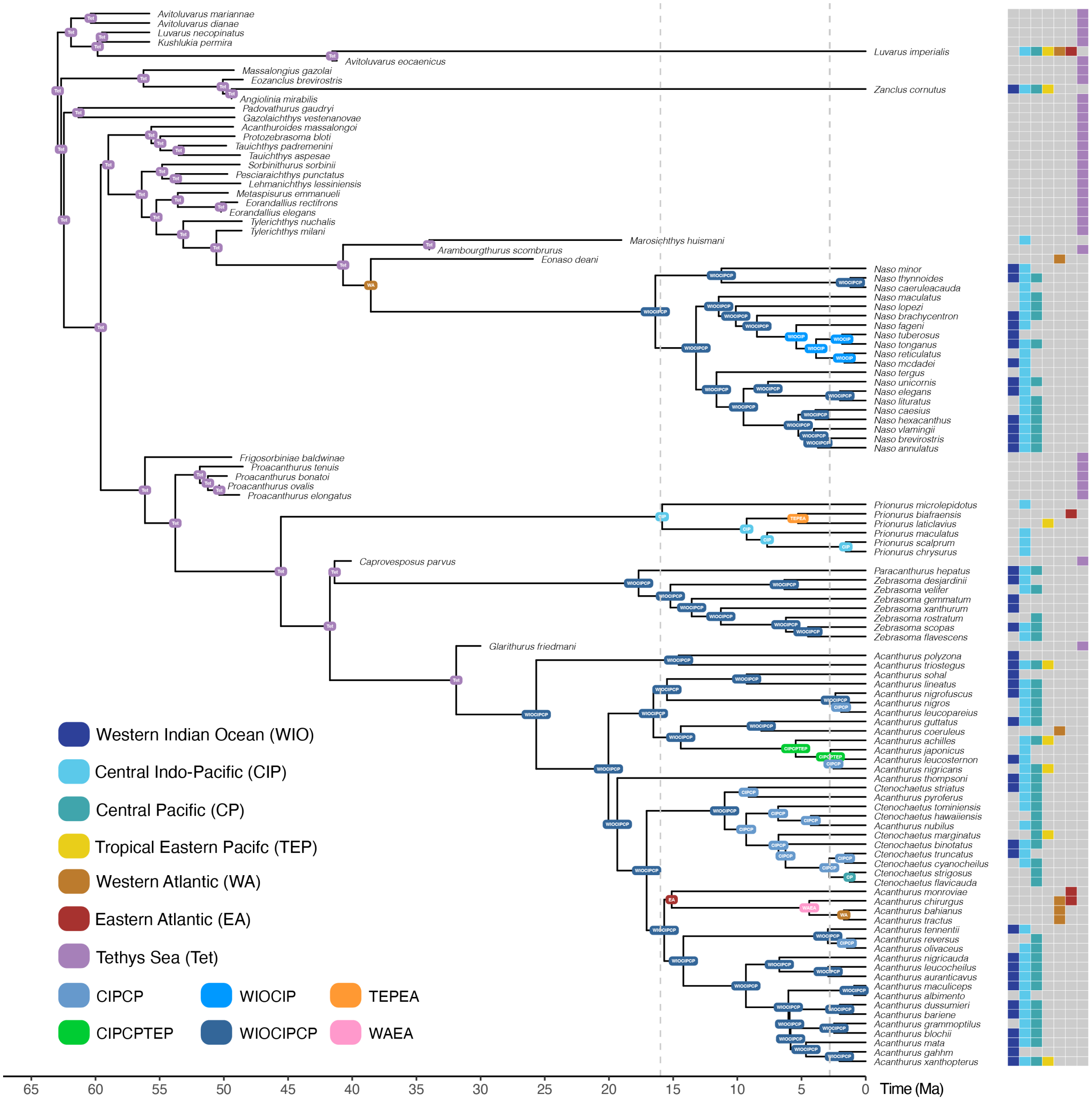

Supplementary Figure 20. Ancestral range estimations for Acanthuriformes using the best-supported biogeographic model (BAYAREALIKE+w) applied to 20 trees subsampled across the five subsets from Scheme 2 and using the MCC tree of Scheme 2 as a fixed topology in *BioGeoBEARS*. Note that this analysis assumes that the final closure of the Tethys Seaway occurred at 16 Ma. Boxes at nodes and tips are color-coded by area, or areas with the highest ML probability. Ma: millions of years.

Supplementary Table 6. HiSSE alternative models of lineage diversification and model fitting results trophic guilds based on the MCC tree of each scheme and excluding fossils.

	Model	Number of hidden states	lnL	AIC	AICc	AIC weight
**Scheme 1**	Null BiSSE	0	-303.033	614.066	614.599	0.015
	Full BiSSE	0	-302.995	617.991	619.141	0.002
	Full HiSSE	2	-293.419	612.838	618.353	0.028
	CID-2	2	-295.954	605.908	607.463	0.910
	CID-4	4	-294.990	611.980	615.862	0.044
**Scheme 2**	Null BiSSE	0	-298.158	604.316	604.850	0.053
	Full BiSSE	0	-297.905	607.810	608.961	0.009
	Full HiSSE	2	-289.339	604.677	610.192	0.044
	CID-2	2	-292.380	598.760	600.316	0.845
	CID-4	4	-291.212	604.423	608.306	0.050

lnL = log likelihood; AIC = Akaike Information Criterion.

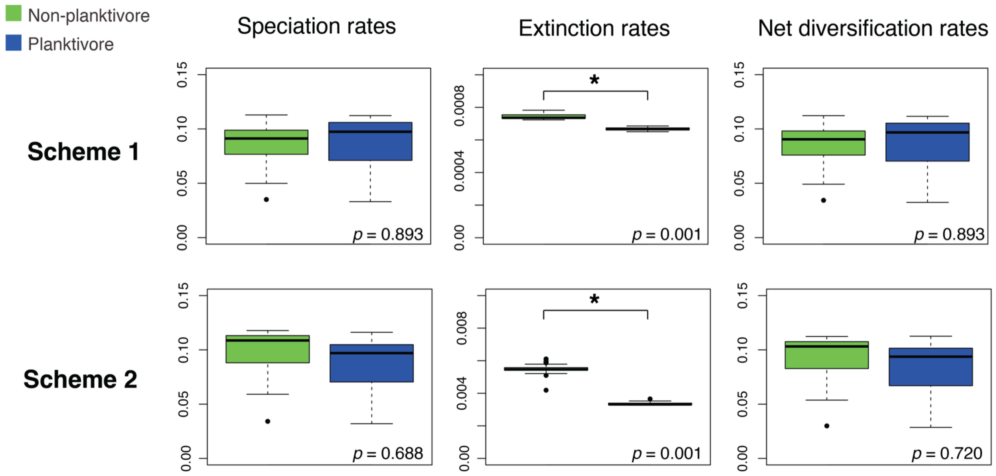

Supplementary Figure 21. Box plots of model averaged tip rates estimated with HiSSE for speciation, extinction, and net diversification rates in extant non-planktivore and planktivore lineages. Phylogenetic ANOVA significance values are also shown at the bottom right of each panel. First row of panels shows the results obtained based on the MCC tree of Scheme 1 and the second row based on the MCC tree of Scheme 2, both excluding fossils. Box plots indicate the median (middle line), the 25th and 75th percentiles (box), the 5th and 95th percentiles (whiskers), as well as outliers (individual points). * *p*-value < 0.05.

Supplementary Table 7. HiSSE results across the MCC tree and the 20 trees subsampled across the five subsets from Schemes 1 and 2. Transition rates between the two trophic guilds, non-planktivore and planktivore, are displayed for each tree. Statistical results estimating the relationships between diet and the different tip-associated rates (diversification, speciation, and extinction) are illustrated with *p*-values calculated for each tree using two-sided phylogenetic ANOVA . Significant *p*-values are highlighted in red.

		Transition rates		p-values between rates and diet		
	Tree	from non-planktivore to planktivore	from planktivore to non-planktivore	Diversication rates	Speciation rate	Extinction rate
**Scheme 1**	MCC	0.010	0.026	0.893	0.893	0.001
	1	0.011	0.032	0.954	0.803	0.001
	2	0.010	0.030	0.982	0.929	0.001
	3	0.010	0.025	0.837	0.799	0.001
	4	0.011	0.033	0.949	0.838	0.001
	5	0.010	0.032	0.933	0.910	0.001
	6	0.009	0.033	0.402	0.527	0.424
	7	0.010	0.026	0.900	0.489	0.001
	8	0.010	0.034	0.951	0.972	0.731
	9	0.010	0.026	0.746	0.745	0.757
	10	0.010	0.031	0.995	0.964	0.001
	11	0.010	0.031	0.981	0.996	0.001
	12	0.009	0.023	0.703	0.700	0.001
	13	0.009	0.032	0.767	0.856	0.602
	14	0.011	0.032	0.886	0.832	0.001
	15	0.010	0.028	0.933	0.906	0.530
	16	0.010	0.027	0.894	0.884	0.001
	17	0.007	0.013	0.001	0.001	0.001
	18	0.011	0.034	0.983	0.887	0.001
	19	0.009	0.029	0.878	0.926	0.893
	20	0.010	0.028	0.963	0.962	0.895
**Scheme 2**	MCC	0.010	0.026	0.772	0.688	0.001
	1	0.011	0.030	0.808	0.836	0.001
	2	0.010	0.022	0.878	0.885	0.001
	3	0.009	0.025	0.927	0.916	0.898
	4	0.009	0.032	0.993	0.809	0.158
	5	0.009	0.022	0.828	0.833	0.001
	6	0.009	0.022	0.893	0.800	0.001
	7	0.011	0.024	0.757	0.707	0.052
	8	0.011	0.030	0.925	0.928	0.968
	9	0.008	0.018	0.704	0.683	0.001
	10	0.009	0.022	0.848	0.857	0.613
	11	0.009	0.024	0.903	0.778	0.518
	12	0.011	0.028	0.890	0.821	0.006
	13	0.009	0.022	0.733	0.714	0.001
	14	0.010	0.029	0.897	0.832	0.001
	15	0.010	0.035	0.984	0.988	0.001
	16	0.008	0.020	0.800	0.741	0.001
	17	0.009	0.020	0.706	0.647	0.001
	18	0.010	0.030	0.874	0.955	0.001
	19	0.009	0.025	0.992	0.913	0.001
	20	0.011	0.037	0.955	0.958	0.487

Supplementary Table 8. Climatic independent and dependent model results. AIC scores and AIC weights for models testing the correlation between trophic shifts and global average or tropical paleo-climatic curves. Analyses were performed using both the MCC tree and the 500 posterior trees from each scheme. The best-fitting model for each scheme is shown in bold.

		Temperature Curve	Clim fit AIC	BM fit AIC	EB fit AIC	Lambda fit AIC	sigma	beta	Clim fit AICw	BM fit AICw	EB Fit AICw	Lambda Fit AICw
**Scheme 1**	*MCC tree*	Scotese Global Avg.	222.948	231.953	233.953	222.486	1.000	2.987	**0.440**	0.005	0.002	**0.554**
		Scotese Tropical	222.590	231.953	233.953	222.486	1.000	2.940	**0.484**	0.004	0.002	**0.510**
	500 trees	Scotese Global Avg.	257.766	263.505	265.503	256.533	1.000	-0.094	**0.340**	0.019	0.007	**0.633**
		Scotese Tropical	259.961	263.505	265.503	256.533	1.000	-0.094	0.147	0.025	0.009	**0.818**
**Scheme 2**	*MCC tree*	Scotese Global Avg.	223.334	233.116	235.116	222.495	1.000	0.000	**0.395**	0.003	0.001	**0.601**
		Scotese Tropical	224.920	233.116	235.116	222.495	1.000	0.000	0.228	0.004	0.001	**0.767**
	500 trees	Scotese Global Avg.	256.964	262.390	264.389	255.716	1.000	-0.088	**0.339**	0.022	0.008	**0.630**
		Scotese Tropical	258.997	262.390	264.389	255.716	1.000	-0.088	0.156	0.029	0.011	**0.805**

BM = Brownian motion; EB = Early burst; Clim= Climatic model; AIC = Akaike Information Criterion; AICw = AIC weight.

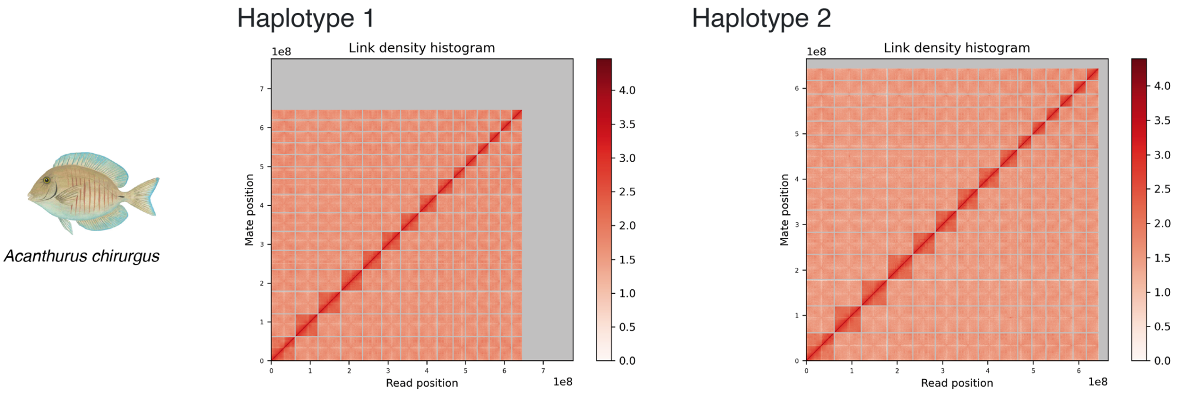

Assembly	Total Length (bp)	N50	L50	N90	L90	Total Length (bp)	N50	L50	N90	L90
**Input Assembly**	776,875,734	23,491,650	12	35,706	1,224	664,955,783	8,334,248	10	8,334,248	25
**Dovetail HiRise Assembly**	776,879,634	47,485,454	8	35,706	1,185	664,959,085	29,137,030	7	29,137,030	14

Supplementary Figure 22. Overview results from each haplotype of the *Acanthurus chirurgus* chromosome-level genome. Link-density histograms visualize how scaffolds interact with each other across the entire genome for each haplotype. The table represents the scaffold genome assembly statistics for each haplotype from PacBio and Hi-C sequencing. The Dovetail HiRise assembly resulted in 15 putative chromosomes.

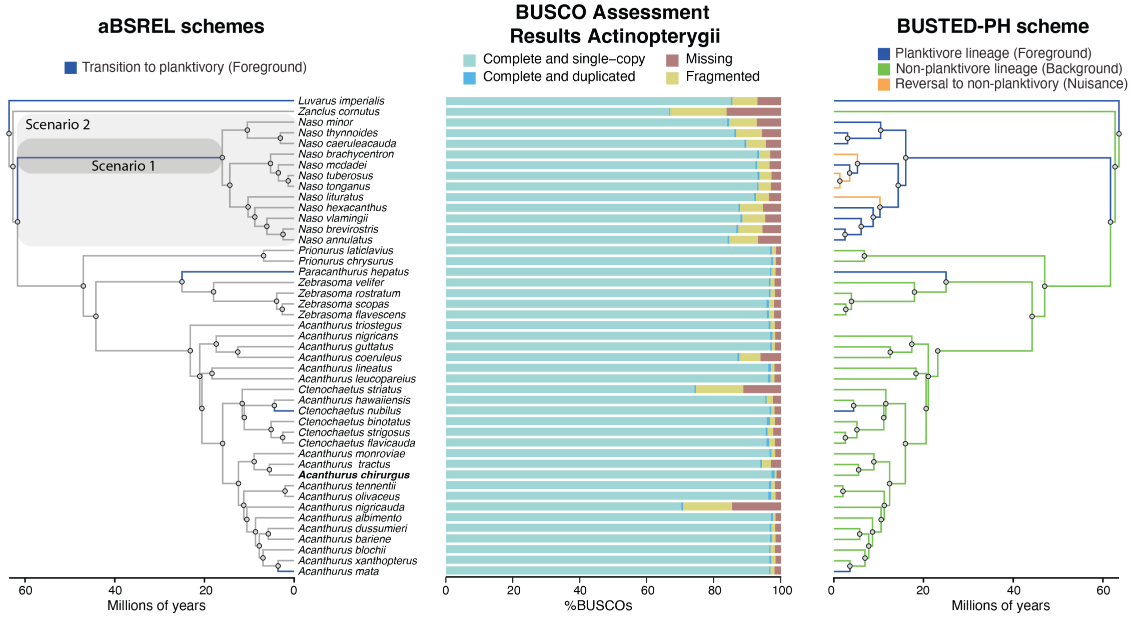

Supplementary Figure 23. Schematic representation of the two aBSREL scenarios and BUSTED-PH analyses used to identify positively selected genes (PSGs) in HyPhy. aBSREL was applied to branches associated with transitions to planktivory and to lineages exhibiting a planktivorous diet. The middle panel shows genome completeness based on BUSCO scores (using Actinopterygii odb12) for all species, with the chromosome-level assembly of the *Acanthurus chirurgus* highlighted in bold. Short-read assemblies are shown for the remaining species.

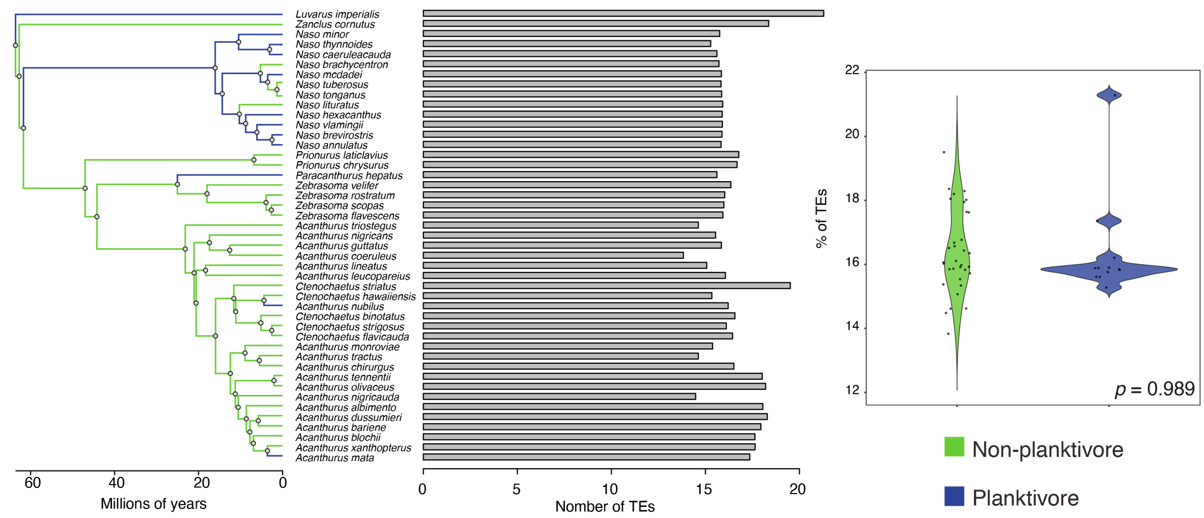

Supplementary Figure 24. Correlation between the number of transportable elements (TEs) and diet. The phylogenetic tree illustrates the ancestral diet reconstruction for species with whole-genome data. Horizontal gray bars represent the number of TEs per species. Violin plots depict the distribution of the % of TEs across non-planktivore and planktivore lineages, with dots representing the raw scores for each species. The significance value from the phylogenetic ANOVA is shown at the bottom right of the violin plot.

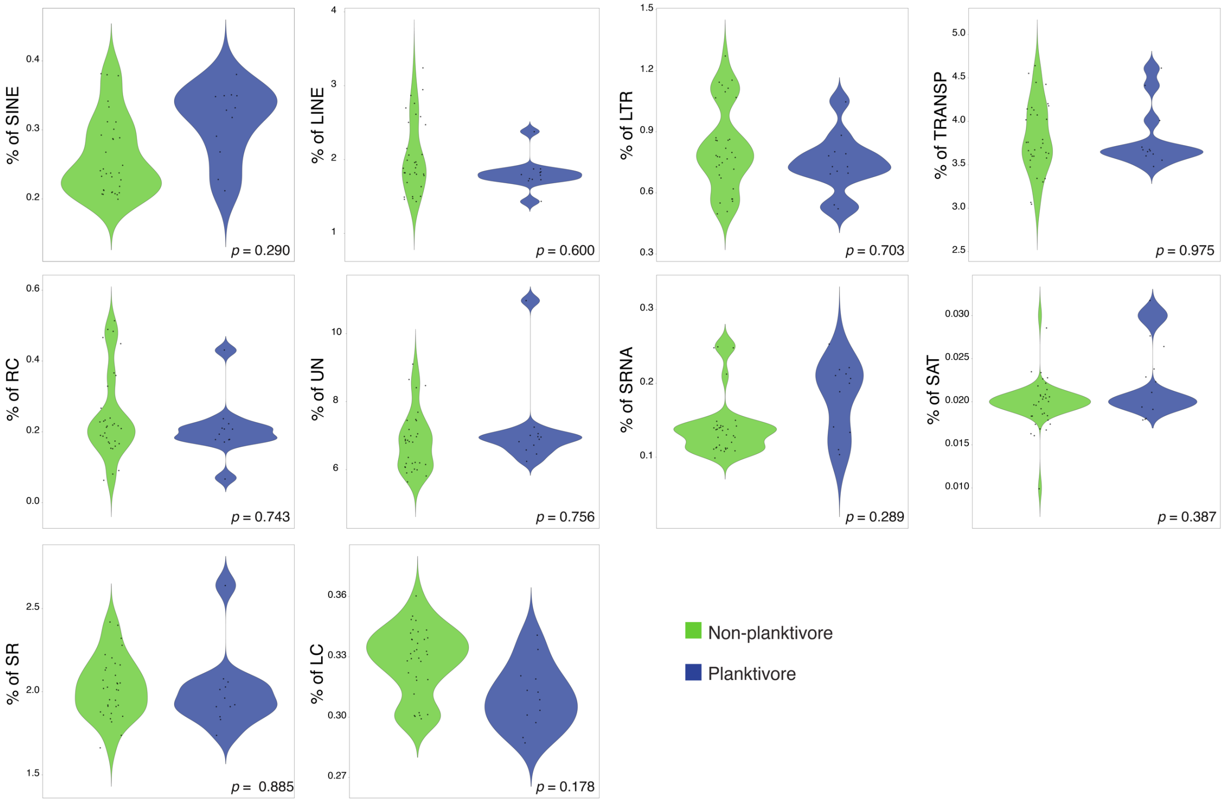

Supplementary Figure 25. Correlation between the different repetitive element categories with each trophic guild. Violin plots depict the distribution of the percentage of each category across non-planktivorous and planktivorous lineages, with dots representing the raw scores for each species. Phylogenetic ANOVA significance values are shown at the bottom right of each panel.

Supplementary References

1. Randall, J. E. A contribution to the biology of the Acanthuridae (surgeon fishes). Honolulu: University of Hawaii (1955).
2. Tyler, J. C. Osteological Aspects of Interrelationships of Surgeon Fish Genera (Acanthuridae). *Proceedings of the Academy of Natural Sciences of Philadelphia* **122**, 87–124 (1970).
3. Tyler, J. C. & Micklich, N. R. A new genus and species of surgeon fish (Perciformes, Acanthuridae) from the Oligocene of Kanton Glarus, Switzerland. *Swiss Journal of Palaeontology* **130**, 203–216 (2011).
4. Bannikov, A. F. & Tyler, J. C. Phylogenetic revision of the fish families Luvaridae and Kushlukidae (Acanthuroidei), with a new genus and two new species of Eocene luvarids. *Smithsonian Contributions to Paleobiology* **81**, 1–45 (1995).
5. Winterbottom, R. & McLennan, D. A. Cladogram versatility: Evolution and biogeography of acanthuroid fishes. *Evolution* **47**, 1557–1571 (1993).
6. Guiasu, R. C. & Winterbottom, R. Osteological Evidence for the Phylogeny of Recent Genera of Surgeonfishes. *Copeia* **1993**, 300–312 (1993).
7. Winterbottom, R. Myological Evidence for the Phylogeny of Recent Genera of Surgeonfishes (Percomorpha, Acanthuridae), with Comments on the Acanthuroidei. *Copeia* **1993**, 21–39 (1993).
8. Tyler, J. C. & Sorbini, L. On the relationships of *Eonaso*, an Antillean fossil surgeon fish (Acanthuridae). *Studi e Ricerche sui Giacimenti Terziari di Bolca, Museo Civico di Storia Naturale di Verona* **7**, 35–42 (1998).
9. Tyler, J. C., Johnson, G. D., Nakamura, I. & Collette, B. B. Morphology of *Luvarus imperialis* (Luvaridae) with a phylogenetic analysis of the Acanthuroidei (Pisces). *Smithsonian Contributions to Zoology* **485** (1989).
10. Smith, J. L. B. Fishes of the sub-family Nasinae with a synopsis of the Prionurinae. *Ichthyological Bulletin (Rhodes University, Grahamstown)* **32**, 634–682 (1966).
11. Mok, H.-K. Gut Patterns of the Acanthuridae and Zanclidae. *Japan Journal of Ichthyology* **23**, 215–219 (1977).
12. Tyler, J. C. The Miocene fish *Marosichthys*, a putative tetraodontiform, actually a perciform surgeon fish (Acanthuridae) related to the recent *Naso*. *Beaufortia* **47**, 1–10 (1997).
13. Tyler, J. C. & Bannikov, A. F. A new species of the surgeon fish genus *Tauichthys* from the Eocene of Monte Bolca, Italy (Perciformes, Acanthuridae). *Bollettino del Museo Civico di Storia Naturale di Verona* **24**, 29–36 (2000).
14. Agassiz, L. Kritische revision der Ittiolitologia Ve- ronese abgebildenten fossilen Fische. *Neues Jahrbuch für Mineralogie, Geognosie, Geologie und Petrefaktenkunde,* **1835**, 290–316 (1835).
15. Carnevale, G. & Tyler, J. C. A new moorish idol (teleostei, Zanclidae) from the Eocene of Bolca, Italy. *Rivista Italiana di Paleontologia e Stratigrafia* **130**, 19–33 (2024).
16. Carnevale, G., Bannikov, A. F., Marramà, G., Tyler, J. C. & Zorzin, R. The Pesciara-Monte Postale Fossil-Lagerstätte: 2. Fishes and other vertebrates. *Rendiconti della Società Paleontologica Italiana* **4**, 37–63 (2014).
17. Blot, J. & Tyler, J. C. New genera and species of fossil surgeon fishes and their relatives (Acanthuroidei, Teleostei) from the Eocene of Monte Bolca, Italy, with application of the

Blot formula to both fossil and recent forms. *Studi e Ricerche sui Giacimenti Terziari di Bolca* **6**, 13–92 (1990).
18. Tyler, J. C. Redescription and basal phylogenetic placement of the acanthurid surgeon fish *Gazolaichthys vestenanovae* from the Eocene of Monte Bolca, Italy (Perciformes; Acanthuroidea). *Studi e Ricerche sui Giacimenti Terziari di Bolca* **11**, 97–117 (2005).
19. Hussakof, L. *Zebrasoma deani*, a fossil surgeon-fish from the West Indies. *Bulletin American Museum Natural History* **23**, 125–126 (1907).
20. Tyler, J. C. A new genus and species of surgeon fish (Acanthuridae) with four dorsal-fin spines from the Eocene of Monte Bolca, Italy. *Studi e Ricerche sui Giacimenti Terziari de Bolca* **8**, 257–268 (1999).
21. Sorbini, L. & Tyler, J. C. A new genus and species of Eocene surgeon fish (Acanthuridae) from Monte Bolca, Italy, with similarities to the Recent *Zebrasoma*. *Studi e Ricerche sui Giacimenti Terziari di Bolca* **7**, 7–19 (1998).
22. Carnevale, G. & Tyler, J. C. The caudal skeleton of *Arambourgthurus scombrurus* (Arambourg, 1967), a Paleogene oceanic surgeonfish. *Proceedings of the Biological Society of Washington* **131**, 101–110 (2018).
23. de Beaufort, L. F. On a collection of marine fishes from the Miocene of South Celebes. *Geologische Onderzoekingen in den Oostelijken Oost-Indischen Archiepel* 117–148 (1926).
24. Danil'chenko, P. G. Bony fishes of the Maikop deposits of the Caucasus. *Trudy Paleontologicheskogo Instituta* **78**, 1–247 (1960).
25. Agassiz, L. Recherches Sur Les Poissons Fossiles. *Tome IV (10me, 12me livraison)* 1–16 (1838).
26. Bannikov, A. F. & Tyler, J. C. A New Species of the Luvarid Fish Genus *Avitoluvarus* (Acanthuroidei, Perciformes) from the Eocene of the Caucasus in Southwest Russia. *Proceedings of the Biological Society of Washington* **114**, 579–588 (2001).
27. Alfaro, M. E. *et al.* Explosive diversification of marine fishes at the Cretaceous-Palaeogene boundary. *Nature Ecology and Evolution* **2**, 688–696 (2018).
28. Betancur-R., R. *et al.* The Tree of Life and a New Classification of Bony Fishes. *PLoS Currents* **5**, 0–45 (2013).
29. Betancur-R., R. *et al.* Phylogenetic classification of bony fishes. *BMC Evolutionary Biology* **17**, 1–40 (2017).
30. Ghezelayagh, A. *et al.* Prolonged morphological expansion of spiny-rayed fishes following the end-Cretaceous. *Nature Ecology and Evolution* **6**, 1211–1220 (2022).
31. Hughes, L. C. *et al.* Comprehensive phylogeny of ray-finned fishes (Actinopterygii) based on transcriptomic and genomic data. *Proceedings of the National Academy of Sciences* **115**, 6249–6254 (2018).
32. Near, T. J. *et al.* Resolution of ray-finned fish phylogeny and timing of diversification. *Proceedings of the National Academy of Sciences* **109**, 13698–13703 (2012).
33. Near, T. J. *et al.* Phylogeny and tempo of diversification in the superradiation of spiny-rayed fishes. *Proceedings of the National Academy of Sciences* **110**, 12738–12743 (2013).
34. Rabosky, D. L. *et al.* An inverse latitudinal gradient in speciation rate for marine fishes. *Nature* **559**, 392–395 (2018).
35. Siqueira, A. C., Bellwood, D. R. & Cowman, P. F. Historical biogeography of herbivorous coral reef fishes: The formation of an Atlantic fauna. *Journal of Biogeography* **46**, 1611–1624 (2019).

36. Sorenson, L., Santini, F., Carnevale, G. & Alfaro, M. E. A multi-locus timetree of surgeonfishes (Acanthuridae, Percomorpha), with revised family taxonomy. *Molecular Phylogenetics and Evolution* **68**, 150–160 (2013).
37. Bellwood, D. R., Hoey, A. S., Bellwood, O. & Goatley, C. H. R. Evolution of long-toothed fishes and the changing nature of fish-benthos interactions on coral reefs. *Nature Communications* **5**, 1–6 (2014).
