# Supplementary material for: Ecological and genomic signatures of the convergent evolution of planktivory in fossil and living reef fishes over deep time
Source: Nat Commun. 2026 May 22;17:6739. doi: 10.1038/s41467-026-73110-3 (PMC13385881; doi:10.1038/s41467-026-73110-3)
Supplement: Supplementary file 2 — Descriptions of Additional Supplementary Files [file 41467_2026_73110_MOESM2_ESM.pdf]

## Descriptions for Supplementary Data 1-5

**Supplementary Data 1: List of specimens sequenced for exon capture.** This table includes the family, genus, and species of each studied individual, alongside their respective museum catalog number, voucher number, the institution affiliated with the sample (with corresponding institution code), collection locality, as well as the associated BioProject, BioSample, and SRA accession numbers.

**Supplementary Data 2: List of specimens sequenced for short-read genomes.** This table includes the family, genus, and species of each studied individual, alongside their respective museum catalog number, voucher number, the institution affiliated with the sample (with corresponding institution code), collection locality, as well as the associated BioProject, BioSample, and SRA accession numbers.

**Supplementary Data 3: List of 7,207 genes from the BUSCO Actinopterygii OrthoDatabase version 12, their status in our reference genome, and their corresponding BUSCO odb10 identifiers.** The table reports the 'BUSCO ID odb12' representing the gene identifier in the Actinopterygii odb12 database, the 'Status' indicating whether each gene is complete, duplicated, fragmented, or missing, the 'Reference Genome Sequence' showing the matching gene identified in the A. chirurgus reference genome, and the 'BUSCO ID odb10' providing the corresponding gene identifier in the older odb10 database.

**Supplementary Data 4: List of the 302 positive selected genes obtained in aBSREL with its corresponding biological process, molecular functions and cellular components.** Functional classification of candidate genes based on PANTHER analysis. The table shows the PANTHER 'Family ID' assigned to each gene, the number of 'Mapped ID's (i.e., the gene alignment used), the 'Source' of the mapped gene (i.e., genome, BUSCO odb10, or odb12), and the 'Family Name' describing the protein family. The 'Genes' column lists the genes included in each family. Gene functions were annotated using 'PANTHER GO-Slim' terms, which provide simplified summaries of Gene Ontology categories for 'Molecular Function', 'Biological Process', and 'Cellular Component'. Additionally, each gene was assigned a 'PANTHER Protein Class' indicating the general role of the encoded protein.

**Supplementary Data 5: List of the 39 identified as convergently evolving based on aBSREL analysis.** For each gene, the table shows the PANTHER 'Family ID', 'Mapped ID' gene identifier(s), 'Gene name', the 'Source' database and 'Match ID' to the associated reference genome and BUSCO odb10 database. Columns also include information on rate shifts inferred from Relative Evolutionary Rates (RER), with notes on whether acceleration or deceleration was detected and in which lineages. The number of sites under episodic diversifying selection detected by MEME is also reported. Functional annotations include the protein 'family name', 'number of genes in the family', and GO-Slim terms for 'Molecular Function', 'Biological Process', and 'Cellular Component'. Additional broad biological categories are included to contextualize gene functions as well as expression patterns and phenotypic information for some genes.
