# Supplementary material for: Ecological and genomic signatures of the convergent evolution of planktivory in fossil and living reef fishes over deep time
Source: Nat Commun. 2026 May 22;17:6739. doi: 10.1038/s41467-026-73110-3 (PMC13385881; doi:10.1038/s41467-026-73110-3)
Supplement: Supplementary file 8 — Reporting Summary [file 41467_2026_73110_MOESM8_ESM.pdf]

nature portfolio

Corresponding author(s): Ricardo Betancur-R.

Last updated by author(s): Apr 6, 2026

Reporting Summary

Nature Portfolio wishes to improve the reproducibility of the work that we publish. This form provides structure for consistency and transparency in reporting. For further information on Nature Portfolio policies, see our Editorial Policies and the Editorial Policy Checklist.

Statistics

For all statistical analyses, confirm that the following items are present in the figure legend, table legend, main text, or Methods section.

n/a	Confirmed	
☐	☒	The exact sample size (*n*) for each experimental group/condition, given as a discrete number and unit of measurement
☐	☒	A statement on whether measurements were taken from distinct samples or whether the same sample was measured repeatedly
☐	☒	The statistical test(s) used AND whether they are one- or two-sided<br>*Only common tests should be described solely by name; describe more complex techniques in the Methods section.*
☒	☐	A description of all covariates tested
☐	☒	A description of any assumptions or corrections, such as tests of normality and adjustment for multiple comparisons
☐	☒	A full description of the statistical parameters including central tendency (e.g. means) or other basic estimates (e.g. regression coefficient) AND variation (e.g. standard deviation) or associated estimates of uncertainty (e.g. confidence intervals)
☐	☒	For null hypothesis testing, the test statistic (e.g. *F*, *t*, *r*) with confidence intervals, effect sizes, degrees of freedom and *P* value noted<br>*Give P values as exact values whenever suitable.*
☐	☒	For Bayesian analysis, information on the choice of priors and Markov chain Monte Carlo settings
☒	☐	For hierarchical and complex designs, identification of the appropriate level for tests and full reporting of outcomes
☒	☐	Estimates of effect sizes (e.g. Cohen's *d*, Pearson's *r*), indicating how they were calculated

Our web collection on statistics for biologists contains articles on many of the points above.

Software and code

Policy information about availability of computer code

Data collection	Illumina HiSeq 4000 for exon capture, Illumina NovaSeq 6000 for short-reads, PacBio Sequell II (long reads) and Illumina HiSeq X (HiC) for chromosome-level genome, NovaSeq 6000 for transcriptome.
Data analysis	The code used is available at https://github.com/lilychughes/FishLifeExonCapture/ and Figshare https://doi.org/10.6084/m9.figshare.29594255.<br>The code includes all open source software packages utilized as mentioned in the method section and listed here:<br>Trimmomatic v0.39<br>BWA v.0.7.17<br>SAMtools v.1.9<br>Velvet v.1.2.10<br>aTRAM v.2.2<br>Trinity v.2.2<br>CD-HIT-EST v.4.8.1<br>Exonerate v.2.4.0<br>MACSE v.2.03<br>Geneious Prime v.2024.0<br>AMAS<br>FastTree-2<br>PartitionFinder2<br>RAxML v.8.2.11<br>ASTRAL-III

TNT v.1.5
MrBayes v.3.2.7a
TRACER v.1.7
Figtree v.1.4.4
Hifiasm v.0.15.4-r347
blobtools2 v.1.1.1
purge_dups3 v.1.2.5
Omni-C HiRise
BUSCO v.6.0.0
RepeatModeler v.2.0.1
RepeatMasker v.4.1.0
AUGUSTUS v.2.5.5
SNAP v.2006-07-28
STAR v.2.7
MAKER2
tRNAscan-SE v.2.05
MaSuRCA v.4.0.8
RagTag v.2.1.0
SeqKit v2.8.2
HMMER v.3.1
OrthoFinder
HyPhy
RStudio v.4.4.2

For manuscripts utilizing custom algorithms or software that are central to the research but not yet described in published literature, software must be made available to editors and reviewers. We strongly encourage code deposition in a community repository (e.g. GitHub). See the Nature Portfolio guidelines for submitting code & software for further information.

Data

Policy information about availability of data

All manuscripts must include a data availability statement. This statement should provide the following information, where applicable:

- Accession codes, unique identifiers, or web links for publicly available datasets
- A description of any restrictions on data availability
- For clinical datasets or third party data, please ensure that the statement adheres to our policy

The genomic data generated in this study have been deposited in the NCBI database under the umbrella BioProject PRJNA1433022. Raw sequence reads for exon-capture and short-reads are under the BioProject PRJNA1418135. Accession numbers for exon-capture data and short-read genomes are provided in Supplementary Data 1 and 2, respectively. Museum collection numbers and the collected geographic locations for all sampled specimens can also be found in Supplementary Data 1 and 2. The voucher specimen of Acanthurus chirurgus used to generate the chromosome-level genome in this study is deposited at Scripps Institution of Oceanography (SIO), under collection number SIO 24-10. PacBio and Hi-C raw reads for the A. chirurgus genome are also available under BioProject PRJNA1418135. The phased haplotypes assemblies are deposited under BioProjects PRJNA1430673 (principal haplotype, accession number JBVQTB000000000) and PRJNA1430672 (alternate haplotype, accession number JBVQTC000000000). All data generated and analyzed during this study are available on Figshare (https://doi.org/10.6084/m9.figshare.29594255), including exon and single-copy ortholog alignments, phylogenetic trees, input files for comparative analyses, and chromosome-level genome annotation files. Source data for the main figures are provided as a Source Data file and on Figshare.

Research involving human participants, their data, or biological material

Policy information about studies with human participants or human data. See also policy information about sex, gender (identity/presentation), and sexual orientation and race, ethnicity and racism.

Reporting on sex and gender	NA
Reporting on race, ethnicity, or other socially relevant groupings	NA
Population characteristics	NA
Recruitment	NA
Ethics oversight	NA

Note that full information on the approval of the study protocol must also be provided in the manuscript.

Field-specific reporting

Please select the one below that is the best fit for your research. If you are not sure, read the appropriate sections before making your selection.

☐ Life sciences ☐ Behavioural & social sciences ☒ Ecological, evolutionary & environmental sciences

For a reference copy of the document with all sections, see nature.com/documents/nr-reporting-summary-flat.pdf

Ecological, evolutionary & environmental sciences study design

All studies must disclose on these points even when the disclosure is negative.

Study description	This study used ethanol-preserved tissues from museum collections for DNA extraction, exon sequencing, and short-read genome sequencing, as well as one flash-frozen tissue sample to generate a chromosome-level genome.
Research sample	We sequenced a total of 57 acanthuriforms and 9 outgroups. Sequences for 25 species were downloaded from GenBank. Finally 32 fossil species from museums were examined.
Sampling strategy	We focused our study on representing the diversity across the order Acanthuriformes. To this end, we attempted to include all valid species in the group by requesting tissue samples from museums worldwide.
Data collection	This study relied on ethanol-preserved tissues from museum collections for DNA extraction, library preparation, and sequencing. In addition, we obtained flash-frozen muscle tissue from an individual captured with a hand net to generate a chromosome-level genome assembly.
Timing and spatial scale	We used samples from all around the world sampled from 2014 to 2023.
Data exclusions	No data was excluded from the analysis.
Reproducibility	Genomic sequencing of individual samples was not replicated.
Randomization	Randomization is not applicable to genomic sequencing of individuals.
Blinding	Blinding is not applicable to genomic sequencing of individiuals.

Did the study involve field work? ☒ Yes ☐ No

Field work, collection and transport

Field conditions	We obtained flash-frozen muscle tissues from an individual of the Doctorfish tang (Acanthurus chirurgus), captured using a hand net.
Location	Florida Keys (24°59.564 N, 80°25.753 W), United States.
Access & import/export	Phillipp Rauch collected the sample near the shore using a hand net. The wild fish Acanthurus chirurgus was collected under a valid Commercial Saltwater Products License (SPLicense #SP-188970) with Marine Life endorsement (MLD-299), issued by the Florida Fish and Wildlife Conservation Commission. This license authorizes the legal collection of marine ornamental species in Florida waters. The collection complied with relevant local and state regulations.
Disturbance	NA

Reporting for specific materials, systems and methods

We require information from authors about some types of materials, experimental systems and methods used in many studies. Here, indicate whether each material, system or method listed is relevant to your study. If you are not sure if a list item applies to your research, read the appropriate section before selecting a response.

Materials & experimental systems

n/a	Involved in the study	
☒	☐	Antibodies
☒	☐	Eukaryotic cell lines
☐	☒	Palaeontology and archaeology
☐	☒	Animals and other organisms
☒	☐	Clinical data
☒	☐	Dual use research of concern
☒	☐	Plants

Methods

n/a	Involved in the study	
☒	☐	ChIP-seq
☒	☐	Flow cytometry
☒	☐	MRI-based neuroimaging

Palaeontology and Archaeology

Specimen provenance	All specimens used come from museum collections.
Specimen deposition	All specimens used come from museum collections.
Dating methods	No fossils were dated.

☐ Tick this box to confirm that the raw and calibrated dates are available in the paper or in Supplementary Information.

Ethics oversight	NA

Note that full information on the approval of the study protocol must also be provided in the manuscript.

Animals and other research organisms

Policy information about studies involving animals; ARRIVE guidelines recommended for reporting animal research, and Sex and Gender in Research

Laboratory animals	No laboratory animals were used.
Wild animals	Doctorfish tang
Reporting on sex	Not collected.
Field-collected samples	We obtained flash-frozen muscle tissues from an Doctorfish tang on January of 2023. The voucher specimen was deposited at Scripps Institution of Oceanography (SIO), collection number SIO 24-10. Subsequently, we outsourced the DNA and RNA extractions, library preparations, sequencing, assembly, and annotation to Cantata Bio LLC and generate a chromosome-level genome and transcriptome for the species.
Ethics oversight	The University of Oklahoma Institutional Animal Care and Use Committee reviewed and approved protocol #2022-0239

Note that full information on the approval of the study protocol must also be provided in the manuscript.

Plants

Seed stocks	NA
Novel plant genotypes	NA
Authentication	NA
