# Supplementary material for: Ecological and genomic signatures of the convergent evolution of planktivory in fossil and living reef fishes over deep time
Source: Nat Commun. 2026 May 22;17:6739. doi: 10.1038/s41467-026-73110-3 (PMC13385881; doi:10.1038/s41467-026-73110-3)
Supplement: Supplementary file 9 — Transparent Peer Review file [file 41467_2026_73110_MOESM9_ESM.pdf]

nature portfolio

Peer Review File

Ecological and genomic signatures of the convergent evolution of planktivory in fossil and living reef fishes over deep time

Corresponding Author: Professor Ricardo Betancur-R

This file contains all reviewer reports in order by version, followed by all author rebuttals in order by version.

Attachments originally included by the reviewers as part of their assessment can be found at the end of this file.

Version 0:

Reviewer comments:

Reviewer #1

(Remarks to the Author)
Please see attachment

Reviewer #2

(Remarks to the Author)
I co-reviewed this manuscript with one of the reviewers who provided the listed reports. This is part of the Nature Communications initiative to facilitate training in peer review and to provide appropriate recognition for Early Career Researchers who co-review manuscripts.

Reviewer #3

(Remarks to the Author)
General comments
The manuscript by Santaquiteria et al. examines the evolutionary basis for the convergence towards planktivory in coral reef fishes, with a focus on Acanthuriformes. By collating an impressive amount of genomic and morphological data, the authors set out to estimate the timing, frequency, and biogeography of dietary shifts; test evolutionary rates associated with planktivory (i.e. speciation, extinction, and transitions); assess correlations between trophic transitions and paleoclimatic trends; and identify the potential genomic basis of selection for planktivory. Overall, I found this to be a very interesting, well written, and well executed manuscript. The dataset assembled is both comprehensive and valuable, and the analyses offer important insights into the evolutionary history of a key reef fish group. I commend the authors on the herculean effort of compiling and analysing such a large dataset, and I believe the manuscript will be of broad interest to ichthyologists and evolutionary biologists alike.
That said, I will limit my evaluation to the phylogenetic and macroevolutionary components of the study, as I do not have the necessary expertise to comment on the genomic analyses (e.g. signatures of selection and gene annotations). Below I outline both positive aspects and points that I think should be strengthened before publication.

Major points
One of the major strengths of this manuscript is the integration of morphological and molecular evidence to produce divergence time estimates. This approach provides more realistic age estimates for Acanthuriformes compared to earlier studies (including my own), where inferred crown ages were often older than supported by the fossil record. It is encouraging to see that combined datasets reconcile molecular and paleontological perspectives, in line with recent findings for other reef-associated lineages (e.g. Cantalice et al. 2022 – BioScience). This represents a significant advance in our understanding of reef fish evolution and helps resolve mismatches identified in earlier analyses. The manuscript therefore highlights the value of integrating fossil and extant species data, and I strongly support this approach.
Despite agreeing with most of the results and interpretations, I do have a few suggestions for improvement. The first relates to the interpretation of the paleoclimatic model results. Based on Figure 1B and Table S8, the lambda model appears to be

consistently better supported than climate-dependent models, both for the MCC tree and across the posterior sample of trees. If this is correct, the evidence does not support a strong role for temperature in driving evolutionary differences between feeding modes. Statements such as "…planktivory diversified during periods of global cooling" (lines 34-35) seem to be based largely on lineage-through-time plots, which in my view provide weak evidence compared to the formal model comparison. Unless I am misinterpreting the results (which I might), I recommend toning down or removing claims of a causal relationship between paleoclimatic shifts and trophic evolution, particularly in the abstract and conclusions. The paper already makes a substantial contribution without leaning on this inference, which currently feels unsupported by the analyses.

Related to this, it would also be useful to better integrate the results of the transition models. Based on the simmap analysis, the equal-rates model of transition between dietary states is preferred. However, when discussing whether planktivory is an evolutionary dead end, the interpretation relies mainly on the HiSSE model results, which show higher transition rates from non-planktivores to planktivores. As the results acknowledge, this pattern is largely driven by transitions and reversals in the genus Naso. The two approaches seem to suggest different dynamics, yet the manuscript does not fully reconcile these outcomes. While I agree with the overall conclusion that planktivory does not appear to be an evolutionary dead end in Acanthuriformes, I suggest more explicitly discussing why simmap and HiSSE may yield different inferences, and what this means for the robustness of the conclusions.

Finally, I believe some parts of the Results and Discussion section could be streamlined, as the manuscript is already quite long. For example, the opening paragraphs of the sections "No evolutionary dead end associated with the evolution of planktivory" and "Signatures of convergence in positive selection following transitions to planktivory" feel somewhat repetitive and read more like background introduction. Reducing or condensing these sections would improve readability and help the main findings stand out more clearly.

Minor Points

Line 81: The parentheses is not closed. This also happens in other instances (e.g. lines 291, 578, 630), so I am assuming this stems from the automatic reference maker. Please double check throughout the text.

Line 101: I suggest changing from "ecological histories" to just "ecologies" for simplicity.

Lines 106-107: I appreciate that my previous work relied on taxonomic placement of fossil species rather than the morphological dataset used here, but it was based on the available literature, not on "subjective interpretation of photographic records" as stated. I suggest rephrasing this to more accurately reflect the approach taken. In addition, the order of citations in this sentence appears to be incorrect (I believe ref. 50 rather than 51 is intended?), please double check.

Line 108: Suggested change from "originated" to "diversified" if the authors agree.

Lines 123-127: Perhaps numbering the specific goals might help with clarity; e.g. "Specifically, we 1) estimated…; 2) tested…; 3) assessed…; 4) identified…"

Line 132: Suggestion - "…acanthuriform trophic diversity has been shaped…"

Lines 146-148: I suggest breaking this sentence in two; e.g. "…and 9 outgroups. Of these…"

Line 152 (and throughout the results): Considering the amount of supplementary material, it might be a good idea to order the supp figures in order of appearance in the main text. The current order made it hard to scroll back and forth while reading the main text.

Lines 157-158: I thought this was super interesting that the morphological dataset groups Luvarus with the (mostly planktivorous) Naso. Could this be an indication of how morphological convergence towards planktivory might mislead the analysis and, therefore, shows how important it is to also include the genetic data? Just a thought.

Lines 169-170: This is a massive amount of time (well done on the effort!) and yet, some subsets did not achieve the desired ESS. From my experience, adding the fossils makes it hard for the analysis to converge. Just to be clear, I am in no way suggesting that you should rerun the analyses; but just wondering out of curiosity if you compared an analysis with and without the fossils to see if convergence would be achieved faster?

Line 200: "diets" instead of "lifestyle"?

Lines 269-272: Yes, I agree that this result is mostly being driven by Naso. Therefore, it might be relevant to note that some Naso species (specifically Naso brevirostris) browse on benthic macroalgae as juveniles and only then transition to being more planktivore (see Clements 1991 for example). Perhaps this demonstrates how diet in this genus may be more labile than usual?

Lines 273-276: This sentence is a little awkward, can you please clarify?

Lines 296-297: Where does the information that fishes on the Paleocene fed on "filamentous or macrophytic algae as well as detritus and microbes" come from? Just curious.

Line 305: How do you know that benthic-feeding lineages went extinct at this time? The fossil-tip analysis only suggests these lineages were present at that time, but not necessarily when they went extinct. Please correct me if I am wrong.

Line 311: Suggestion "However, it is important to recognize that strong taphonomic biases…"

Line 317: In this case, are you talking about speciation rates or just number of lineages through time? Please clarify.

Line 360: "estimated" instead of "estimationed".

Materials and Methods: Some of the sections in the Supplementary Materials seem almost the same (with a little bit more detail) as in the main text. Is there a need for these two versions? My suggestion would be to consolidate the important information in the main text, and only have auxiliary info (e.g. the list of morphological characters, tables and figures) as supplementary.

Lines 614-621: Sorry if I am missing something here, but these numbers are not matching. In the previous methods section, you talk about 56 in-group species sequenced, but here 91 newly sequenced species are mentioned. Then at the end you talk about 80 in-group species and 9 outgroup species and in parenthesis there are 90 species in total. Please double check these numbers as they are confusing.

Line 647: Should it be "see Supplementary material for the list…"?

Lines 688-690: Suggested rephrasing: "Because these analyses ran for over a year and Subset 2 based on the Scheme 2 had not reached satisfactory ESS values, we removed it from all downstream analyses."

Line 691: I am assuming the trees were sampled from the posterior distribution post-burnin, right?
Lines 690-693: Out of curiosity, if you combine the posterior distributions from these subsets, do you get ESS above 200? This is essentially what your sample of the posterior represents (the 10,000 trees per Scheme).
Lines 700 and 717: Perhaps cite the literature sources?
Lines 758-762: "…rates independent of…" and "…dependent on…"
Figure S8: A part of legend is repeated, please double check.

I hope this review was helpful.
Alexandre Siqueira

Version 1:

Reviewer comments:

Reviewer #1

(Remarks to the Author)
We appreciate the author's thoroughness in addressing all our comments and in taking the time to rerun the BUSCO analyses on the updated dataset. We believe this study to be very interesting and important to better understand the evolutionary history and diversification of reef fishes. This final version is much clearer and more concise, and we are happy with all the revisions done. We suggest this manuscript for acceptance.

Reviewer #2

(Remarks to the Author)
I co-reviewed this manuscript with one of the reviewers who provided the listed reports. This is part of the Nature Communications initiative to facilitate training in peer review and to provide appropriate recognition for Early Career Researchers who co-review manuscripts.

Reviewer #3

(Remarks to the Author)
I thank the authors for considering and addressing my comments from the first round of reviews. I have checked the responses to all reviewers and consider them satisfactory. The manuscript is substantially improved and will be a valuable addition to the literature. I only have a few very minor points to suggest:
Line 203 of the ms version after accepting all changes: remove "the" from the sentence "With the newly inferred time trees including fossil and living acanthuriform species, we reconstructed their diets to investigate the number of times the planktivory evolved, as well as the regions and timing of these transitions".
Line 276: "highlighting an exceptional dietary lability in Naso".
Line 300-303: Detritus feeding has been suggested as a more recent acquisition (see Bellwood et al. 2014 Nat Comms), so unless there is other fossil evidence to indicate this type of feeding in the Eocene, I suggest changing the sentence to: "These analyses revealed that acanthuriforms gradually started diversifying during the Paleocene and early Eocene (66–50 Ma), particularly benthic lineages whose trophic ecology (based on inference from extant relatives 11) might have involved filamentous or macrophytic algae."
Line 327: I suggest adding "potential" to the sentence "Although we observe a potential temporal correlation between the evolution of planktivory and major climatic transitions, our model fitting analyses indicate split support between climate-dependent and climate-independent models".
Line 374: "Understanding whether trophic transition convergence is mirrored at the molecular level…", change transition to singular?
Lines 405-406: suggested change "Among these, 39 convergently underwent positive selection in at least two lineages (Fig. 3 and Table S11)".

I look forward to seeing this published!

Open Access This Peer Review File is licensed under a Creative Commons Attribution 4.0 International License, which permits use, sharing, adaptation, distribution and reproduction in any medium or format, as long as you give appropriate credit to the original author(s) and the source, provide a link to the Creative Commons license, and indicate if changes were made.
In cases where reviewers are anonymous, credit should be given to 'Anonymous Referee' and the source.
The images or other third party material in this Peer Review File are included in the article's Creative Commons license, unless indicated otherwise in a credit line to the material. If material is not included in the article's Creative Commons license and your intended use is not permitted by statutory regulation or exceeds the permitted use, you will need to obtain permission directly from the copyright holder.
To view a copy of this license, visit https://creativecommons.org/licenses/by/4.0/

REVIEWER COMMENTS

Reviewer #1(Remarks to the Author):

Santaquiteria et al. Ecological and genomic signatures of the convergent evolution of planktivory in fossil and living fishes over deep time.
In this study, Santaquiteria et al present a comprehensive study that aims to investigate the evolution of planktivory in Acanthuriformes (specifically focused on surgeonfishes, unicornfishes, tangs, luvars, and moorish idols) while also testing the hypothesis that pelagic planktivory represents an evolutionary dead end. This study integrates genomic and morphological data across more than 80 species encompassing approximately 93% of the group's diversity, representing one of the most complete phylogenomic studies of Acanthuriformes to date. Among their most notable results, they find evidence that rejects the hypothesis of pelagic planktivory as an evolutionary dead end, and find shared molecular pathways associated with shifts in trophic positions that evolved by parallel or convergent evolution. Overall, the manuscript is well written and will serve as an important study to enhance our understanding of the ecological and genetic drivers shaping dietary evolution in marine fishes. With the declining costs of sequencing, more studies will benefit from this framework to combine morphological and genomic data to better understand the drivers of diversification and obtain higher resolution phylogenies. The methods and data provided are thorough and clear. However, the methods provided in the manuscript and supplementary information greatly overlap and could possibly be updated in the main text to reduce the supplementary information if the word count allows.

Response: Thank you for the positive comments. We have addressed all concerns raised below and removed the redundant information from the Supplementary Methods.

Specific comments:

● Scheme 1 versus Scheme 2 should be introduced with more clarity – it took a few reads to figure out the difference and the best explanation came from the methods (section: *Total-evidence dating analyses and phylogenetic uncertainty*)

Response: Thanks for this. We're also now stating more clearly in the Results section ("*Phylogenomic total-evidence dating resolves acanthuriform relationships and reveals post-K–Pg diversification.*"), as follows: "Given anatomical evidence supporting the latter arrangement [1], we present comparative results based on this topology (the two Eocene fossils sister to Zanclidae + Acanthuridae: 'Scheme 1' hereafter), with alternative results (the two Eocene fossils sister to Acanthuridae: 'Scheme 2' hereafter) reported in the Supplementary Materials (Figs. S10, S14, S16, S17, S19–S21; Tables S5–S8)."

● We suggest going into more depth on the reasoning behind pelagic planktivory as an "evolutionary dead end" (~Line 67-73) in the introduction. This is better described later in the paper (Line 236-243) and would be useful to move this earlier or expand in the introduction.

Response: Thanks, this is a great suggestion. We have revised the Introduction to more clearly articulate the conceptual basis for why pelagic planktivory has been proposed as a potential evolutionary dead end. We now explain that "Adopting a water column lifestyle may offer short-term advantages, but in the long term, it can create an evolutionary trap due to reduced speciation, elevated extinction, or both, particularly in structurally homogeneous pelagic habitats with fewer opportunities for ecological partitioning," and include brief examples from the literature demonstrating where this pattern is and is not supported across reef fish clades. We also removed the overlapping explanation from the Results section to avoid redundancy.

● Line 141-144: The math doesn't seem to add up in the number of species. You mention there are "80 ingroup species" and "9 outgroup" which would be a total of 89 species, however, you follow up with "of these, 65 species have data for 998 'FishLife' exon markers 54, which also incorporate 22 legacy markers, whereas 25 species include data only for legacy markers". 65 + 25 would be a total of 90 species, not 89? Similarly, in Line 599 you mention "91 newly sequenced species". Check the numbers as they are not consistent.

Response: Thanks for pointing this out. We have 24 species with legacy markers and 65 species with both exon and legacy markers, totaling 89 species. We have corrected this as follows: "The molecular-only matrix contains 80 ingroup species (representing approximately 93% of the group's diversity) and 9 outgroups. Of these, 65 species have data for 998 'FishLife' exon markers [2], which also incorporate 22 legacy markers, whereas 24 species include data only for legacy markers."

Regarding the "91 newly sequenced species," this was referring to the expanded dataset, which does not consist solely of newly sequenced taxa. We have revised the sentence and change it from 91 to 66 taxa (65 species, as *Acanthurus tractus* is duplicated): "To increase the number of species, we generated an expanded matrix combining the 66 newly sequenced taxa (reduced matrix) with sequences for up to 9 markers for 59 species obtained from GenBank."

● We suggest to rerun BUSCO with the updated actinopterygii dataset (used odb9 but odb12 is available). This should not be too time consuming but would make it stronger and more up to date.

Response: Thank you for this suggestion. We reran BUSCO with the updated Actinopterygii dataset (odb12) for both the chromosome-level genome and the short-read genomes, which required more than a month of additional analyses. The results are now summarized in Lines 568–597 and 1263–1285. Briefly, completeness for our reference non-annotated genome changed from 98.8% to 98.1%, and for the annotated version from 92.2% to 85.7%. Short-read genomes now range from 67% to 97.6% completeness (vs. 74–98% before). Because odb12 includes more than 7,000 genes, we subsequently ran OrthoFinder to evaluate whether additional single-copy loci could be incorporated into the HyPhy positive selection analyses. This resulted in 1,666 single-copy genes, of which 1,532 were already included in our genome dataset. The remaining 134 were newly analyzed for this version, 24 of which were found to be under positive selection, with 1 showing convergent signatures across at least two planktivorous lineages.

● Line 395: You mention *Naso* exhibited the highest number of shared PSG's with other planktivors, however in Figure 3 it seems to be only one *Naso* species (*Naso mcdadei*). We suggest discussing this further, or clarifying whether the UpSet plot of Figure 3 refers to Scenario 1 specifically.

Response: The point in the UpSet plot of Fig. 3 represents the entire *Naso* clade, not just *Naso mcdadei*. We originally positioned the point near the center of the clade to reflect this, but we agree that it could be misinterpreted. Therefore, we have modified Figure 3 (now Fig. 3A) to make this clearer. Additionally, Figure 3 combines results from both Scenario 1 and Scenario 2, and we have updated the figure caption to clarify this.

● Figure 1: (Panel A)
  - ○ Black and Red lines are described in Panel B but should be moved to Panel A.
  - ○ Phylogeny tips are a bit hard to see with the green and blue line going through them. We suggest making the tips slightly larger to adequately see the tip colors.

Response: We believe it is more appropriate to retain the curve descriptions in Panel B, as that is where the model is fitted to the two temperature curves. However, we have added a clarification to the caption: "curves for tropical temperature (red) and global average temperature (black), illustrated in Panel A." We moved the tip circles to the front in Panel A to improve visibility, so they no longer overlap with the green and blue lines.

● Figure 2:
  - ○ This figure is not very clear. Figure caption could use more detail. Colors in the key below the figure don't seem to fully match the phylogeny or those shown on the black bars. Blue colors in general are very similar and hard to tell apart. The colors overlaid on the map are hard to see as well. It is unclear what the key at the bottom of the figure is intended to illustrate. Lastly, "CIP" is missing from the figure caption.

Response: The colors on the black bars represent present-day areas (WIO, CIP, CP, TEP, WA, EA, and WA), while the colors in the key at the bottom correspond to combined ancestral areas. We have clarified this both in the figure and in the figure caption. We also enhanced the visibility of the colors overlaid on the map and believe the blue shades are now sufficiently distinguishable. Finally, "CIP" has been added to the figure caption.

● Figure 3:
  - ○ Break this figure up into panel A, B, and C. Within the text you refer to blocks of this figure, but it is not aligned with how the figure is presented
  - ○ Line 853: change "Fig. 3A" to "Fig 3", OR alter Figure 3 as in comment above.
  - ○ Line 866: change "Fig. 3D" to "Fig 3", OR alter Figure 3. However, we're not sure what this is referring to in figure 3. Or did you mean Figure 4?

Response: We broke the figure in panel A and B and update the figure caption.
Line 853 we kept Fig. 3A.
Line 866 we changed Fig. 3D to Fig. 4B and S23.

● Figure 4:

○ Similar to the above comment, break it up in panels A and B to keep it consistent.

○ Although we appreciate the image of the fish, was there a specific reason this species was selected? We suggest mentioning its importance or presence in the caption.

Response: This figure has two panels now, A and B. We modified the caption accordingly and add an explanation of why we add an image of the Palette surgeonfish in the figure.

Minor Comments:

● Line 81: add ")" after "but see 25-27"

Response: Parenthesis added.

● Line 105-110: Long confusing sentence. Suggest splitting and rewording.

Response: We have revised these lines to improve clarity by splitting the sentence and rewording it. The revised text now reads: "Two studies—one including 18 fossil taxa subjectively placed from photographic records alongside 41 extant species [3], and another with 25 fossil taxa placed according to available literature alongside 72 extant species[4], rather than explicit morphological datasets—proposed that surgeonfishes originated in two distinct phases: first during the Paleocene–Eocene (~66–34 Ma), potentially due to ecological release after the Cretaceous–Paleogene (K–Pg) mass extinction, and again in the Oligocene–Miocene (~23–5 Ma), coinciding with the rise of extensive coral reef systems [5]."

● Lines 265-267: reword sentence: "While planktivory is considered an evolutionary destination, some unicornfishes, despite not further specializing their diet, can revert to their ancestral trophic states…"

Response: We have reworded the sentence as suggested.

● Lines 374-375: Is this sentence stating BUSCO scores run in both genome and protein modes? If so, we recommend rewording to say so instead of describing the protein BUSCO as a "decrease" from the initial assembly BUSCO, as these are two separate analyses.

Response: We now separated each analysis into two different sentences for clarity: "We assessed the genome completeness in BUSCO [97,98] based on the Actinopterygii database (odb12). Before annotation, BUSCO showed a completeness score of 98.1% (Fig. S23). When BUSCO was run on the annotated genome and predicted 23,548 protein-coding genes, the completeness was 85.7%."

● Line 615: add ")" after "supercomputers"

Response: Parenthesis added.

● Line 616: change “bootstrapping” to “bootstrap replicates”

Response: Changed.

● Line 638: change “bootstraps” to “bootstrap replicates”

Response: Changed.

● Lines 651-652: reword “combining molecules with morphology” to something like “a combination of morphology and molecular information”

Response: We have revised the sentence as suggested. It now reads: “After independently estimating phylogenies using morphology alone and using a dataset combining morphology and molecular information, we noticed that two fossils, †*Gazolaichthys vestenanovae* and †*Padovathurus gaudryi*, had incongruent placements in the resulting trees.”

● Line 670: take “of the 10” out of parentheses

Response: Parentheses removed.

● Lines 860-862: reword this sentence to something like “aBSREL is restricted to focal branches and therefore cannot detect whether a gene is also under selection in non-focal branches, so we complemented these analyses with BUSTED-Phenotype (BUSTED-PH).”

Response: We have now revised the sentence as follows: “aBSREL is restricted to focal branches and cannot detect positive selection in non-focal branches; we therefore complemented these analyses with BUSTED-Phenotype (BUSTED-PH).”

Reviewer #2 (Remarks to the Author):

I co-reviewed this manuscript with one of the reviewers who provided the listed reports. This is part of the Nature Communications initiative to facilitate training in peer review and to provide appropriate recognition for Early Career Researchers who co-review manuscripts.

Reviewer #3 (Remarks to the Author):

General comments

The manuscript by Santaquiteria et al. examines the evolutionary basis for the convergence towards planktivory in coral reef fishes, with a focus on Acanthuriformes. By collating an impressive amount of genomic and morphological data, the authors set out to estimate the timing, frequency, and biogeography of dietary shifts; test evolutionary rates associated with planktivory (i.e. speciation, extinction, and transitions); assess correlations between trophic transitions and paleoclimatic trends; and identify the potential genomic basis of selection for planktivory. Overall, I found this to be a very interesting, well written, and well executed manuscript. The dataset assembled is both comprehensive and valuable, and the analyses offer important insights into the evolutionary history of a key reef fish group. I commend the authors on the herculean effort of compiling and analysing such a large dataset, and I believe the manuscript will be of broad interest to ichthyologists and evolutionary biologists alike.
That said, I will limit my evaluation to the phylogenetic and macroevolutionary components of the study, as I do not have the necessary expertise to comment on the genomic analyses (e.g. signatures of selection and gene annotations). Below I outline both positive aspects and points that I think should be strengthened before publication.

Response: Thank you, Dr. Siqueira, for the positive and encouraging comments. We have addressed all concerns raised below.

Major points

One of the major strengths of this manuscript is the integration of morphological and molecular evidence to produce divergence time estimates. This approach provides more realistic age estimates for Acanthuriformes compared to earlier studies (including my own), where inferred crown ages were often older than supported by the fossil record. It is encouraging to see that combined datasets reconcile molecular and paleontological perspectives, in line with recent findings for other reef-associated lineages (e.g. Cantalice et al. 2022 – BioScience). This represents a significant advance in our understanding of reef fish evolution and helps resolve mismatches identified in earlier analyses. The manuscript therefore highlights the value of integrating fossil and extant species data, and I strongly support this approach.
Despite agreeing with most of the results and interpretations, I do have a few suggestions for improvement.

The first relates to the interpretation of the paleoclimatic model results. Based on Figure 1B and Table S8, the lambda model appears to be consistently better supported than climate-dependent models, both for the MCC tree and across the posterior sample of trees. If this is correct, the evidence does not support a strong role for temperature in driving evolutionary differences between feeding modes. Statements such as "…planktivory diversified during periods of global cooling" (lines 34-35) seem to be based largely on lineage-through-time plots, which in my view provide weak evidence compared to the formal model comparison. Unless I am misinterpreting the results (which I might), I recommend toning down or removing claims of a causal relationship between paleoclimatic shifts and trophic evolution, particularly in the abstract and conclusions. The paper already makes a substantial contribution without leaning on this inference, which currently feels unsupported by the analyses.

Response: The reviewer is correct that the lambda model was consistently better supported than the climate-dependent models, both for the MCC tree and across the posterior sample. We now emphasize that stronger support was recovered for a climate-independent (phylogenetic signal) model. We also revised the Abstract, Results, and Conclusions to reflect this balanced interpretation and removed any phrasing implying a strong causal link between paleoclimate and trophic evolution.

Abstract: "Time-dependent models support both climate and phylogenetic effects on planktivorous lineage diversification."

Conclusions: "Our findings suggest that dietary shifts reflect both paleoclimatic fluctuations and lineage-specific histories, with temporal patterns aligning planktivore diversification with cooler intervals and benthic herbivore diversification with warmer periods, while models also support strong phylogenetic influences."

Related to this, it would also be useful to better integrate the results of the transition models. Based on the simmap analysis, the equal-rates model of transition between dietary states is preferred. However, when discussing whether planktivory is an evolutionary dead end, the interpretation relies mainly on the HiSSE model results, which show higher transition rates from non-planktivores to planktivores. As the results acknowledge, this pattern is largely driven by transitions and reversals in the genus Naso. The two approaches seem to suggest different dynamics, yet the manuscript does not fully reconcile these outcomes. While I agree with the overall conclusion that planktivory does not appear to be an evolutionary dead end in Acanthuriformes, I suggest more explicitly discussing why simmap and HiSSE may yield different inferences, and what this means for the robustness of the conclusions.

Response: We agree that clarifying the relationship between the SIMMAP and HiSSE results strengthens the interpretation of trophic evolution. In the SIMMAP analyses, the equal-rates (ER) model was marginally favored (AICw = 0.55 with fossils; 0.51 without fossils), but the all-rates-different (ARD) model also received substantial support (AICw = 0.45 with fossils; 0.48 without fossils), indicating limited power to confidently discriminate between symmetric and asymmetric transitions. Importantly, estimated transition rates under both SIMMAP models are highly consistent with those inferred by HiSSE. Including fossils, SIMMAP estimated an ER rate of 0.015, with ARD rates of 0.012 from non-planktivory to planktivory and 0.025 for the reverse; excluding fossils, the ER rate was 0.012, with ARD rates of 0.010 and 0.025, respectively. These values closely match the HiSSE estimates (0.010 from non-planktivory to planktivory and 0.026 for the reverse). Thus, despite differences in model selection outcomes, both approaches recover the same asymmetric pattern, largely driven by repeated reversals within Naso. The two methods differ primarily in scope: SIMMAP provides a time-integrated view of transitions across fossil-calibrated phylogenies, whereas HiSSE more sensitively captures clade-specific dynamics in extant lineages. We now explicitly discuss these methodological differences and their implications in the Results and Discussion, emphasizing that both approaches converge on the same conclusion: planktivory is evolutionarily reversible and does not represent an evolutionary dead end in Acanthuriformes.

"Ancestral diet reconstruction using the *make.simmap* function in the R package *phytools*[6] (SIMMAP hereafter) was conducted under the best-fit equal-rates (ER) model for both phylogenies; ER was marginally favored for the extant-only tree (AICw = 0.51 vs. 0.48 for all rates different or ARD) and similarly for the tree including fossils (AICw = 0.55 vs. 0.45 for ARD). Under both models, we inferred six independent transitions to planktivory in the extant-only tree (Figs. S12, S15). When fossils were incorporated, the number of independent origins increased to at least seven (range: 7–9 across posterior distribution [PD] trees)."

"When we compared these results with transition rates from ancestral diet reconstructions, we found them to be highly consistent. Including fossils, SIMMAP estimated an ER rate of 0.015, with ARD rates of 0.012 from non-planktivory to planktivory and 0.025 for the reverse; excluding fossils, the ER rate was 0.012, with ARD rates of 0.010 and 0.025, respectively. These values closely match the HiSSE estimates (0.010 and 0.026), indicating that differences between approaches reflect model structure and sensitivity rather than conflicting evolutionary signals."

Finally, I believe some parts of the Results and Discussion section could be streamlined, as the manuscript is already quite long. For example, the opening paragraphs of the sections "No evolutionary dead end associated with the evolution of planktivory" and "Signatures of convergence in positive selection following transitions to planktivory" feel somewhat repetitive and read more like background introduction. Reducing or condensing these sections would improve readability and help the main findings stand out more clearly.

Response: We have reduced the opening paragraph of "No evolutionary dead end associated with the evolution of planktivory", by excluding this excerpt: "Adopting a water column lifestyle may offer short-term advantages, but in the long term, it can lead to an evolutionary trap, or 'evolutionary dead end', due to elevated extinction rates, reduced speciation, or both"

In addition, for the section "Signatures of convergence in positive selection following transitions to planktivory," we removed the first two sentences, which we agree were repetitive with information already presented in the Introduction. Specifically, we removed the following text:

"The repeated instances of convergence towards a planktivorous diet have been associated with specialized morphological traits, including variations in jaw size reduction, tooth morphology, elongated gill rakers, slender fusiform body shapes, and bifurcated caudal fins. These morphological adaptations have been documented not only in surgeonfishes[11] but also in cichlids[2], damselfishes[100], carangids[17], grunts[10], and snappers and fusiliers[9], among others."

Minor Points

Line 81: The parentheses is not closed. This also happens in other instances (e.g. lines 291, 578, 630), so I am assuming this stems from the automatic reference maker. Please double check throughout the text.

Response: All the parentheses have been closed.

Line 101: I suggest changing from "ecological histories" to just "ecologies" for simplicity.

Response: Changed.

Lines 106-107: I appreciate that my previous work relied on taxonomic placement of fossil species rather than the morphological dataset used here, but it was based on the available literature, not on "subjective interpretation of photographic records" as stated. I suggest rephrasing this to more accurately reflect the approach taken. In addition, the order of citations in this sentence appears to be incorrect (I believe ref. 50 rather than 51 is intended?), please double check.

Response: The citation to the sentence the referee is referring is not citing Siqueira et al 2019. It is referring to the reference 50 (now ref. 52), Bellwood et al 2014. However, we now included Siqueira et al 2019's work (ref. 51) and modified the sentence to: "Two studies—one including 18 fossil taxa subjectively placed from photographic records alongside 41 extant species [3], and another with 25 fossil taxa placed according to available literature alongside 72 extant species[4], rather than explicit morphological datasets—proposed that surgeonfishes originated in two distinct phases: first during the Paleocene–Eocene (~66–34 Ma), potentially due to ecological release after the Cretaceous–Paleogene (K–Pg) mass extinction, and again in the Oligocene–Miocene (~23–5 Ma), coinciding with the rise of extensive coral reef systems [5]."

Line 108: Suggested change from "originated" to "diversified" if the authors agree.

Response: Agreed. Change has been made.

Lines 123-127: Perhaps numbering the specific goals might help with clarity; e.g. "Specifically, we 1) estimated…; 2) tested…; 3) assessed…; 4) identified…"

Response: We numbered the goals as suggested.

Line 132: Suggestion - "…acanthuriform trophic diversity has been shaped…"

Response: We have made a similar change: "trophic shifts have been shaped."

Lines 146-148: I suggest breaking this sentence in two; e.g. "…and 9 outgroups. Of these…"

Response: Changed.

Line 152 (and throughout the results): Considering the amount of supplementary material, it might be a good idea to order the supp figures in order of appearance in the main text. The current order made it hard to scroll back and forth while reading the main text.

Response: We appreciate this suggestion. The SM figures are ordered to reflect the analytical workflow rather than their order of appearance in the main text, which we chose to preserve for clarity regarding the progression of analyses. We did change the order of figures S2 and S3. All figures are clearly referenced and labeled in the manuscript, and we therefore retained the current ordering.

Lines 157-158: I thought this was super interesting that the morphological dataset groups Luvarus with the (mostly planktivorous) Naso. Could this be an indication of how morphological convergence towards planktivory might mislead the analysis and, therefore, shows how important it is to also include the genetic data? Just a thought.

Response: The reviewer raised an excellent point. Accordingly, we added a sentence addressing this interpretation of our results. "By contrast, morphology-only trees generated with TNT v1.5 [7] split Zanclidae and Acanthuridae and reposition Luvaridae, indicating that skeletal traits alone can be misleading at this phylogenetic depth (Fig. S6). Specifically, the morphology-only dataset groups *Luvarus* with the largely planktivorous *Naso*, a pattern that may reflect convergence associated with planktivory and illustrates how morphology alone can obscure true evolutionary relationships, reinforcing the importance of incorporating genomic data."

Lines 169-170: This is a massive amount of time (well done on the effort!) and yet, some subsets did not achieve the desired ESS. From my experience, adding the fossils makes it hard for the analysis to converge. Just to be clear, I am in no way suggesting that you should rerun the analyses; but just wondering out of curiosity if you compared an analysis with and without the fossils to see if convergence would be achieved faster?

Response: Thank you for acknowledging our effort. We did not run MrBayes analyses without fossils. Based on previous experience, MrBayes runs substantially faster when fossils and a morphological matrix are not included. However, once a morphological matrix is added, especially one like ours with 107 characters, runs become much slower. In fact, even though such datasets typically converge faster than tip-dating analyses with fossils, our current run (which has been running for about a month while preparing this review) has still not reached convergence, with some ESS values remaining between 20 and 50.

Line 200: "diets" instead of "lifestyle"?

Response: We removed "lifestyle," but did not add "diets" since the word is already used in the sentence: "With the newly inferred time trees including fossil and living acanthuriform species, we reconstructed their diets to investigate the number of times the planktivory evolved, as well as the regions and timing of these transitions."

Lines 269-272: Yes, I agree that this result is mostly being driven by Naso. Therefore, it might be relevant to note that some Naso species (specifically Naso brevirostris) browse on benthic macroalgae as juveniles and only then transition to being more planktivore (see Clements 1991 for example). Perhaps this demonstrates how diet in this genus may be more labile than usual?

Response: We added a sentence clarifying the dietary lability in unicornfishes as follows: "Some species in this genus, especially *N. brevirostris* and *N. vlamingii*, feed on benthic macroalgae as juveniles and become planktivorous later in life 84,85, highlighting an exceptional dietary lability of *Naso*.

Lines 273-276: This sentence is a little awkward, can you please clarify?

Response: We changed it to: "While planktivory is often considered an evolutionary destination[15], some unicornfishes revert to their ancestral trophic states without further dietary specialization, representing a notable exception to this expectation."

Lines 296-297: Where does the information that fishes on the Paleocene fed on "filamentous or macrophytic algae as well as detritus and microbes" come from? Just curious.

Response: We have now clarified this further: "particularly benthic lineages whose trophic ecology (based on inference from extant relatives [9]) involved filamentous or macrophytic algae as well as detritus and microbes."

Line 305: How do you know that benthic-feeding lineages went extinct at this time? The fossil-tip analysis only suggests these lineages were present at that time, but not necessarily when they went extinct. Please correct me if I am wrong.

Response: Fair point. We have now rephrased the sentence as follows: "Several benthic-feeding acanthuriform lineages are restricted to this interval in the fossil record (Fig. 1A; [47,48]), suggesting a contraction or turnover of benthic trophic lineages during this time."

Line 311: Suggestion "However, it is important to recognize that strong taphonomic biases…"

Response: We have now revised the sentence as suggested.

Line 317: In this case, are you talking about speciation rates or just number of lineages through time? Please clarify.

Response: We were talking about the number of lineages through time. We have now clarified the sentence as follows: "A second wave of diversification occurred in the early Miocene (~20 Ma), when acanthuriform planktivores, alongside other reef fishes, increased in lineage diversity, although non-planktivores exhibited a steeper diversification slope (Fig. 1A)."

Line 360: "estimated" instead of "estimationed".

Response: Changed.

Materials and Methods: Some of the sections in the Supplementary Materials seem almost the same (with a little bit more detail) as in the main text. Is there a need for these two versions? My suggestion would be to consolidate the important information in the main text, and only have auxiliary info (e.g. the list of morphological characters, tables and figures) as supplementary.

Response: We have now removed the redundant information from the Supplementary Material.

Lines 614-621: Sorry if I am missing something here, but these numbers are not matching. In the previous methods section, you talk about 56 in-group species sequenced, but here 91 newly

sequenced species are mentioned. Then at the end you talk about 80 in-group species and 9 outgroup species and in parenthesis there are 90 species in total. Please double check these numbers as they are confusing.

Response: We did not sequence 91 species. We sequenced 66 specimens (57 ingroup including A. tractus + 9 outgroups). We now fixed all the numbers in the following sentence:

"After implementing a set of quality control steps, the final reduced molecular matrix assembled comprised 998 genes for 57 ingroup taxa (56 species out of the 86 extant acanthuriforms), representing all genera and families, along with 9 outgroup species as outlined above."

"To increase the number of species, we generated an expanded matrix combining the 66 newly sequenced taxa (reduced matrix) with 59 species obtained from GenBank."

"The final expanded molecular matrix comprises 1,002 genes for 80 ingroup species (~93%) and 9 outgroup species (65 spp. with FishLife exons and 24 spp. with legacy markers only)."

Line 647: Should it be "see Supplementary material for the list…"?

Response: Changed.

Lines 688-690: Suggested rephrasing: "Because these analyses ran for over a year and Subset 2 based on the Scheme 2 had not reached satisfactory ESS values, we removed it from all downstream analyses."

Response: We rephrased the sentence as suggested.

Line 691: I am assuming the trees were sampled from the posterior distribution post-burnin, right?

Response: The reviewer is correct. We added post-burn-in for clarification.

Lines 690-693: Out of curiosity, if you combine the posterior distributions from these subsets, do you get ESS above 200? This is essentially what your sample of the posterior represents (the 10,000 trees per Scheme).

Response: When I combine all the posterior distributions from the five subsets for both schemes, the ESS are above 200. See screenshots from TRACER below:

Scheme 1:

Trace Files:

Trace File	States	Burn-In
MrBayes_05subsets_04_SAcanthuriformes.r...	385900000	38590000
MrBayes_05subsets_04_SAcanthuriformes.r...	385900000	38590000
MrBayes_05subsets_04_SAcanthuriformes.r...	385900000	38590000
MrBayes_05subsets_04_SAcanthuriformes.r...	385900000	38590000
MrBayes_05subsets_04_SAcanthuriformes.r...	385900000	38590000
MrBayes_05subsets_04_SAcanthuriformes.r...	385900000	38590000
MrBayes_05subsets_04_SAcanthuriformes.r...	385900000	38590000
MrBayes_05subsets_04_SAcanthuriformes.r...	385900000	38590000
MrBayes_05subsets_05_SAcanthuriformes.r...	333710000	33371000
MrBayes_05subsets_05_SAcanthuriformes.r...	333710000	33371000
MrBayes_05subsets_05_SAcanthuriformes.r...	333710000	33371000
MrBayes_05subsets_05_SAcanthuriformes.r...	333710000	33371000
MrBayes_05subsets_05_SAcanthuriformes.r...	333710000	33371000
MrBayes_05subsets_05_SAcanthuriformes.r...	333710000	33371000
MrBayes_05subsets_05_SAcanthuriformes.r...	333710000	33371000
MrBayes_05subsets_05_SAcanthuriformes.r...	333710000	33371000
Combined	13292800000	-

+ − Reload

Traces:

Statistic	Mean	ESS	Type
LnL	-4.06E5	1570	R
LnPr	-743.627	1278	R
TH{all}	7.51E-2	903	R
TL{all}	1.385	1175	R
prop_ancfossil{all}	0.162	3143	R
r(A<->C){1}	7.235E-2	404	R
r(A<->G){1}	0.191	335	R
r(A<->T){1}	9.335E-2	619	R
r(C<->G){1}	2.398E-2	344	R
r(C<->T){1}	0.573	336	R
r(G<->T){1}	4.589E-2	411	R
r(A<->C){2}	7.474E-2	555	R
r(A<->G){2}	0.317	337	R
r(A<->T){2}	6.567E-2	361	R
r(C<->G){2}	0.102	340	R
r(C<->T){2}	0.392	336	R
r(G<->T){2}	4.929E-2	411	R
r(A<->C){3}	6.509E-2	521	R
r(A<->G){3}	0.364	346	R
r(A<->T){3}	8.53E-2	345	R
r(C<->G){3}	4.953E-2	335	R
r(C<->T){3}	0.383	568	R
r(G<->T){3}	5.286E-2	846	R
pi(A){1}	0.252	336	R
pi(C){1}	0.267	340	R
pi(G){1}	0.314	352	R
pi(T){1}	0.166	336	R
pi(A){2}	0.308	341	R
pi(C){2}	0.237	350	R
pi(G){2}	0.212	335	R
pi(T){2}	0.244	335	R
pi(A){3}	0.205	335	R
pi(C){3}	0.293	337	R
pi(G){3}	0.273	335	R
pi(T){3}	0.229	343	R
pi(A){4}	0.283	10104	R
pi(C){4}	0.275	8399	R
pi(G){4}	0.194	10529	R
pi(T){4}	0.248	9749	R
alpha{1}	0.254	334	R
alpha{2}	4.107E-2	357	R
alpha{3}	0.675	335	R
alpha{4}	5.118E-2	14343	R
m{1}	1.102	335	R
m{2}	0.394	334	R
m{3}	1.491	334	R
m{4}	1.016	2297	R
m{5}	4.608	1879	R
net_speciation{all}	3.111E-2	17917	R
relative_extinction{all}	0.823	7984	R
relative_fossilization{all}	8.013E-2	5002	R
igrvar{all}	3.346E-3	2129	R
clockrate{all}	1.178E-3	904	R

Scheme 2:

Trace Files:

Trace File	States	Burn-In
MrBayes_05subsets_04_SAcanthuridae.r...	349050000	34905000
MrBayes_05subsets_04_SAcanthuridae.r...	349050000	34905000
MrBayes_05subsets_04_SAcanthuridae.r...	349050000	34905000
MrBayes_05subsets_04_SAcanthuridae.r...	349050000	34905000
MrBayes_05subsets_04_SAcanthuridae.r...	349050000	34905000
MrBayes_05subsets_04_SAcanthuridae.r...	349050000	34905000
MrBayes_05subsets_04_SAcanthuridae.r...	349050000	34905000
MrBayes_05subsets_04_SAcanthuridae.r...	349050000	34905000
MrBayes_05subsets_05_SAcanthuridae.r...	360500000	36050000
MrBayes_05subsets_05_SAcanthuridae.r...	360500000	36050000
MrBayes_05subsets_05_SAcanthuridae.r...	360500000	36050000
MrBayes_05subsets_05_SAcanthuridae.r...	360500000	36050000
MrBayes_05subsets_05_SAcanthuridae.r...	360500000	36050000
MrBayes_05subsets_05_SAcanthuridae.r...	360500000	36050000
MrBayes_05subsets_05_SAcanthuridae.r...	360500000	36050000
MrBayes_05subsets_05_SAcanthuridae.r...	360500000	36050000
Combined	10792640000	-

+ − Reload

Traces:

Statistic	Mean	ESS	Type
LnL	-4.024E5	752	R
LnPr	-746.012	932	R
TH{all}	7.425E-2	604	R
TL{all}	1.372	812	R
prop_ancfossil{all}	0.166	2040	R
r(A<->C){1}	7.432E-2	400	R
r(A<->G){1}	0.151	456	R
r(A<->T){1}	9.489E-2	958	R
r(C<->G){1}	2.098E-2	1168	R
r(C<->T){1}	0.615	398	R
r(G<->T){1}	4.41E-2	685	R
r(A<->C){2}	7.473E-2	413	R
r(A<->G){2}	0.355	784	R
r(A<->T){2}	6.061E-2	503	R
r(C<->G){2}	0.12	370	R
r(C<->T){2}	0.337	398	R
r(G<->T){2}	5.259E-2	783	R
r(A<->C){3}	6.474E-2	361	R
r(A<->G){3}	0.376	334	R
r(A<->T){3}	9.106E-2	307	R
r(C<->G){3}	3.396E-2	348	R
r(C<->T){3}	0.381	410	R
r(G<->T){3}	5.309E-2	468	R
pi(A){1}	0.267	410	R
pi(C){1}	0.259	323	R
pi(G){1}	0.319	400	R
pi(T){1}	0.155	460	R
pi(A){2}	0.315	308	R
pi(C){2}	0.234	284	R
pi(G){2}	0.188	530	R
pi(T){2}	0.263	327	R
pi(A){3}	0.181	321	R
pi(C){3}	0.305	383	R
pi(G){3}	0.291	318	R
pi(T){3}	0.223	322	R
pi(A){4}	0.283	3983	R
pi(C){4}	0.275	6540	R
pi(G){4}	0.195	4112	R
pi(T){4}	0.248	5922	R
alpha{1}	0.134	465	R
alpha{2}	2.138E-2	1500	R
alpha{3}	0.826	355	R
alpha{4}	5.095E-2	6371	R
m{1}	0.968	390	R
m{2}	0.26	308	R
m{3}	1.759	318	R
m{4}	1.021	1456	R
m{5}	4.629	1256	R
net_speciation{all}	3.11E-2	7691	R
relative_extinction{all}	0.822	4543	R
relative_fossilization{all}	8.086E-2	3136	R
igrvar{all}	3.272E-3	1653	R
clockrate{all}	1.165E-3	602	R

Type: (R)eal (I)nt (C)at (T)ime * constant

Lines 700 and 717: Perhaps cite the literature sources?

Response: We added the following references: Tebbett et al. 2022 and FishBase.

Lines 758-762: "…rates independent of…" and "…dependent on…"

Response: Changed.

Figure S8: A part of legend is repeated, please double check.

Response: The repeated sentence of the legend was removed.

REVIEWER COMMENTS

Reviewer #1 and #2 (Remarks to the Author):

We appreciate the author's thoroughness in addressing all our comments and in taking the time to rerun the BUSCO analyses on the updated dataset. We believe this study to be very interesting and important to better understand the evolutionary history and diversification of reef fishes. This final version is much clearer and more concise, and we are happy with all the revisions done. We suggest this manuscript for acceptance.
Response: We thank the reviewers for their constructive and positive comments, which have helped improve the robustness and clarity of the manuscript.

Reviewer #3 (Remarks to the Author):

I thank the authors for considering and addressing my comments from the first round of reviews. I have checked the responses to all reviewers and consider them satisfactory. The manuscript is substantially improved and will be a valuable addition to the literature.
Response: We thank the reviewer for their constructive feedback. His comments and suggestions have substantially strengthened the manuscript.

I only have a few very minor points to suggest:

- Line 203 of the ms version after accepting all changes: remove "the" from the sentence "With the newly inferred time trees including fossil and living acanthuriform species, we reconstructed their diets to investigate the number of times the planktivory evolved, as well as the regions and timing of these transitions".
  Response: We removed "the" as suggested: "With the newly inferred time trees including fossil and living acanthuriform species, we reconstructed their diets to investigate the number of times planktivory evolved, as well as the regions and timing of these transitions."

- Line 276: "highlighting an exceptional dietary lability in Naso".
  Response: We changed "of" to "in".

- Line 300-303: Detritus feeding has been suggested as a more recent acquisition (see Bellwood et al. 2014 Nat Comms), so unless there is other fossil evidence to indicate this type of feeding in the Eocene, I suggest changing the sentence to: "These analyses revealed that acanthuriforms gradually started diversifying during the Paleocene and early Eocene (66–50 Ma), particularly benthic lineages whose trophic ecology (based on inference from extant relatives 11) might have involved filamentous or macrophytic algae."
  Response: We added "might have" to the sentence as suggested.

- Line 327: I suggest adding "potential" to the sentence "Although we observe a potential temporal correlation between the evolution of planktivory and major climatic transitions, our model fitting analyses indicate split support between climate-dependent and climate-

independent models".
Response: We added "potential" as suggested.

- Line 374: "Understanding whether trophic transition convergence is mirrored at the molecular level…", change transition to singular?
Response: We changed transition to singular.

- Lines 405-406: suggested change "Among these, 39 convergently underwent positive selection in at least two lineages (Fig. 3 and Table S11)".
Response: We removed "of them" from the sentence as suggested.

Santaquiteria et al. Ecological and genomic signatures of the convergent evolution of planktivory in fossil and living fishes over deep time.

In this study, Santaquiteria et al present a comprehensive study that aims to investigate the evolution of planktivory in Acanthuriformes (specifically focused on surgeonfishes, unicornfishes, tangs, luvars, and moorish idols) while also testing the hypothesis that pelagic planktivory represents an evolutionary dead end. This study integrates genomic and morphological data across more than 80 species encompassing approximately 93% of the group's diversity, representing one of the most complete phylogenomic studies of Acanthuriformes to date. Among their most notable results, they find evidence that rejects the hypothesis of pelagic planktivory as an evolutionary dead end, and find shared molecular pathways associated with shifts in trophic positions that evolved by parallel or convergent evolution.

Overall, the manuscript is well written and will serve as an important study to enhance our understanding of the ecological and genetic drivers shaping dietary evolution in marine fishes. With the declining costs of sequencing, more studies will benefit from this framework to combine morphological and genomic data to better understand the drivers of diversification and obtain higher resolution phylogenies. The methods and data provided are thorough and clear. However, the methods provided in the manuscript and supplementary information greatly overlap and could possibly be updated in the main text to reduce the supplementary information if the word count allows.

Specific comments:

- Scheme 1 versus Scheme 2 should be introduced with more clarity – it took a few reads to figure out the difference and the best explanation came from the methods (section: *Total-evidence dating analyses and phylogenetic uncertainty*)

- We suggest going into more depth on the reasoning behind pelagic planktivory as an "evolutionary dead end" (~Line 67-73) in the introduction. This is better described later in the paper (Line 236-243) and would be useful to move this earlier or expand in the introduction.

- Line 141-144: The math doesn't seem to add up in the number of species. You mention there are "80 ingroup species" and "9 outgroup" which would be a total of 89 species, however, you follow up with "of these, 65 species have data for 998 'FishLife' exon markers 54, which also incorporate 22 legacy markers, whereas 25 species include data only for legacy markers". 65 + 25 would be a total of 90 species, not 89? Similarly, in Line 599 you mention "91 newly sequenced species". Check the numbers as they are not consistent.

- We suggest to rerun BUSCO with the updated actinopterygii dataset (used odb9 but odb12 is available). This should not be too time consuming but would make it stronger and more up to date.

- Line 395: You mention *Naso* exhibited the highest number of shared PSG's with other planktivors, however in Figure 3 it seems to be only one *Naso* species (*Naso mcdadei*). We suggest discussing this further, or clarifying whether the UpSet plot of Figure 3 refers to Scenario 1 specifically.

- Figure 1: (Panel A)
    - Black and Red lines are described in Panel B but should be moved to Panel A.
    - Phylogeny tips are a bit hard to see with the green and blue line going through them. We suggest making the tips slightly larger to adequately see the tip colors.

- Figure 2:
    - This figure is not very clear. Figure caption could use more detail. Colors in the key below the figure don't seem to fully match the phylogeny or those shown on the black bars. Blue colors in general are very similar and hard to tell apart. The colors overlaid on the map are hard to see as well. It is unclear what the key at the bottom of the figure is intended to illustrate. Lastly, "CIP" is missing from the figure caption.

- Figure 3:
    - Break this figure up into panel A, B, and C. Within the text you refer to blocks of this figure, but it is not aligned with how the figure is presented
    - Line 853: change "Fig. 3A" to "Fig 3", OR alter Figure 3 as in comment above.
    - Line 866: change "Fig. 3D" to "Fig 3", OR alter Figure 3. However, we're not sure what this is referring to in figure 3. Or did you mean Figure 4?

- Figure 4:
    - Similar to the above comment, break it up in panels A and B to keep it consistent.
    - Although we appreciate the image of the fish, was there a specific reason this species was selected? We suggest mentioning its importance or presence in the caption.

Minor Comments:

- Line 81: add ")" after "but see 25-27"
- Line 105-110: Long confusing sentence. Suggest splitting and rewording.
- Lines 265-267: reword sentence: "While planktivory is considered an evolutionary destination, **some** unicornfishes, despite not further specializing their diet, can revert to their ancestral trophic states…"
- Lines 374-375: Is this sentence stating BUSCO scores run in both genome and protein modes? If so, we recommend rewording to say so instead of describing the protein BUSCO as a "decrease" from the initial assembly BUSCO, as these are two separate analyses.
- Line 615: add ")" after "supercomputers"
- Line 616: change "bootstrapping" to "bootstrap replicates"
- Line 638: change "bootstraps" to "bootstrap replicates"
- Lines 651-652: reword "combining molecules with morphology" to something like "a combination of morphology and molecular information"
- Line 670: take "of the 10" out of parentheses
- Lines 860-862: reword this sentence to something like "aBSREL is restricted to focal branches and therefore cannot detect whether a gene is also under selection in non-focal branches, so we complemented these analyses with BUSTED-Phenotype (BUSTED-PH)."
